# Pd/Ag-Cocatalyzed Merging Intramolecular Oxidative Coupling and Cascade [4 + 2] Cycloaddition: Synthesis and Photophysical Properties of Novel Polycyclic *N*-Heterocycles Fused Naphthoquinones

**DOI:** 10.3390/molecules29235639

**Published:** 2024-11-28

**Authors:** Yu Dong, Lin Chen, Han-Qing Wu, Li Xie, Jing-Hao Yu, Fan Yang, Yu-Ting Wang, Yu-Rong Liu, Guo-Wei Deng, Zhi-Fan Wang

**Affiliations:** 1 Sichuan Provincial Key Laboratory for Structural Optimization and Application of Functional Molecules, College of Chemistry and Life Science, Chengdu Normal University, Chengdu 611130, China; ssdy1990@126.com (Y.D.); cl13649000476@163.com (L.C.); 18070442587@163.com (J.-H.Y.); 18153805241@163.com (F.Y.); yutingwang0919@126.com (Y.-T.W.); 18884569093@163.com (Y.-R.L.); 2 Chengdu Institute for Drug Control, Chengdu 610061, China

**Keywords:** Pd/Ag-cocatalyzed, intramolecular oxidative coupling, [4 + 2] cycloaddition, novel polycyclic *N*-heterocycles, photophysical properties

## Abstract

We report a step-economic strategy for the direct synthesis of novel polycyclic *N*-heterocycle-fused naphthoquinones by merging intramolecular oxidative coupling and cascade [4 + 2] cycloaddition. In the protocol, mechanistic investigations suggest that the cascade reaction involves the intermediate spiro polycyclic *N*-heterocycles and [4 + 2] cycloaddition processes. This protocol is featured with moderate to excellent yields, wide substrate scope, and divergent structures of products. In addition, the photophysical properties of the synthesized products were evaluated. These products exhibit interesting fluorescence properties, and surprisingly, the compounds have the ability to selectively recognize trifluoroacetic acid.

## 1. Introduction

**Figure 1:**
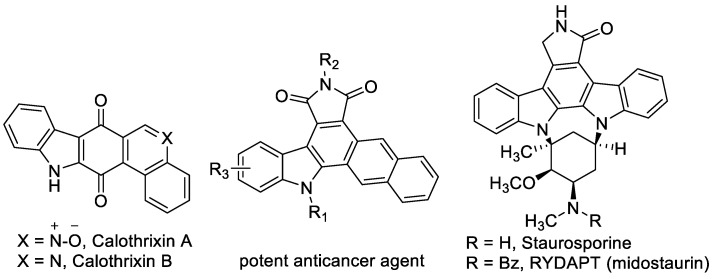
The representative examples of polycyclic *N*-heterocycles.

*N*-Heterocycle-fused polycyclic organic scaffolds are privileged structural motifs that frequently occur in various, drugs, natural products, and functional materials [1,2,3,4,5,6,7]. The representative examples of the quinone-fused polycyclic *N*-heterocycles Calothrixin A and B both inhibited the in vitro growth of the chloroquine-resistant strain of the human parasite, *Plasmodium falciparum*, in a dose-dependent manner [8,9]. In addition, staurosporine (kinase inhibitor) [10], a potential candidate for cancer treatment [11], and midostaurin (FDA-approved drugs) were approved as drugs to treat patients diagnosed with a form of blood cancer known as acute myeloid leukemia in 2017 (Figure 1) [12,13]. In addition, some polycyclic compounds containing the basic structure, which are complex molecules, are privileged scaffolds that possess diverse applications in organic electronics [14,15] and a wide range of biological activities [16,17,18,19].

Annellated polycyclic complexes bearing a quinone skeleton are very promising for applications in various areas [20,21,22,23]. However, there are a few related reports on the construction of quinone-fused polycyclic *N*-heterocycles. The D–A addition reaction is one of the efficient strategies for constructing cyclized derivatives from naphthoquinones. For instance, in 1990, the Pindur group reported the first structural example of indolocarbazole-fused naphthoquinone by a Diels–Alder reaction of naphthoquinones and 3-vinylindole in only 10% yield [24]. In 2019, Fu’s group developed a formal [4 + 2] annulation of 2-methyl-3-oxoacetate indoles with naphthalene-1,4-dione [25]. In 2021, Wang’s research groups published B(C_6_F_5_)_3_-catalyzed three-component tandem reaction of naphthoquinone, indole, and maleimide [26]. In addition, the cycloaddition reaction is one of the effective methods for building complex cyclic structures from substituted naphthoquinones. For instance, in 2010, Zhang’s group reported a strategy to synthesize anthraquinone from bromoquinone and arylpropanoic acid [27]. In 2019, Albrecht’s group reported a [6 + 4]-cycloaddition reaction of 2-substituted 1,4-naphthoquinones with 8,8-dicyanoheptafulvene [28]. The cycloaddition reaction is also a classic strategy from indolenaphthoquinones. For instance, in 2020, Wang’s research groups realized the Co(II)-catalyzed cyclization reaction of indolequinones and aniline [29]. In 2023, they also reported the B(C_6_F_5_)_3_-catalyzed [4 + 2] cyclization/oxidative reaction of indolequinones and dimethyl butynedioates [30]. Despite their merits, many existing synthetic approaches have several shortcomings, such as the preparation of the prerequisite functional groups, multiple reaction steps, harsh reaction conditions, expensive catalysts, and low product yields.

**Scheme 1:**
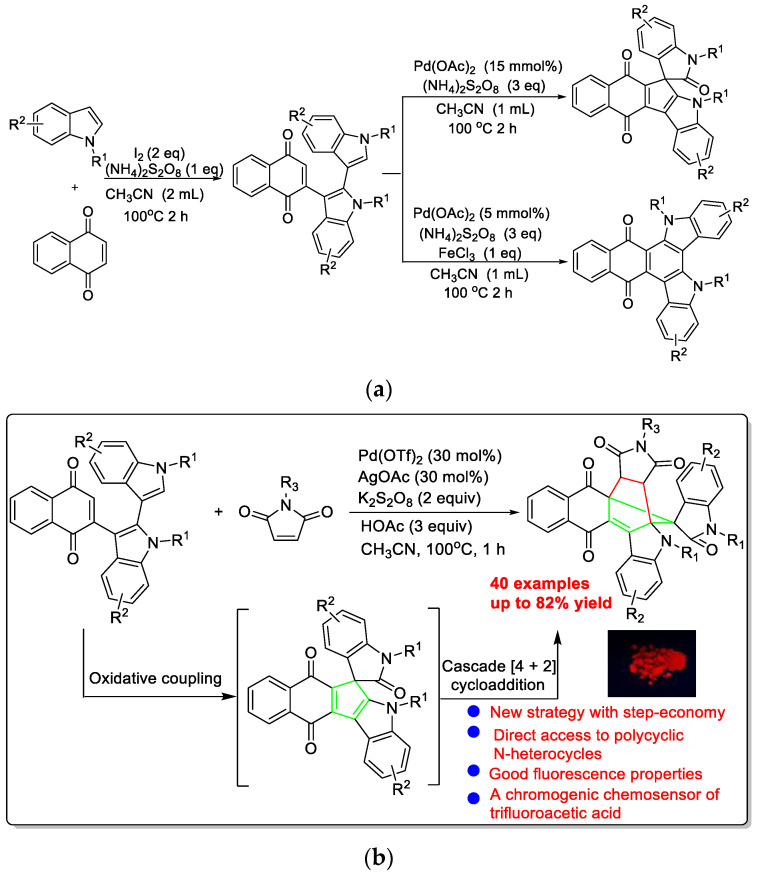
The synthesis of novel polycyclic *N*-heterocycle-fused naphthoquinones. (**a**) Our previous work [31]. (**b**) This work.

In order to develop better methods, we have developed a strategy for the synthesis of polycyclic *N*-heterocycle- and indolecarbazole-fused naphthoquinones by merging oxidative coupling and cascade palladium-catalyzed intramolecular oxidative cyclization from bi-indolylnaphthoquinones (Scheme 1a) [31]. Herein, we report a step-economic strategy for the direct synthesis of novel polycyclic *N*-heterocycle-fused naphthoquinones by merging intramolecular oxidative coupling and cascade [4 + 2] cycloaddition. In the protocol, mechanistic investigations suggest that the cascade reaction involves the intermediate spiro polycyclic *N*-heterocycles and [4 + 2] cycloaddition processes (Scheme 1b).

## 2. Results

### 2.1. Synthesis

We commenced our investigation of oxidative coupling using bi-indolnaphthoquinone **1a** and maleimide **2a** as model substrates with the aim of obtaining the novel polycyclic *N*-heterocycle **3aa** (Table 1). Next, we proceeded to investigate if the merging intramolecular oxidative coupling and cascade [4 + 2] cycloaddition process of bi-indolnaphthoquinones gave the intermediate spiro polycyclic *N*-heterocycle and maleimide **2a** (Table 1). Other palladium catalysts were less effective than Pd(OTf)_2_ (entry 2; Supplementary Materials). Moreover, other catalysts and other oxidants were less effective than AgOAc and K_2_S_2_O_8_, respectively (entries 3 and 4; Supplementary Materials). Changing their composition led to low yields of **3aa** (entry 5; Supplementary Materials), suggesting that the additives create suitable conditions for the cascade [4 + 2] cycloaddition. Switching the solvent and decreasing the reaction temperature was also ineffective in improving the yield of **3aa** (entries 6–7; Supplementary Materials). After rigorous optimization studies, we found the initial conditions to be optimal (Table 1, entry 1), providing the product **3aa** in 82% yield.

With the optimized conditions in hand, now, the scope of merging intramolecular oxidative coupling and cascade [4 + 2] cycloaddition process was explored, and the results are summarized in Table 2. First, we examined the scope of maleimides **2** by using **1a** as the partner. Various *N*-alkyl-substituted maleimides such as –Me (**2a**), –Et (**2b**), –Pr(**2c**), –nBu (**2d**), –iBu (**2e**), –tBu (**2f**), *n*-pentyl (**2g**), *n*-hexyl (**2h**), cyclopropyl (**2i**), cyclopentyl (**2j**), and cyclohenxyl (**2k**) all reacted smoothly with bi-indolnaphthoquinone **1a** to provide their corresponding polycyclic *N*-heterocycle products (**3aa**–**3ak**) in good yields (53–82%). The structure of **3ae** was determined using single-crystal X-ray diffraction analysis (Table 2). *N*-unsubstituted maleimides (**2l**) successfully provided the desired product **3al** (63%). Similarly, substituted *N*-benzylmaleimide (**2m**-**2u**) and ethylbenzene (**2v**) reacted well under this protocol to deliver the corresponding products **3am**-**3av**, respectively, in good yields (57–81%). Further, maleimides derived from 2-ethoxyethyl amine (**2w**), thiophenemethyl amine (**2x**), thiophenethyl amine (**2y**), and 5-aminoindole (**2z**) all successfully gave their polycyclic *N*-heterocycle products **3aw**–**3az** (in 53–79% yields).

Next, the merging intramolecular oxidative coupling and the cascade [4 + 2] cycloaddition reaction of bi-indolnaphthoquinone **1** that gave the intermediate spiro polycyclic *N*-heterocycle and maleimide **2** was investigated, and the results are listed in Table 3. *N*-alkyl-substituted indoles, such as Et, readily underwent the cascade [4 + 2] reaction to product **4a** in yields of 78%. The reaction was compatible with a variety of indole moieties bearing electron-donating and electron-withdrawing substituents to produce the desired polycyclic *N*-heterocycle products (**4b**–**4n**) in moderate to good yields (52–78%). The structure of the polycyclic *N*-heterocycle **4i** was confirmed by X-ray analysis. To our delight, the reaction also showed good compatibility with a wide range of valuable functional groups such as fluoro (**4c** and **4m**), chloro (**4d**, **4f**, and **4n**), and bromo (**4g**). Tolerance to the halogen atoms was noteworthy since they have been frequently used for further modification. Moreover, we were pleased to find that the position of the substituent on the indole moiety showed no obvious influence on the reaction outcome, and substitutions at the C5, C6, or C7 were all well tolerated in the cascade [4 + 2] cycloaddition reaction.

**Scheme 2:**
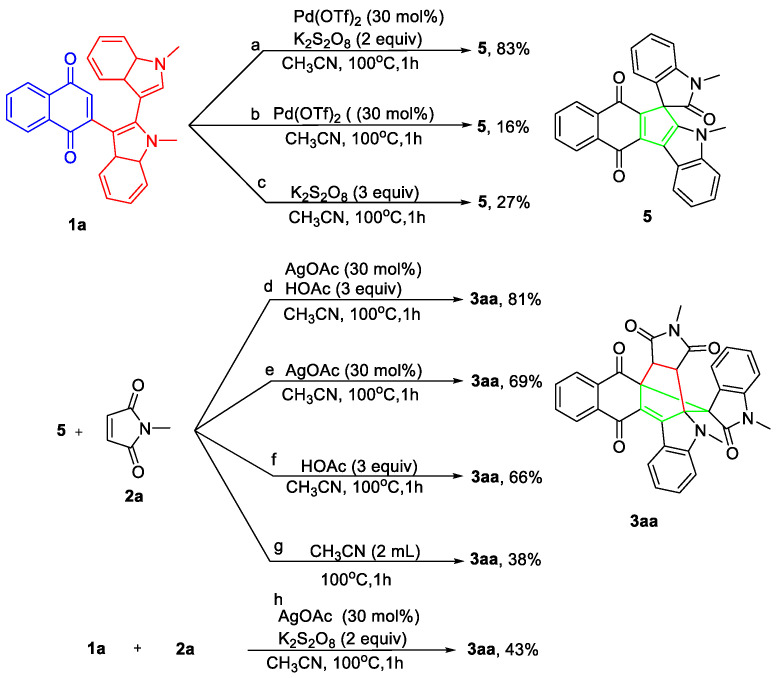
Control experiments for obtaining polycyclic *N*-heterocycles.

The theoretical calculation part was completed by the Beijing density function (BDF) program (2024A) (Figure S1 in Supplementary Materials) [32]. The studies demonstrated the importance of K_2_S_2_O_8_ and Pd(OTf)_2_. Under the conditions of AgOAc and HOAc, the reaction of cyclization product intermediate **5** and **2a** generated the polycyclic *N*-heterocycle product **3aa** in a yield of 81% (Scheme 2, (d)). We surmised that the cyclization product **5** should be a key intermediate in this reaction. In the absence of AgOAc or HOAc, the reaction of compound **5** and **2a** generated the polycyclic *N*-heterocycle product **3aa** in just 69%, 66%, and 38% yield (Scheme 2, (e–g)). The studies demonstrated the importance of AgOAc and HOAc. A control experiment involved using AgOAc without Pd for substrates **1a** and **2a**, and the reaction of the polycyclic *N*-heterocycle product **3aa** in a yield of 43% (Scheme 2, (h)). The studies demonstrated the importance of AgOAc and Pd(OTf)_2_.

**Scheme 3:**
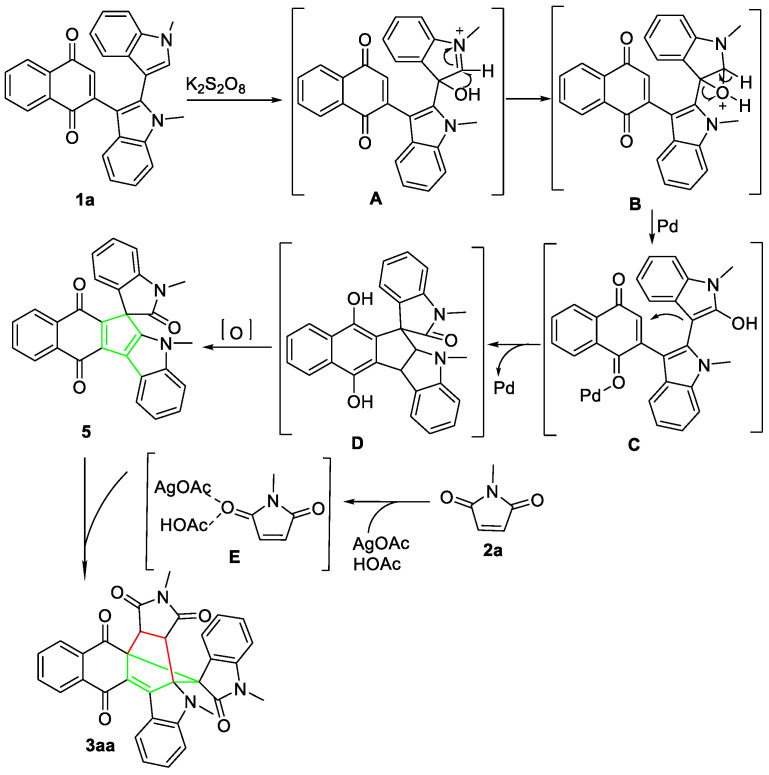
Plausible reaction mechanism.

On the basis of literature precedents and control experiments, a plausible mechanism is proposed in Scheme 3 [25,26,31,33,34,35]. Initially, the attack of the OH^+^ ion of (NH_4_)_2_S_2_O_8_ onto the nucleophilic center C-3 of indole (**1a**) generates enol intermediate (**C**) in situ through the intermediate compounds **A** and **B**. Palladium-catalyzed intramolecular nucleophilic attack of enol intermediate **C** form hydroquinone intermediate **D**. Then, the hydroquinone intermediate **D** further oxidized to spiro-cyclized polycyclic *N*-heterocycle products (5) by (NH_4_)_2_S_2_O_8_. At the same time, *N*-methylmaleimide (**2a**) is activated by AgOAc and HOAc, making intermediate **E** undergo the cascade [4 + 2] cycloaddition reaction with intermediate **5** to produce the final product **3aa**.

### 2.2. Photophysical Properties

**Figure 2:**
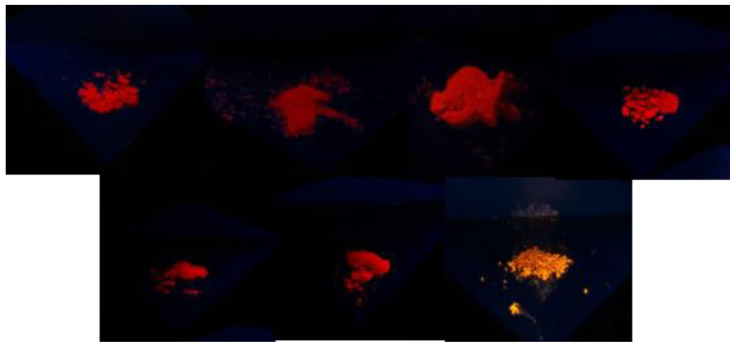
Some compounds (**3ac**, **3ai**, **3an**, **3as**, **3ay**, **4k**, **4m**) under 365 nm UV.

During the experiment, we found that the series of compounds showed an interesting photophysical phenomenon under 365 nm UV (Figure 2), so the photophysical properties of these compounds were characterized in the works (Figure 2, ESI Table S7 and Figures S2–S8). After comparing the fluorescence emission spectra of all products in ethyl acetate (EA) at a low concentration (3 × 10^−5^ M) (ESI Table S7, Figures S6 and S7), some interesting results were delivered. They emit between 565 and 662 nm in EA with moderate to good quantum yields (ranging from 0.10 to 0.83). The results indicated that the change of the functional groups on the indole-naphthoquinone skeletons had a greater impact on the relative quantum yield (ΦF) of compounds, while the change of the *N*-substituents of the maleimide had no significant impact on the photophysical properties of the compounds. Overall, these compounds had excellent relative fluorescence quantum yields, which provided them the potential for becoming fluorescent probes or chromogenic chemosensors.

**Figure 3:**
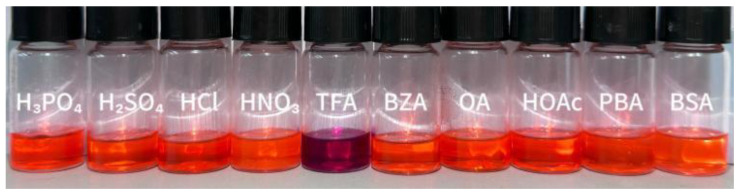
Colorimetric responses of **3aa** (5 × 10^−5^ M) in DCM after the addition of 1000 equiv of different acids (H_3_PO_4_, H_2_SO_4_, HCl, HNO_3_, TFA = trifluoroacetic acid, BZA = benzoic acid, OA = oxalic acid, HOAc, PBA = phenylboronic acid, BSA = benzenesulfonic acid).

**Figure 4:**
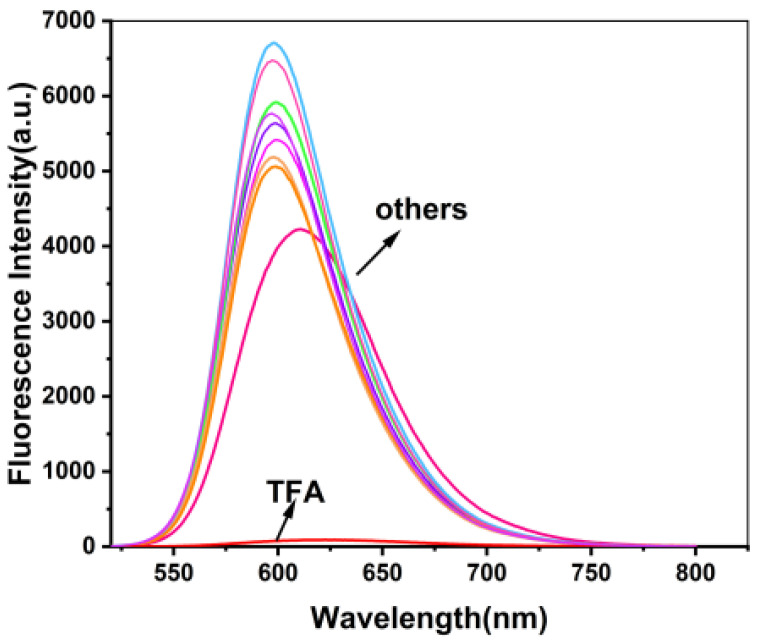
Changes in the fluorescence spectra of **3aa** (5 × 10^−5^ M) with acid.

To examine the selectivity, a fluorescence screening of various acids was carried out (Figure 3). Hence, **3aa** as the model compound (3.0 × 10^−5^ M) in dichloromethane (DCM) interacted with 1000 equiv of different acids such as H_3_PO_4_, H_2_SO_4_, HCl, HNO_3_, TFA, benzoic acid, oxalic acid, HAc, phenylboronic acid, and benzenesulfonic acid. While strong emission enhancement was observed for the majority of the acids tested, surprisingly, nearly no fluorescence was observed due to fluorescence quenching with the TFA added into the solution, confirming its suitability for the selective sensing of TFA (Figure 4). Furthermore, using this, one can detect TFA selectively as it changes to a distinctly different color (red to purple) (Figure 3). The quantitative analysis was carried out by fluorescence spectral titration of **3aa** with varying concentrations of TFA (0–1000 equiv) in DCM (Figure S8; Supplementary Materials). The colorimetric sensors are even better, as the signaling event can be detected by the naked eye itself.

## 3. Materials and Methods

Chemicals and analytical-grade solvents were purchased from commercial suppliers and used without further purification unless otherwise stated. All reagents were weighed and handled in air at room temperature. Analytical thin-layer chromatography was performed on glass plates of silica gel GF–254 with detection by UV light (254 and 365 nm). Column chromatography was carried out on silica gel (200–300 mesh). ^1^H NMR spectra were recorded at 400 MHz and ^13^C NMR spectra were recorded at 101 MHz by using Agilent 400 MHz NMR spectrometer (Swiss Bruker Company, Fällanden, Switzerland). Chemical shifts were calibrated using residual undeuterated solvent as an internal reference (^1^H NMR: CDCl_3_ 7.26 ppm, DMSO-*d_6_* 2.50 ppm, ^13^C NMR: CDCl_3_ 77.16 ppm, DMSO-*d_6_* 39.52 ppm). Data were reported as follows: chemical shift, multiplicity (s = singlet, br s = broad singlet, d = doublet, t = triplet, q = quartet, m = multiplet). Coupling constants (J) were reported in Hertz (Hz). Melting points were measured with a micro melting point apparatus. HRESIMS spectra were acquired using the waters G2-Xs qtof mass spectrometer (Waters, Milford, MA, USA). Single-crystal X-ray diffraction data were collected using a Bruker D8 Quest diffractometer (Swiss Bruker Company, Fällanden, Switzerland). (Mo Kα, λ = 0.71073 Å). Photophysical properties were evaluated on a Unic 4802 UV-Vis Double Beam spectrophotometer and a Horiba (Dual-fl) fluorescence spectrophotometer (Swiss Bruker Company, Fällanden, Switzerland). 

To a solution of bi-indolnaphthoquinone **1** (0.1 mmol) and Pd(OTf)_2_ (0.03 mmol), AgOAc (0.03 mmol), K_2_S_2_O_8_ (0.2 mmol), and HOAc (0.3 mmol) in CH_3_CN (2 mL) was added maleimide 2. The reaction mixture was stirred at 100 °C under a sealed tube for 1 h. After the completion of the reaction (monitored by TLC), the reaction was quenched with saturated salt water (6 mL), and the mixture was extracted with EtOAc (3 × 3 mL). The organic extracts were washed with brine, dried over Na_2_SO_4_, filtered, and the solvent was removed in vacuo. The crude product was purified by silica gel column chromatography to give **3** or **4**.

**1,5′,7′-trimethyl-5b′,8a′-dihydro-5′H,6′H-spiro[indoline-3,15′-[5a,8b]methanonaphtho[2,3-c]pyrrolo[3,4-a]carbazole]-2,6′,8′,9′,14′(7′H)-pentaone (3aa):** A pink solid (44 mg, 82% yield); mp 293–295 °C; purified over a column of silica gel (petroleum ether/EtOAc = 3:1); ^1^H NMR (400 MHz, CDCl_3_) δ 8.53 (d, *J* = 7.7 Hz, 1H), 8.26 (d, *J* = 7.7 Hz, 1H), 7.99 (d, *J* = 7.8 Hz, 1H), 7.77 (t, *J* = 7.5 Hz, 1H), 7.65 (t, *J* = 7.5 Hz, 1H), 7.45 (t, *J* = 7.8 Hz, 1H), 7.11 (t, *J* = 7.7 Hz, 1H), 6.96 (d, *J* = 7.5 Hz, 1H), 6.74 (d, *J* = 7.8 Hz, 1H), 6.67 (d, *J* = 9.0 Hz, 2H), 6.54 (t, *J* = 7.7 Hz, 1H), 4.89 (d, *J* = 8.0 Hz, 1H), 4.65 (d, *J* = 8.0 Hz, 1H), 3.34 (s, 3H), 3.01 (s, 3H), 2.71 (s, 3H). ^13^CNMR (101 MHz, CDCl_3_) δ 193.23, 178.41, 174.81, 173.66, 171.54, 162.62, 160.85, 143.80, 137.22, 136.75, 135.03, 134.79, 133.30, 129.86, 128.68, 127.52, 127.38, 124.41, 122.82, 121.85, 120.04, 119.77, 118.94, 108.88, 108.47, 85.69, 80.44, 69.23, 47.58, 41.17, 30.80, 26.60, 24.61. HRMS (ESI) *m*/*z* calcd for C_33_H_23_N_3_O_5_Na^+^(M + Na)^+^ 564.1533, found 564.1535.

**7′-ethyl-1,5′-dimethyl-5b′,8a′-dihydro-5′H,6′H-spiro[indoline-3,15′-[5a,8b]methanonaphtho[2,3-c]pyrrolo[3,4-a]carbazole]-2,6′,8′,9′,14′(7′H)-pentaone (3ab):** A pink solid (41 mg, 74% yield); mp 274–276 °C; purified over a column of silica gel (petroleum ether/EtOAc = 3:1); ^1^H NMR (400 MHz, CDCl_3_) δ 8.51 (d, *J* = 7.7 Hz, 1H), 8.27 (d, *J* = 7.4 Hz, 1H), 8.00 (d, *J* = 7.4 Hz, 1H), 7.77 (t, *J* = 7.1 Hz, 1H), 7.65 (t, *J* = 7.1 Hz, 1H), 7.46 (t, *J* = 7.3 Hz, 1H), 7.11 (t, *J* = 7.4 Hz, 1H), 6.95 (t, *J* = 7.5 Hz, 1H), 6.78–6.66 (m, 3H), 6.55 (t, *J* = 7.5 Hz, 1H), 4.85 (d, *J* = 7.9 Hz, 1H), 4.60 (d, *J* = 7.9 Hz, 1H), 3.34 (s, 3H), 3.30–3.21 (m, 2H), 3.02 (s, 3H), 0.86 (t, *J* = 7.1 Hz, 3H). ^13^C NMR (101 MHz CDCl_3_) δ 193.33, 178.23, 174.62, 173.50, 171.49, 162.77, 160.77, 143.82, 137.24, 136.71, 135.01, 134.91, 133.28, 129.84, 128.53, 127.46, 127.37, 124.46, 122.78, 121.94, 120.10, 119.76, 119.01, 108.94, 108.45, 86.01, 80.07, 69.44, 47.45, 41.01, 33.64, 30.86, 26.59, 12.75. HRMS (ESI) *m*/*z* calcd for C_34_H_25_N_3_O_5_Na^+^ (M + Na)^+^ 578.1683, found 578.1692.

**1,5′-dimethyl-7′-propyl-5b′,8a′-dihydro-5′H,6′H-spiro[indoline-3,15′-[5a,8b]methanonaphtho[2,3-c]pyrrolo[3,4-a]carbazole]-2,6′,8′,9′,14′(7′H)-pentaone (3ac):** A pink solid (40 mg, 70% yield); mp 289–291 °C; purified over a column of silica gel (petroleum ether/EtOAc = 3:1); ^1^H NMR (400 MHz, CDCl_3_) δ 8.52 (d, *J* = 7.7 Hz, 1H), 8.27 (d, *J* = 7.8 Hz, 1H), 7.99 (d, *J* = 7.7 Hz, 1H), 7.77 (t, *J* = 7.4 Hz, 1H), 7.64 (t, *J* = 7.5 Hz, 1H), 7.45 (t, *J* = 7.7 Hz, 1H), 7.11 (t, *J* = 7.7 Hz, 1H), 6.94 (t, *J* = 7.5 Hz, 1H), 6.77–6.65 (m, 3H), 6.55 (t, *J* = 7.7 Hz, 1H), 4.86 (d, *J* = 8.0 Hz, 1H), 4.61 (d, *J* = 8.0 Hz, 1H), 3.34 (s, 3H), 3.15 (t, *J* = 7.4 Hz, 2H), 3.01 (s, 3H), 1.26 (sextet, *J* = 7.0 Hz, 2H), 0.74 (t, *J* = 7.4 Hz, 3H). ^13^C NMR (101 MHz, CDCl_3_) δ 193.45, 178.22, 174.79, 173.72, 171.47, 162.71, 160.93, 143.81, 137.24, 136.71, 135.02, 134.88, 133.27, 129.84, 128.63, 127.48, 127.35, 124.45, 122.79, 121.94, 120.16, 119.74, 119.02, 108.91, 108.45, 85.87, 80.51, 69.31, 47.47, 41.00, 40.50, 30.84, 26.60, 20.81, 11.27. HRMS (ESI) *m*/*z* calcd for C_35_H_27_N_3_O_5_Na^+^ (M + Na)^+^ 592.1854, found 592.1848.

**7′-butyl-1,5′-dimethyl-5b′,8a′-dihydro-5′H,6′H-spiro[indoline-3,15′-[5a,8b]methanonaphtho[2,3-c]pyrrolo[3,4-a]carbazole]-2,6′,8′,9′,14′(7′H)-pentaone (3ad):** A pink solid (42 mg, 72% yield); mp 299–301 °C; purified over a column of silica gel (petroleum ether/EtOAc = 3:1); ^1^H NMR (400 MHz, CDCl_3_) δ 8.52 (d, *J* = 7.8 Hz, 1H), 8.27 (d, *J* = 7.8 Hz, 1H), 7.99 (d, *J* = 7.7 Hz, 1H), 7.76 (t, *J* = 7.5 Hz, 1H), 7.64 (t, *J* = 7.5 Hz, 1H), 7.45 (t, *J* = 7.8 Hz, 1H), 7.11 (t, *J* = 7.7 Hz, 1H), 6.94 (t, *J* = 7.5 Hz, 1H), 6.71 (dd, *J* = 7.6, 7.1 Hz, 3H), 6.55 (t, *J* = 7.6 Hz, 1H), 4.86 (d, *J* = 8.0 Hz, 1H), 4.61 (d, *J* = 8.0 Hz, 1H), 3.33 (s, 3H), 3.19 (t, *J* = 5.9 Hz, 2H), 3.01 (s, 3H), 1.23–1.05 (m, 4H), 0.84 (t, *J* = 7.1 Hz, 3H). ^13^C NMR (101 MHz, CDCl_3_) δ 188.66, 173.40, 170.04, 169.01, 166.71, 157.93, 156.09, 139.05, 132.53, 131.96, 130.27, 130.11, 128.50, 125.08, 123.90, 122.72, 122.59, 119.70, 118.05, 117.20, 115.59, 114.92, 114.28, 104.18, 103.70, 81.12, 76.17, 64.55, 42.73, 36.25, 33.93, 28.94, 26.11, 24.62, 21.85, 14.53. HRMS (ESI) *m*/*z* calcd for C_36_H_29_N_3_O_5_Na^+^ (M + Na)^+^ 606.2010, found 606.2005.

**7′-isobutyl-1,5′-dimethyl-5b′,8a′-dihydro-5′H,6′H-spiro[indoline-3,15′-[5a,8b]methanonaphtho[2,3-c]pyrrolo[3,4-a]carbazole]-2,6′,8′,9′,14′(7′H)-pentaone (3ae):** A pink solid (36 mg, 61% yield); mp 252–254 °C; purified over a column of silica gel (petroleum ether/EtOAc = 3:1); ^1^H NMR (400 MHz, CDCl_3_) δ 8.52 (d, *J* = 7.8 Hz, 1H), 8.26 (d, *J* = 7.8 Hz, 1H), 7.99 (d, *J* = 7.7 Hz, 1H), 7.76 (t, *J* = 7.5 Hz, 1H), 7.64 (t, *J* = 7.5 Hz, 1H), 7.44 (t, *J* = 7.7 Hz, 1H), 7.11 (t, *J* = 7.7 Hz, 1H), 6.93 (t, *J* = 7.5 Hz, 1H), 6.78–6.62 (m, 3H), 6.54 (t, *J* = 7.7 Hz, 1H), 4.89 (d, *J* = 8.2 Hz, 1H), 4.64 (d, *J* = 8.2 Hz, 1H), 3.34 (s, 3H), 3.03 (d, *J* = 11.1 Hz, 5H), 1.67 (dt, *J* = 20.6, 6.9 Hz, 1H), 0.70 (dd, *J* = 19.0, 6.7 Hz, 6H). ^13^C NMR (101 MHz, CDCl_3_) δ 193.22, 178.24, 175.05, 174.00, 171.42, 162.63, 161.00, 143.80, 137.26, 136.74, 135.03, 134.84, 133.28, 129.84, 128.85, 127.48, 127.32, 124.44, 122.81, 121.93, 120.46, 119.67, 119.04, 108.90, 108.44, 85.70, 80.87, 69.17, 47.42, 46.47, 41.00, 30.81, 27.01, 26.61, 20.08, 20.05. HRMS (ESI) *m*/*z* calcd for C_36_H_29_N_3_O_5_Na^+^ (M + Na)^+^ 606.2010, found 606.2005.

**7′-(tert-butyl)-1,5′-dimethyl-5b′,8a′-dihydro-5′H,6′H-spiro[indoline-3,15′-[5a,8b]methanonaphtho[2,3-c]pyrrolo[3,4-a]carbazole]-2,6′,8′,9′,14′(7′H)-pentaone (3af):** A pink solid (38 mg, 65% yield); mp 291–293 °C; purified over a column of silica gel (petroleum ether/EtOAc = 3:1); ^1^H NMR (400 MHz, CDCl_3_) δ 8.55 (d, *J* = 7.8 Hz, 1H), 8.27 (d, *J* = 7.9 Hz, 1H), 7.98 (d, *J* = 7.7 Hz, 1H), 7.76 (t, *J* = 7.5 Hz, 1H), 7.64 (t, *J* = 7.6 Hz, 1H), 7.46 (t, *J* = 7.8 Hz, 1H), 7.10 (t, *J* = 7.7 Hz, 1H), 6.95 (t, *J* = 7.5 Hz, 1H), 6.75–6.66 (m, 3H), 6.54 (t, *J* = 7.7 Hiiz, 1H), 4.70 (d, *J* = 8.3 Hz, 1H), 4.49 (d, *J* = 8.3 Hz, 1H), 3.33 (s, 3H), 3.00 (s, 3H), 1.28 (s, 9H). ^13^C NMR (101 MHz, CDCl_3_) δ 193.44, 178.44, 176.11, 175.00, 171.47, 162.85, 160.84, 143.84, 137.24, 136.61, 135.06, 134.91, 133.57, 133.30, 129.76, 128.48, 127.34, 127.24, 124.45, 122.71, 122.01, 120.30, 119.60, 119.16, 108.87, 108.40, 86.34, 80.18, 69.74, 58.90, 47.49, 40.57, 30.83, 28.64, 28.04, 26.58. HRMS (ESI) *m*/*z* calcd for C_36_H_29_N_3_O_5_Na^+^ (M + Na)^+^ 606.2012, found 606.2005.

**1,5′-dimethyl-7′-pentyl-5b′,8a′-dihydro-5′H,6′H-spiro[indoline-3,15′-[5a,8b]methanonaphtho[2,3-c]pyrrolo[3,4-a]carbazole]-2,6′,8′,9′,14′(7′H)-pentaone (3ag):** A pink solid (32 mg, 54% yield); mp 242–244 °C; purified over a column of silica gel (petroleum ether/EtOAc = 3:1); ^1^H NMR (400 MHz, CDCl_3_) δ 8.52 (d, *J* = 7.7 Hz, 1H), 8.27 (d, *J* = 7.8 Hz, 1H), 7.99 (d, *J* = 7.6 Hz, 1H), 7.77 (t, *J* = 7.4 Hz, 1H), 7.64 (t, *J* = 7.5 Hz, 1H), 7.45 (t, *J* = 7.6 Hz, 1H), 7.11 (t, *J* = 7.7 Hz, 1H), 6.94 (t, *J* = 7.5 Hz, 1H), 6.71 (dd, *J* = 25.7, 6.9 Hz, 3H), 6.55 (t, *J* = 7.7 Hz, 1H), 4.86 (d, *J* = 8.0 Hz, 1H), 4.61 (d, *J* = 8.0 Hz, 1H), 3.34 (s, 3H), 3.18 (td, *J* = 7.5, 3.2 Hz, 2H), 3.01 (s, 3H), 1.25 (d, *J* = 6.2 Hz, 4H), 1.08 (q, *J* = 7.5 Hz, 2H), 0.84 (t, *J* = 7.3 Hz, 3H). ^13^C NMR (101 MHz, CDCl_3_) δ 193.33, 178.14, 174.78, 173.73, 171.47, 162.69, 160.81, 143.81, 137.29, 136.68, 135.01, 134.88, 133.24, 129.82, 128.65, 127.47, 127.33, 124.45, 122.80, 121.96, 120.29, 119.69, 119.03, 108.92, 108.45, 85.88, 80.02, 69.33, 47.47, 41.00, 38.86, 30.86, 29.68, 28.71, 27.22, 26.60, 22.14, 13.73. HRMS (ESI) *m*/*z* calcd for C_37_H_31_N_3_O_5_Na^+^ (M + Na)^+^ 620.2168, found 620.2161.

**7′-hexyl-1,5′-dimethyl-5b′,8a′-dihydro-5′H,6′H-spiro[indoline-3,15′-[5a,8b]methanonaphtho[2,3-c]pyrrolo[3,4-a]carbazole]-2,6′,8′,9′,14′(7′H)-pentaone (3ah):** A pink solid (32 mg, 53% yield); mp 331–333 °C; purified over a column of silica gel (petroleum ether/EtOAc = 3:1); ^1^H NMR (400 MHz, CDCl_3_) δ 8.51 (d, *J* = 7.8 Hz, 1H), 8.27 (d, *J* = 7.8 Hz, 1H), 7.99 (d, *J* = 7.7 Hz, 1H), 7.77 (t, *J* = 7.4 Hz, 1H), 7.64 (t, *J* = 7.4 Hz, 1H), 7.45 (t, *J* = 7.6 Hz, 1H), 7.11 (t, *J* = 7.6 Hz, 1H), 6.94 (t, *J* = 7.5 Hz, 1H), 6.71 (dd, *J* = 26.3, 7.7 Hz, 3H), 6.55 (t, *J* = 7.6 Hz, 1H), 4.86 (d, *J* = 7.9 Hz, 1H), 4.60 (d, *J* = 7.9 Hz, 1H), 3.34 (s, 3H), 3.18 (s, 2H), 3.01 (s, 3H), 1.59 (s, 2H), 1.17 (dd, *J* = 40.6, 6.0 Hz, 6H), 0.86 (t, *J* = 6.7 Hz, 3H). ^13^C NMR (101 MHz, CDCl_3_) δ 193.21, 178.14, 174.77, 173.46, 171.47, 162.59, 160.61, 143.81, 137.28, 136.69, 135.01, 134.77, 133.24, 129.82, 128.64, 127.48, 127.34, 124.45, 122.80, 121.94, 120.28, 119.69, 119.03, 108.92, 108.45, 85.83, 80.43, 69.22, 47.48, 40.99, 38.87, 31.22, 30.86, 27.48, 26.60, 26.33, 22.31, 14.02. HRMS (ESI) *m*/*z* calcd for C_38_H_33_N_3_O_5_Na^+^ (M + Na)^+^ 634.2322, found 634.2318.

**7′-cyclopropyl-1,5′-dimethyl-5b′,8a′-dihydro-5′H,6′H-spiro[indoline-3,15′-[5a,8b]methanonaphtho[2,3-c]pyrrolo[3,4-a]carbazole]-2,6′,8′,9′,14′(7′H)-pentaone (3ai):** A pink solid (34 mg, 60% yield); mp 273–275 °C; purified over a column of silica gel (petroleum ether/EtOAc = 3:1); ^1^H NMR (400 MHz, CDCl_3_) δ 8.51 (d, *J* = 7.7 Hz, 1H), 8.28 (d, *J* = 7.7 Hz, 1H), 7.99 (d, *J* = 7.7 Hz, 1H), 7.77 (t, *J* = 7.5 Hz, 1H), 7.65 (t, *J* = 7.5 Hz, 1H), 7.46 (t, *J* = 7.7 Hz, 1H), 7.11 (t, *J* = 7.7 Hz, 1H), 6.96 (t, *J* = 7.4 Hz, 1H), 6.77–6.65 (m, 3H), 6.54 (t, *J* = 7.6 Hz, 1H), 4.81 (d, *J* = 8.0 Hz, 1H), 4.56 (d, *J* = 8.0 Hz, 1H), 3.33 (s, 3H), 3.00 (s, 3H), 2.24 (dd, *J* = 11.4, 7.7 Hz, 1H), 0.81–0.65 (m, 2H), 0.55 (dd, *J* = 10.2, 5.3 Hz, 1H), 0.42–0.33 (m, 1H). ^13^C NMR (101 MHz, CDCl_3_) δ 193.10, 178.42, 175.30, 174.12, 171.27, 162.65, 160.58, 143.80, 137.10, 136.84, 135.05, 134.90, 133.35, 129.85, 128.32, 127.48, 127.38, 124.42, 122.76, 121.90, 119.99, 119.85, 118.77, 109.04, 108.46, 86.12, 79.72, 69.26, 47.09, 40.54, 30.85, 26.60, 21.50, 5.14. HRMS (ESI) *m*/*z* calcd for C_35_H_25_N_3_O_5_Na^+^ (M + Na)^+^ 590.1694, found 590.1692.

**7′-cyclopentyl-1,5′-dimethyl-5b′,8a′-dihydro-5′H,6′H-spiro[indoline-3,15′-[5a,8b]methanonaphtho[2,3-c]pyrrolo[3,4-a]carbazole]-2,6′,8′,9′,14′(7′H)-pentaone (3aj):** A pink solid (37 mg, 65% yield); mp 205–207 °C; purified over a column of silica gel (petroleum ether/EtOAc = 3:1); ^1^H NMR (400 MHz, CDCl_3_) δ 8.52 (d, *J* = 7.8 Hz, 1H), 8.27 (d, *J* = 7.8 Hz, 1H), 7.99 (d, *J* = 7.7 Hz, 1H), 7.77 (t, *J* = 7.4 Hz, 1H), 7.65 (t, *J* = 7.5 Hz, 1H), 7.46 (t, *J* = 7.7 Hz, 1H), 7.11 (t, *J* = 7.7 Hz, 1H), 6.95 (t, *J* = 7.5 Hz, 1H), 6.76–6.66 (m, 3H), 6.54 (t, *J* = 7.7 Hz, 1H), 4.80 (d, *J* = 8.0 Hz, 1H), 4.56 (d, *J* = 8.0 Hz, 1H), 4.15–4.08 (m, 1H), 3.33 (s, 3H), 3.01 (s, 3H), 1.79–1.57 (m, 8H). ^13^C NMR (101 MHz, CDCl_3_) δ 193.08, 178.08, 175.17, 173.96, 171.51, 162.78, 160.56, 143.81, 137.23, 136.67, 134.98, 133.27, 129.80, 128.52, 127.46, 127.32, 124.44, 122.75, 121.98, 120.19, 119.72, 119.11, 108.90, 108.43, 85.52, 79.69, 69.42, 51.83, 47.25, 40.61, 30.86, 29.68, 28.07, 26.59, 24.67. HRMS (ESI) *m*/*z* calcd for C_37_H_29_N_3_O_5_Na^+^ (M + Na)^+^ 618.2010, found 618.2005.

**7′-cyclohexyl-1,5′-dimethyl-5b′,8a′-dihydro-5′H,6′H-spiro[indoline-3,15′-[5a,8b]methanonaphtho[2,3-c]pyrrolo[3,4-a]carbazole]-2,6′,8′,9′,14′(7′H)-pentaone (3ak):** A pink solid (35 mg, 57% yield); mp 292–294 °C; purified over a column of silica gel (petroleum ether/EtOAc = 3:1); ^1^H NMR (400 MHz, CDCl_3_) δ 8.52 (d, *J* = 7.8 Hz, 1H), 8.27 (d, *J* = 7.8 Hz, 1H), 7.99 (d, *J* = 7.8 Hz, 1H), 7.76 (t, *J* = 7.6 Hz, 1H), 7.64 (t, *J* = 7.5 Hz, 1H), 7.45 (t, *J* = 7.8 Hz, 1H), 7.10 (t, *J* = 7.7 Hz, 1H), 6.95 (t, *J* = 7.5 Hz, 1H), 6.76–6.65 (m, 3H), 6.54 (t, *J* = 7.7 Hz, 1H), 4.79 (d, *J* = 8.0 Hz, 1H), 4.56 (d, *J* = 8.0 Hz, 1H), 3.63 (dd, *J* = 16.3, 8.0 Hz, 1H), 3.33 (s, 3H), 3.00 (s, 3H), 1.88–1.65 (m, 4H), 1.50 (d, *J* = 12.5 Hz, 1H), 1.36 (d, *J* = 12.1 Hz, 2H), 1.16–0.98 (m, 3H). ^13^C NMR (101 MHz, CDCl_3_) δ 193.34, 178.23, 174.98, 173.92, 171.47, 162.77, 160.82, 143.82, 137.20, 136.66, 134.98, 133.24, 129.80, 128.52, 127.51, 127.30, 124.43, 122.74, 121.97, 120.25, 119.74, 119.12, 112.31, 108.87, 108.42, 86.00, 80.27, 69.43, 51.73, 47.22, 40.55, 30.86, 28.27, 28.19, 26.59, 25.69, 24.79. HRMS (ESI) *m*/*z* calcd for C_38_H_31_N_3_O_5_Na^+^ (M + Na)^+^ 632.2167, found 632.2161.

**1,5′-dimethyl-5b′,8a′-dihydro-5′H,6′H-spiro[indoline-3,15′-[5a,8b]methanonaphtho[2,3-c]pyrrolo[3,4-a]carbazole]-2,6′,8′,9′,14′(7′H)-pentaone (3al):** A pink solid (33 mg, 63% yield); mp 271–273 °C; purified over a column of silica gel (petroleum ether/EtOAc = 3:1); ^1^H NMR (400 MHz, CDCl_3_) δ 8.58 (d, *J* = 7.7 Hz, 1H), 8.27 (d, *J* = 7.6 Hz, 1H), 7.98 (d, *J* = 7.4 Hz, 1H), 7.76 (t, *J* = 7.4 Hz, 1H), 7.63 (dd, *J* = 6.7, 8.0 Hz, 2H), 7.45 (t, *J* = 7.7 Hz, 1H), 7.11 (t, *J* = 7.7 Hz, 1H), 6.95 (t, *J* = 7.5 Hz, 1H), 6.76–6.65 (m, 3H), 6.56 (t, *J* = 7.6 Hz, 1H), 4.93 (d, *J* = 8.0 Hz, 1H), 4.69 (d, *J* = 8.1 Hz, 1H), 3.33 (s, 3H), 2.99 (s, 3H). ^13^C NMR (101 MHz, CDCl_3_) δ 188.87, 178.25, 174.21, 173.12, 171.47, 162.41, 161.13, 137.16, 136.78, 135.06, 134.67, 133.35, 129.91, 128.90, 127.56, 127.35, 124.42, 122.88, 121.69, 120.23, 119.78, 118.87, 109.98, 108.89, 108.50, 85.40, 80.49, 68.87, 48.67, 42.40, 30.73, 26.61. HRMS (ESI) *m*/*z* calcd for C_32_H_21_N_3_O_5_Na^+^ (M + Na)^+^ 550.1384, found 550.1379.

**7′-benzyl-1,5′-dimethyl-5b′,8a′-dihydro-5′H,6′H-spiro[indoline-3,15′-[5a,8b]methanonaphtho[2,3-c]pyrrolo[3,4-a]carbazole]-2,6′,8′,9′,14′(7′H)-pentaone (3am)**: A pink solid (50 mg, 81% yield); mp 242–244 °C; purified over a column of silica gel (petroleum ether/EtOAc = 3:1); ^1^H NMR (400 MHz, CDCl_3_) δ 8.26–8.19 (m, 2H), 7.98 (d, *J* = 7.7 Hz, 1H), 7.76 (t, *J* = 7.4 Hz, 1H), 7.64 (t, *J* = 7.4 Hz, 1H), 7.41 (t, *J* = 7.6 Hz, 1H), 7.31–7.24 (m, 3H), 7.11–7.02 (m, 3H), 6.88 (t, *J* = 7.5 Hz, 1H), 6.74–6.51 (m, 4H), 4.89 (d, *J* = 8.1 Hz, 1H), 4.65 (d, *J* = 8.1 Hz, 1H), 4.38 (d, *J* = 14.1 Hz, 1H), 4.33 (d, *J* = 14.2 Hz, 1H), 3.32 (s, 3H), 2.99 (s, 3H). ^13^C NMR (101 MHz, CDCl_3_) δ 193.27, 177.75, 174.46, 173.35, 171.41, 162.46, 160.75, 143.79, 137.38, 136.62, 135.02, 134.94, 134.79, 133.21, 132.72, 129.82, 128.98, 128.67, 128.47, 127.68, 127.42, 127.27, 124.42, 122.81, 121.90, 120.19, 119.47, 118.84, 112.41, 108.71, 108.45, 85.79, 80.92, 69.18, 47.54, 42.46, 41.08, 30.84, 26.59. HRMS (ESI) *m*/*z* calcd for C_39_H_27_N_3_O_5_Na^+^ (M + Na)^+^ 640.1848, found 640.1848.

**7′-(2-chlorobenzyl)-1,5′-dimethyl-5b′,8a′-dihydro-5′H,6′H-spiro[indline-3,15′-[5a,8b]methanonaphtho[2,3-c]pyrrolo[3,4-a]carbazole]-2,6′,8′,9′,14′(7′H)-pentaone (3an)**: A pink solid (47 mg, 72% yield); mp 244–246 °C; purified over a column of silica gel (petroleum ether/EtOAc = 3:1); ^1^H NMR (400 MHz, CDCl_3_) δ 8.53 (d, *J* = 7.8 Hz, 1H), 8.23 (d, *J* = 7.8 Hz, 1H), 7.98 (d, *J* = 7.7 Hz, 1H), 7.74 (t, *J* = 7.5 Hz, 1H), 7.63 (t, *J* = 7.6 Hz, 1H), 7.42 (t, *J* = 7.8 Hz, 1H), 7.33 (t, *J* = 7.5 Hz, 1H), 7.30–7.16 (m, 2H), 7.12 (t, *J* = 7.7 Hz, 1H), 6.91 (t, *J* = 7.5 Hz, 2H), 6.75 (d, *J* = 7.8 Hz, 1H), 6.66 (t, *J* = 9.2 Hz, 2H), 6.56 (t, *J* = 7.7 Hz, 1H), 4.99 (d, *J* = 8.3 Hz, 1H), 4.74 (d, *J* = 8.3 Hz, 1H), 4.54 (s, 2H), 3.34 (s, 3H), 3.02 (s, 3H). ^13^C NMR (101 MHz, CDCl_3_) δ 193.14, 178.40, 174.39, 172.99, 171.35, 162.68, 161.57, 143.50, 137.18, 136.87, 135.05, 134.72, 133.31, 132.57, 131.71, 129.91, 129.33, 128.78, 128.61, 128.03, 127.56, 127.37, 124.42, 122.88, 121.81, 120.53, 119.68, 118.91, 108.95, 108.51, 84.95, 80.88, 68.82, 47.62, 41.23, 40.38, 30.79, 26.63. HRMS (ESI) *m*/*z* calcd for C_39_H_26_N_3_O_5_NaCl^+^ (M + Na)^+^ 674.1453, found 674.1459.

**7′-(3-chlorobenzyl)-1,5′-dimethyl-5b′,8a′-dihydro-5′H,6′H-spiro[indoline-3,15′-[5a,8b]methanonaphtho[2,3-c]pyrrolo[3,4-a]carbazole]-2,6′,8′,9′,14′(7′H)-pentaone (3ao):** A pink solid (48 mg, 73% yield); mp 349–351 °C; purified over a column of silica gel (petroleum ether/EtOAc = 3:1); ^1^H NMR (400 MHz, CDCl_3_) δ 8.22 (d, *J* = 7.7 Hz, 1H), 8.15 (d, *J* = 7.8 Hz, 1H), 7.97 (d, *J* = 7.6 Hz, 1H), 7.77 (dd, *J* = 10.7, 4.4 Hz, 1H), 7.65 (t, *J* = 6.9 Hz, 1H), 7.43 (t, *J* = 7.8 Hz, 1H), 7.33 (d, *J* = 8.0 Hz, 1H), 7.26–7.21 (m, 1H), 7.13–7.04 (m, 2H), 6.93–6.87 (m, 2H), 6.73 (d, *J* = 7.7 Hz, 1H), 6.65–6.51 (m, 3H), 4.89 (d, *J* = 8.1 Hz, 1H), 4.64 (d, *J* = 8.0 Hz, 1H), 4.32 (d, *J* = 13.8 Hz, 1H), 4.30 (d, *J* = 13.6 Hz, 1H), 3.33 (s, 3H), 2.99 (s, 3H). ^13^C NMR (101 MHz, CDCl_3_) δ 193.32, 177.60, 174.32, 173.20, 171.42, 162.45, 160.69, 143.78, 137.34, 136.64, 135.06, 134.78, 134.33, 133.23, 130.10, 129.83, 129.03, 128.92, 128.16, 127.46, 127.24, 127.06, 124.41, 122.83, 121.81, 119.83, 119.64, 118.75, 108.69, 108.46, 85.80, 80.37, 69.35, 47.42, 41.83, 41.10, 30.84, 26.60. HRMS (ESI) *m*/*z* calcd for C_39_H_26_N_3_O_5_NaCl^+^ (M + Na)^+^ 674.1461, found 674.1459.

**7′-(3-bromobenzyl)-1,5′-dimethyl-5b′,8a′-dihydro-5′H,6′H-spiro[indoline-3,15′-[5a,8b]methanonaphtho[2,3-c]pyrrolo[3,4-a]carbazole]-2,6′,8′,9′,14′(7′H)-pentaone (3ap)**: A pink solid (52 mg, 75% yield); mp 234–236 °C; purified over a column of silica gel (petroleum ether/EtOAc = 3:1); ^1^H NMR (400 MHz, CDCl_3_) δ 8.00 (d, *J* = 7.8 Hz, 1H), 7.91 (d, *J* = 7.8 Hz, 1H), 7.74 (d, *J* = 7.7 Hz, 1H), 7.54 (t, *J* = 7.5 Hz, 1H), 7.42 (t, *J* = 7.5 Hz, 1H), 7.26–7.17 (m, 2H), 7.03–6.92 (m, 3H), 6.86 (t, *J* = 7.5 Hz, 1H), 6.74–6.65 (m, 2H), 6.49 (d, *J* = 7.8 Hz, 1H), 6.38 (dd, *J* = 7.3, 8.0 Hz, 2H), 6.28 (t, *J* = 7.6 Hz, 1H), 4.65 (d, *J* = 8.0 Hz, 1H), 4.40 (d, *J* = 8.0 Hz, 1H), 4.08 (d, *J* = 14.0 Hz, 1H), 4.03 (d, *J* = 14.1 Hz, 1H), 3.09 (s, 3H), 2.76 (s, 3H). ^13^C NMR (101 MHz, CDCl_3_) δ 193.19, 177.58, 174.42, 173.23, 171.63, 162.26, 160.42, 143.91, 137.32, 136.96, 136.64, 135.06, 134.78, 133.24, 131.91, 131.09, 130.42, 129.83, 128.91, 127.56, 127.51, 127.24, 124.42, 122.82, 122.47, 121.81, 119.88, 119.69, 118.74, 108.69, 108.46, 85.76, 80.52, 69.29, 47.43, 41.76, 41.10, 30.85, 26.60. HRMS (ESI) *m*/*z* calcd for C_39_H_26_N_3_O_5_NaBr^+^ (M + Na)^+^ 718.0958, found 718.0954.

**1,5′-dimethyl-7′-(4-methylbenzyl)-5b′,8a′-dihydro-5′H,6′H-spiro[indoline-3,15′-[5a,8b]methanonaphtho[2,3-c]pyrrolo[3,4-a]carbazole]-2,6′,8′,9′,14′(7′H)-pentaone (3aq)**: A pink solid (43 mg, 68% yield); mp 302–304 °C; purified over a column of silica gel (petroleum ether/EtOAc = 3:1); ^1^H NMR (400 MHz, CDCl_3_) δ 8.24 (dd, *J* = 7.1, 3.9 Hz, 2H), 7.98 (d, *J* = 7.7 Hz, 1H), 7.77 (t, *J* = 7.4 Hz, 1H), 7.64 (t, *J* = 7.2 Hz, 1H), 7.41 (t, *J* = 7.5 Hz, 1H), 7.08 (dd, *J* = 6.3, 7.4 Hz, 3H), 6.95–6.84 (m, 3H), 6.73 (d, *J* = 7.8 Hz, 1H), 6.65–6.58 (m, 2H), 6.52 (t, *J* = 7.6 Hz, 1H), 4.87 (d, *J* = 8.1 Hz, 1H), 4.62 (d, *J* = 8.1 Hz, 1H), 4.37–4.26 (m, 2H), 3.32 (s, 3H), 2.99 (s, 3H), 2.37 (s, 3H). ^13^C NMR (101 MHz, CDCl_3_) δ 193.35, 177.98, 174.46, 173.35, 171.41, 162.45, 160.77, 143.79, 137.39, 137.33, 136.61, 135.01, 134.81, 133.21, 132.01, 129.80, 129.31, 128.94, 128.45, 127.42, 127.28, 124.42, 122.79, 121.92, 120.19, 119.36, 118.84, 108.70, 108.43, 85.79, 80.52, 69.17, 47.56, 42.19, 41.03, 30.83, 26.59, 21.18. HRMS (ESI) *m*/*z* calcd for C_40_H_29_N_3_O_5_Na^+^ (M + Na)^+^ 654.2008, found 654.2005.

**7′-(4-methoxybenzyl)-1,5′-dimethyl-5b′,8a′-dihydro-5′H,6′H-spiro[indoline-3,15′-[5a,8b]methanonaphtho[2,3-c]pyrrolo[3,4-a]carbazole]-2,6′,8′,9′,14′(7′H)-pentaone (3ar)**: A pink solid (40 mg, 62% yield); mp 344–346 °C; purified over a column of silica gel (petroleum ether/EtOAc = 3:1); ^1^H NMR (400 MHz, CDCl_3_) δ 8.24 (t, *J* = 7.2 Hz, 2H), 7.98 (d, *J* = 7.7 Hz, 1H), 7.77 (t, *J* = 7.5 Hz, 1H), 7.64 (d, *J* = 7.5 Hz, 1H), 7.41 (t, *J* = 7.8 Hz, 1H), 7.09 (t, *J* = 7.7 Hz, 1H), 6.98 (d, *J* = 8.4 Hz, 2H), 6.87 (d, *J* = 7.5 Hz, 1H), 6.75 (dd, *J* = 7.8, 8.1 Hz, 3H), 6.65–6.59 (m, 2H), 6.51 (t, *J* = 7.7 Hz, 1H), 4.86 (d, *J* = 8.1 Hz, 1H), 4.62 (d, *J* = 8.1 Hz, 1H), 4.34–4.23 (m, 2H), 3.86 (s, 3H), 3.32 (s, 3H), 2.99 (s, 3H). ^13^C NMR (101 MHz, CDCl_3_) δ 193.32, 177.58, 174.28, 173.36, 171.28, 162.24, 160.53, 159.14, 143.79, 137.32, 136.60, 135.00, 134.73, 133.20, 129.98, 129.80, 128.95, 127.40, 127.36, 127.27, 124.42, 122.78, 121.78, 120.02, 119.36, 118.79, 114.07, 108.69, 108.43, 85.80, 80.63, 68.60, 55.36, 47.50, 41.88, 41.02, 30.82, 26.58. HRMS (ESI) *m*/*z* calcd for C_40_H_29_N_3_O_6_K^+^ (M + K)^+^ 686.1696, found 686.1693.

**7′-(4-fluorobenzyl)-1,5′-dimethyl-5b′,8a′-dihydro-5′H,6′H-spiro[indline-3,15′-[5a,8b]methanonaphtho[2,3-c]pyrrolo[3,4-a]carbazole]-2,6′,8′,9′,14′(7′H)-pentaone (3as)**: A pink solid (36 mg, 57% yield); mp 335–337 °C; purified over a column of silica gel (petroleum ether/EtOAc = 3:1); ^1^H NMR (400 MHz, CDCl_3_) δ 8.20 (dd, *J* = 29.8, 7.7 Hz, 2H), 7.98 (d, *J* = 7.6 Hz, 1H), 7.78 (t, *J* = 7.4 Hz, 1H), 7.65 (t, *J* = 7.4 Hz, 1H), 7.41 (t, *J* = 7.6 Hz, 1H), 7.26 (d, *J* = 3.9 Hz, 2H), 7.09 (t, *J* = 7.6 Hz, 1H), 7.00–6.87 (m, 3H), 6.73 (d, *J* = 7.7 Hz, 1H), 6.66–6.50 (m, 3H), 4.89 (d, *J* = 8.0 Hz, 1H), 4.64 (d, *J* = 8.0 Hz, 1H), 4.31 (dd, *J* = 49.3, 14.1 Hz, 2H), 3.32 (s, 3H), 2.98 (s, 3H). ^13^C NMR (101 MHz, CDCl_3_) δ 192.87, 177.51, 174.27, 173.26, 171.46, 162.29 (d, *J_F_* = 156 Hz), 160.73, 143.70, 137.33, 136.71, 135.08, 134.77, 133.68, 133.25, 130.04, 129.85, 128.90, 128.86, 127.42 (d, *J_F_* = 12 Hz), 127.30, 124.41, 122.83, 121.81, 120.03, 119.45, 118.62, 112.35, 108.70 (d, *J_F_* = 24 Hz), 108.46, 85.73, 80.47, 69.16, 47.51, 41.80, 41.02, 30.81, 26.60. ^19^F NMR (376 MHz, CDCl_3_) δ 85.04. HRMS (ESI) *m*/*z* calcd for C_39_H_26_N_3_O_5_NaF^+^ (M + Na)^+^ 658.1758.

**7′-(4-chlorobenzyl)-1,5′-dimethyl-5b′,8a′-dihydro-5′H,6′H-spiro[indline-3,15′-[5a,8b]methanonaphtho[2,3-c]pyrrolo[3,4-a]carbazole]-2,6′,8′,9′,14′(7′H)-pentaone (3at)**: A pink solid (41 mg, 63% yield); mp 349–351 °C; purified over a column of silica gel (petroleum ether/EtOAc = 3:1); ^1^H NMR (400 MHz, CDCl_3_) δ 8.20 (t, *J* = 8.9 Hz, 2H), 7.98 (d, *J* = 7.7 Hz, 1H), 7.77 (t, *J* = 7.3 Hz, 1H), 7.65 (t, *J* = 7.3 Hz, 1H), 7.42 (t, *J* = 7.4 Hz, 1H), 7.13–6.87 (m, 6H), 6.73 (d, *J* = 7.7 Hz, 1H), 6.65–6.50 (m, 3H), 4.88 (d, *J* = 8.1 Hz, 1H), 4.36 (d, *J* = 14.1 Hz, 1H), 4.27 (d, *J* = 14.1 Hz, 1H), 3.32 (s, 3H), 2.99 (s, 3H). ^13^C NMR (101 MHz, CDCl_3_) δ 193.05, 177.65, 174.34, 172.98, 171.34, 163.48, 162.41, 160.35, 143.76, 137.30, 136.68, 135.06, 134.74, 133.25, 130.72, 130.70, 130.49, 130.41, 129.84, 128.84, 127.39, 127.30, 124.41, 122.84, 121.74, 120.09, 119.43, 118.59, 115.64, 115.43, 108.72, 108.46, 85.61, 80.52, 69.00, 47.48, 41.72, 41.04, 30.82, 26.60. HRMS (ESI) *m*/*z* calcd for C_39_H_26_N_3_O_5_NaCl^+^ (M + Na)^+^ 674.1463, found 674.1459.

**7′-(4-iodobenzyl)-1,5′-dimethyl-5b′,8a′-dihydro-5′H,6′H-spiro[indline-3,15′-[5a,8b]methanonaphtho[2,3-c]pyrrolo[3,4-a]carbazole]-2,6′,8′,9′,14′(7′H)-pentaone (3au):** A pink solid (50 mg, 67% yield); mp 246–248 °C; purified over a column of silica gel (petroleum ether/EtOAc = 3:1); ^1^H NMR (400 MHz, CDCl_3_) δ 8.24 (d, *J* = 7.8 Hz, 1H), 8.13 (d, *J* = 7.7 Hz, 1H), 7.98 (d, *J* = 7.8 Hz, 1H), 7.78 (t, *J* = 7.5 Hz, 1H), 7.64 (dd, *J* = 6.8, 7.7 Hz, 3H), 7.41 (t, *J* = 7.7 Hz, 1H), 7.09 (t, *J* = 7.6 Hz, 1H), 6.93 (t, *J* = 7.5 Hz, 1H), 6.75 (dd, *J* = 6.9, 7.8 Hz, 3H), 6.56 (dt, *J* = 15.3, 8.3 Hz, 3H), 4.88 (d, *J* = 8.1 Hz, 1H), 4.63 (d, *J* = 8.1 Hz, 1H), 4.36 (d, *J* = 14.0 Hz, 1H), 4.20 (d, *J* = 14.1 Hz, 1H), 3.32 (s, 3H), 2.98 (s, 3H). ^13^C NMR (101 MHz, CDCl_3_) δ 193.45, 177.52, 174.40, 173.23, 171.22, 162.36, 160.61, 143.75, 137.86, 137.28, 136.72, 135.09, 134.76, 134.41, 133.25, 130.63, 129.85, 128.91, 127.44, 127.31, 124.42, 122.83, 121.75, 120.06, 119.52, 118.63, 108.68, 108.47, 93.65, 85.81, 80.43, 68.99, 47.52, 41.98, 41.00, 30.82, 26.60. HRMS (ESI) *m*/*z* calcd for C_39_H_26_N_3_O_5_NaI^+^ (M + Na)^+^ 766.0822, found 766.0815.

**1,5′-dimethyl-7′-phenethyl-5b′,8a′-dihydro-5′H,6′H-spiro[indoline-3,15′-[5a,8b]methanonaphtho[2,3-c]pyrrolo[3,4-a]carbazole]-2,6′,8′,9′,14′(7′H)-pentaone (3av)**: A pink solid (51 mg, 80% yield); mp 171–173 °C; purified over a column of silica gel (petroleum ether/EtOAc = 3:1); ^1^H NMR (400 MHz, CDCl_3_) δ 8.52 (s, 1H), 8.29 (d, *J* = 7.7 Hz, 1H), 8.01 (d, *J* = 7.6 Hz, 1H), 7.78 (t, *J* = 7.5 Hz, 1H), 7.66 (t, *J* = 7.5 Hz, 1H), 7.47 (t, *J* = 7.8 Hz, 1H), 7.26–7.07 (m, 6H), 6.95 (t, *J* = 7.5 Hz, 1H), 6.72 (dd, *J* = 8.3, 7.9 Hz, 3H), 6.55 (t, *J* = 7.7 Hz, 1H), 4.88 (d, *J* = 8.0 Hz, 1H), 4.63 (d, *J* = 7.9 Hz, 1H), 3.46–3.39 (m, 2H), 3.35 (s, 3H), 3.03 (s, 3H), 2.52 (ddd, *J* = 31.9, 10.3, 6.0 Hz, 2H). ^13^C NMR (101 MHz, CDCl_3_) δ 193.22, 178.21, 174.58, 173.51, 171.47, 162.75, 160.76, 143.81, 137.73, 137.24, 136.80, 135.08, 134.89, 133.32, 129.88, 128.69, 128.56, 128.44, 127.55, 127.38, 126.53, 124.45, 122.82, 121.90, 120.24, 119.85, 119.00, 108.98, 108.49, 85.96, 80.49, 69.37, 47.52, 41.02, 39.87, 33.49, 30.88, 26.62. HRMS (ESI) *m*/*z* calcd for C_40_H_29_N_3_O_5_Na^+^ (M + Na)^+^ 654.2007, found 654.2005.

**7′-(2-ethoxyethyl)-1,5′-dimethyl-5b′,8a′-dihydro-5′H,6′H-spiro[indline-3,15′-[5a,8b]methanonaphtho[2,3-c]pyrrolo[3,4-a]carbazole]-2,6′,8′,9′,14′(7′H)-pentaone (3aw):** A pink solid (46 mg, 76% yield); mp 254–256 °C; purified over a column of silica gel (petroleum ether/EtOAc = 3:1); ^1^H NMR (400 MHz, CDCl_3_) δ 8.54 (d, *J* = 7.7 Hz, 1H), 8.26 (d, *J* = 7.8 Hz, 1H), 7.98 (d, *J* = 7.7 Hz, 1H), 7.76 (t, *J* = 7.5 Hz, 1H), 7.64 (t, *J* = 7.5 Hz, 1H), 7.45 (t, *J* = 7.7 Hz, 1H), 7.11 (t, *J* = 7.6 Hz, 1H), 6.94 (t, *J* = 7.5 Hz, 1H), 6.70 (dd, *J* = 7.2, 7.8 Hz, 3H), 6.54 (t, *J* = 7.7 Hz, 1H), 4.88 (d, *J* = 8.0 Hz, 1H), 4.64 (d, *J* = 8.0 Hz, 1H), 3.45–3.35 (m, 4H), 3.33 (s, 3H), 3.20 (dd, *J* = 15.5, 6.6 Hz, 2H), 3.01 (s, 3H), 1.08 (t, *J* = 7.0 Hz, 3H). ^13^C NMR (101 MHz, CDCl_3_) δ 193.07, 178.16, 174.58, 173.51, 171.43, 162.67, 160.82, 143.81, 137.24, 136.71, 135.01, 134.79, 133.28, 129.86, 128.60, 127.48, 127.37, 124.43, 122.81, 121.90, 120.12, 119.72, 118.99, 109.92, 108.94, 108.47, 85.78, 80.57, 69.26, 66.09, 66.02, 47.49, 41.06, 37.89, 30.84, 26.61, 14.92. HRMS (ESI) *m*/*z* calcd for C_36_H_29_N_3_O_6_Na^+^ (M + Na)^+^ 622.1962, found 622.1954.

**1,5′-dimethyl-7′-(thiophen-2-ylmethyl)-5b′,8a′-dihydro-5′H,6′H-spiro[indoline-3,15′-[5a,8b]methanonaphtho[2,3-c]pyrrolo[3,4-a]carbazole]-2,6′,8′,9′,14′(7′H)-pentaone (3ax)**: A pink solid (48 mg, 77% yield); mp 277–279 °C; purified over a column of silica gel (petroleum ether/EtOAc = 3:1); ^1^H NMR (400 MHz, CDCl_3_) δ 8.23 (d, *J* = 7.7 Hz, 2H), 7.98 (d, *J* = 7.7 Hz, 1H), 7.78 (t, *J* = 7.3 Hz, 1H), 7.65 (t, *J* = 7.3 Hz, 1H), 7.44 (d, *J* = 7.5 Hz, 1H), 7.22 (d, *J* = 4.8 Hz, 1H), 7.09 (t, *J* = 7.6 Hz, 1H), 6.91 (dd, *J* = 9.3, 6.0 Hz, 2H), 6.75 (dd, *J* = 17.7, 5.1 Hz, 2H), 6.63 (dd, *J* = 15.8, 8.0 Hz, 2H), 6.53 (t, *J* = 7.6 Hz, 1H), 4.88 (d, *J* = 8.1 Hz, 1H), 4.63 (d, *J* = 8.1 Hz, 1H), 4.51 (s, 2H), 3.32 (s, 3H), 3.00 (s, 3H). ^13^C NMR (101 MHz, CDCl_3_) δ 193.30, 177.74, 173.96, 172.91, 171.40, 162.48, 160.61, 143.78, 137.42, 136.62, 136.09, 135.04, 134.79, 133.20, 129.82, 129.06, 127.98, 127.44, 127.29, 126.84, 125.88, 124.43, 122.82, 121.87, 120.07, 119.43, 118.86, 108.71, 108.44, 85.80, 80.45, 69.20, 47.47, 41.05, 36.14, 30.84, 26.59. HRMS (ESI) *m*/*z* calcd for C_37_H_25_N_3_O_5_NaS^+^ (M + Na)^+^ 646.1420, found 646.1413.

**1,5′-dimethyl-7′-(2-(thiophen-2-yl)ethyl)-5b′,8a′-dihydro-5′H,6′H-spiro[indoline-3,15′-[5a,8b]methanonaphtho[2,3-c]pyrrolo[3,4-a]carbazole]-2,6′,8′,9′,14′(7′H)-pentaone (3ay)**: A pink solid (49 mg, 79% yield); mp 235–237 °C; purified over a column of silica gel (petroleum ether/EtOAc = 3:1); ^1^H NMR (400 MHz, CDCl_3_) δ 8.52 (d, *J* = 7.8 Hz, 1H), 8.28 (d, *J* = 7.7 Hz, 1H), 8.00 (d, *J* = 7.7 Hz, 1H), 7.78 (t, *J* = 7.6 Hz, 1H), 7.66 (t, *J* = 7.5 Hz, 1H), 7.47 (t, *J* = 7.7 Hz, 1H), 7.11 (dd, *J* = 6.6, 5.9 Hz, 2H), 6.95 (t, *J* = 7.5 Hz, 1H), 6.86 (d, *J* = 4.9 Hz, 1H), 6.72 (dd, *J* = 6.5, 8.8 Hz, 4H), 6.55 (t, *J* = 7.7 Hz, 1H), 4.88 (d, *J* = 8.0 Hz, 1H), 4.63 (d, *J* = 8.0 Hz, 1H), 3.45 (d, *J* = 6.8 Hz, 2H), 3.34 (s, 3H), 3.02 (s, 3H), 2.84–2.66 (m, 2H). ^13^C NMR (101 MHz, CDCl_3_) δ 192.97, 178.23, 174.51, 173.43, 171.44, 162.73, 160.58, 143.71, 139.64, 137.25, 136.84, 135.08, 134.86, 133.33, 129.89, 128.53, 127.58, 127.38, 126.85, 125.51, 124.44, 123.94, 122.83, 121.78, 120.16, 119.89, 118.83, 108.97, 108.49, 85.90, 80.17, 69.14, 47.51, 41.02, 39.89, 30.86, 27.43, 26.61. HRMS (ESI) *m*/*z* calcd for C_38_H_27_N_3_O_5_NaS^+^ (M + Na)^+^ 660.1570, found 660.1569.

**7′-(1H-indol-5-yl)-1,5′-dimethyl-5b′,8a′-dihydro-5′H,6′H-spiro[indline-3,15′-[5a,8b]methanonaphtho[2,3-c]pyrrolo[3,4-a]carbazole]-2,6′,8′,9′,14′(7′H)-pentaone (3az):** A pink solid (34 mg, 53% yield); mp 272–274 °C; purified over a column of silica gel (petroleum ether/EtOAc = 3:1); ^1^H NMR (400 MHz, DMSO-*d_6_*) δ 11.24 (s, 1H), 8.49 (d, *J* = 7.7 Hz, 1H), 8.18 (d, *J* = 7.7 Hz, 1H), 7.89 (dd, *J* = 6.9, 7.6 Hz, 2H), 7.77 (t, *J* = 7.4 Hz, 1H), 7.48 (t, *J* = 7.6 Hz, 1H), 7.38–7.32 (m, 2H), 7.16 (d, *J* = 7.7 Hz, 1H), 7.02–6.90 (m, 4H), 6.57 (dt, *J* = 7.1, 8.0 Hz, 3H), 6.40 (s, 1H), 4.92 (d, *J* = 8.1 Hz, 1H), 4.65 (d, *J* = 8.0 Hz, 1H), 3.28 (s, 3H), 2.96 (s, 3H). ^13^C NMR (101 MHz, DMSO-*d_6_*) δ 193.59, 177.52, 175.54, 174.13, 170.43, 163.35, 161.37, 144.47, 137.44, 137.03, 136.96, 136.16, 135.62, 134.63, 134.44, 130.51, 127.88, 127.76, 127.67, 127.45, 127.24, 123.62, 123.56, 122.74, 122.03, 120.52, 120.02, 119.09, 119.00, 111.75, 110.03, 109.98, 109.83, 101.86, 85.22, 80.94, 68.56, 48.48, 41.47, 28.76, 26.93. HRMS (ESI) *m*/*z* calcd for C_40_H_26_N_4_O_5_Na^+^ (M + Na)^+^ 665.1804, found 665.1801.

**1,5′-diethyl-7′-methyl-5b′,8a′-dihydro-5′H,6′H-spiro[indoline-3,15′-[5a,8b]methanonaphtho[2,3-c]pyrrolo[3,4-a]carbazole]-2,6′,8′,9′,14′(7′H)-pentaone (4a)**: A pink solid (44 mg, 78% yield); mp 326–328 °C; purified over a column of silica gel (petroleum ether/EtOAc = 3:1); ^1^H NMR (400 MHz, CDCl_3_) δ 8.54 (d, *J* = 7.8 Hz, 1H), 8.26 (d, *J* = 7.8 Hz, 1H), 8.00 (d, *J* = 7.8 Hz, 1H), 7.76 (t, *J* = 7.5 Hz, 1H), 7.64 (t, *J* = 7.4 Hz, 1H), 7.43 (t, *J* = 7.7 Hz, 1H), 7.09 (t, *J* = 7.7 Hz, 1H), 6.91 (t, *J* = 7.5 Hz, 1H), 6.75 (d, *J* = 7.8 Hz, 1H), 6.64 (t, *J* = 6.8 Hz, 2H), 6.52 (t, *J* = 7.7 Hz, 1H), 4.80 (d, *J* = 7.9 Hz, 1H), 4.65 (d, *J* = 7.9 Hz, 1H), 3.89 (q, *J* = 7.5 Hz, 2H), 3.54–3.34 (m, 2H), 2.71 (s, 3H), 1.37 (t, *J* = 7.1 Hz, 3H), 0.96 (t, *J* = 7.1 Hz, 3H). ^13^C NMR (101 MHz, CDCl_3_) δ 193.27, 178.25, 174.80, 171.07, 161.33, 161.22, 142.69, 137.22, 136.78, 134.95, 134.81, 133.23, 129.80, 128.85, 127.46, 127.37, 124.66, 122.66, 122.14, 119.73, 119.17, 118.58, 108.43, 108.30, 85.46, 80.93, 69.21, 47.28, 41.53, 38.41, 34.93, 24.60, 13.82, 12.18. HRMS (ESI) *m*/*z* calcd for C_35_H_27_N_3_O_5_Na^+^ (M + Na)^+^ 592.1854, found 592.1848.

**1,2′,5,5′,7′-pentamethyl-5b′,8a′-dihydro-5′H,6′H-spiro[indoline-3,15′-[5a,8b]methanonaphtho[2,3-c]pyrrolo[3,4-a]carbazole]-2,6′,8′,9′,14′(7′H)-pentaone (4b)**: A pink solid (34 mg, 59% yield); mp 331–333 °C; purified over a column of silica gel (petroleum ether/EtOAc = 3:1); ^1^H NMR (400 MHz, CDCl_3_) δ 8.32–8.25 (m, 2H), 8.00 (d, *J* = 7.5 Hz, 1H), 7.79 (d, *J* = 7.9 Hz, 1H), 7.66 (d, *J* = 7.6 Hz, 1H), 7.28 (d, *J* = 8.0 Hz, 1H), 6.91 (d, *J* = 7.9 Hz, 1H), 6.65–6.59 (m, 2H), 6.45 (s, 1H), 4.86 (d, *J* = 8.0 Hz, 1H), 4.63 (d, *J* = 8.0 Hz, 1H), 3.31 (s, 3H), 2.99 (s, 3H), 2.70 (s, 3H), 2.38 (s, 3H), 1.88 (s, 3H). ^13^C NMR (101 MHz, CDCl_3_) δ 193.51, 178.15, 174.63, 173.93, 171.33, 161.21, 141.34, 138.35, 137.33, 134.98, 134.93, 133.20, 132.08, 130.21, 129.26, 128.20, 127.42, 127.36, 125.24, 121.90, 119.38, 118.97, 108.78, 108.13, 85.90, 80.28, 68.99, 47.73, 41.19, 31.03, 26.60, 24.61, 21.22, 20.79. HRMS (ESI) *m*/*z* calcd for C_35_H_27_N_3_O_5_Na^+^ (M + Na)^+^ 592.1851, found 592.1848.

**2′,5-difluoro-1,5′,7′-trimethyl-5b′,8a′-dihydro-5′H,6′H-spiro[indoline-3,15′-[5a,8b]methanonaphtho[2,3-c]pyrrolo[3,4-a]carbazole]-2,6′,8′,9′,14′(7′H)-pentaone (4c)**: A pink solid (32 mg, 55% yield); mp 232–234 °C; purified over a column of silica gel (petroleum ether/EtOAc = 3:1); ^1^H NMR (400 MHz, CDCl_3_) δ 8.25 (dd, *J* = 6.0, 6.7 Hz, 2H), 8.01 (d, *J* = 7.7 Hz, 1H), 7.81 (t, *J* = 7.2 Hz, 1H), 7.69 (t, *J* = 7.1 Hz, 1H), 7.24–7.16 (m, 1H), 6.84 (dd, *J* = 8.6, 6.6 Hz, 1H), 6.66 (ddd, *J* = 6.9, 8.7, 3.8 Hz, 2H), 6.39 (d, *J* = 6.7 Hz, 1H), 4.87 (d, *J* = 8.0 Hz, 1H), 4.64 (d, *J* = 7.9 Hz, 1H), 3.32 (s, 3H), 3.00 (s, 3H), 2.72 (s, 3H). ^13^C NMR (101 MHz, CDCl_3_) δ 193.51, 178.15, 174.63 (d, *J_F_* = 70 Hz), 173.93, 171.33, 161.32 (d, *J_F_* = 4 Hz), 161.28, 141.34, 138.35, 137.33, 134.98 (d, *J_F_* = 5 Hz), 134.93, 133.20 (d, *J_F_* = 112 Hz), 132.08, 130.21, 129.26, 128.20, 127.42 (d, *J_F_* = 6 Hz), 127.36, 125.24, 121.90, 119.38, 118.97, 108.78 (d, *J_F_* = 65 Hz), 108.13, 85.90, 80.28, 68.99, 47.73, 41.19, 31.03, 26.60, 24.61, 21.22, 20.79. ^1^HRMS (ESI) *m*/*z* calcd for C_33_H_21_N_3_O_5_NaF_2_^+^ (M + Na)^+^ 600.1353, found 600.1347.

**2′,5-dichloro-1,5′,7′-trimethyl-5b′,8a′-dihydro-5′H,6′H-spiro[indoline-3,15′-[5a,8b]methanonaphtho[2,3-c]pyrrolo[3,4-a]carbazole]-2,6′,8′,9′,14′(7′H)-pentaone (4d)**: A pink solid (32 mg, 52% yield); mp 240–242 °C; purified over a column of silica gel (petroleum ether/EtOAc = 3:1); ^1^H NMR (400 MHz, CDCl_3_) δ 8.55 (s, 1H), 8.32 (d, *J* = 7.6 Hz, 1H), 8.06 (d, *J* = 7.6 Hz, 1H), 7.87 (t, *J* = 7.5 Hz, 1H), 7.75 (t, *J* = 7.1 Hz, 1H), 7.46 (d, *J* = 8.2 Hz, 1H), 7.17 (d, *J* = 8.0 Hz, 1H), 6.76–6.63 (m, 3H), 4.89 (d, *J* = 7.8 Hz, 1H), 4.67 (d, *J* = 7.9 Hz, 1H), 3.37 (s, 3H), 3.04 (s, 3H), 2.77 (s, 3H). ^13^C NMR (101 MHz, CDCl_3_) δ 192.78, 178.13, 174.40, 173.45, 170.86, 160.88, 158.79, 142.40, 136.91, 136.73, 135.38, 134.79, 133.72, 130.17, 128.12, 127.73, 127.62, 127.51, 125.13, 124.64, 123.35, 120.89, 119.51, 110.07, 109.52, 85.92, 80.33, 69.41, 47.24, 41.11, 31.05, 26.78, 24.76. HRMS (ESI) *m*/*z* calcd for C_33_H_21_N_3_O_5_NaCl_2_^+^ (M + Na)^+^ 632.0759, found 632.0756.

**3′,6-dimethoxy-1,5′,7′-trimethyl-5b′,8a′-dihydro-5′H,6′H-spiro[indline-3,15′-[5a,8b]methanonaphtho[2,3-c]pyrrolo[3,4-a]carbazole]-2,6′,8′,9′,14′(7′H)-pentaone (4e)**: An orange solid (44 mg, 73% yield); mp 211–213 °C; purified over a column of silica gel (petroleum ether/EtOAc = 3:1); ^1^H NMR (400 MHz, CDCl_3_) δ 8.55 (t, *J* = 9.3 Hz, 1H), 8.33 (d, *J* = 7.6 Hz, 1H), 8.07 (d, *J* = 7.5 Hz, 1H), 7.85 (t, *J* = 7.0 Hz, 1H), 7.75–7.69 (m, 1H), 6.69–6.59 (m, 2H), 6.39 (s, 1H), 6.23–6.08 (m, 2H), 4.90 (d, *J* = 8.0 Hz, 1H), 4.67 (d, *J* = 8.0 Hz, 1H), 3.94 (s, 3H), 3.71 (s, 3H), 3.40 (s, 3H), 3.09 (s, 3H), 2.81 (s, 3H). ^13^C NMR (101 MHz, CDCl_3_) δ 193.62, 177.75, 175.03, 173.96, 171.97, 167.71, 165.12, 161.02, 160.26, 137.40, 134.93, 132.68, 129.93, 127.25, 125.35, 116.36, 113.78, 113.15, 112.40, 109.06, 106.31, 96.42, 92.62, 86.21, 69.00, 55.63, 55.24, 47.93, 41.37, 30.64, 29.68, 26.59, 24.59. HRMS (ESI) *m*/*z* calcd for C_35_H_27_N_3_O_7_Na^+^ (M + Na)^+^ 624.1752, found 624.1747.

**3′,6-dichloro-1,5′,7′-trimethyl-5b′,8a′-dihydro-5′H,6′H-spiro[indoline-3,15′-[5a,8b]methanonaphtho[2,3-c]pyrrolo[3,4-a]carbazole]-2,6′,8′,9′,14′(7′H)-pentaone (4f)**: An orange solid (40 mg, 66% yield); mp 327–329 °C; purified over a column of silica gel (petroleum ether/EtOAc = 3:1); ^1^H NMR (400 MHz, CDCl_3_) δ 8.43 (d, *J* = 8.3 Hz, 1H), 8.24 (d, *J* = 7.8 Hz, 1H), 8.00 (d, *J* = 7.8 Hz, 1H), 7.80 (d, *J* = 7.3 Hz, 1H), 7.69 (d, *J* = 7.5 Hz, 1H), 6.91 (d, *J* = 8.3 Hz, 1H), 6.75 (s, 1H), 6.67 (s, 1H), 6.54 (s, 2H), 4.83 (d, *J* = 8.0 Hz, 1H), 4.61 (d, *J* = 8.0 Hz, 1H), 3.32 (s, 3H), 2.98 (s, 3H), 2.72 (s, 3H). ^13^C NMR (101 MHz, CDCl_3_) δ 192.96, 178.33, 174.48, 173.42, 170.93, 162.47, 159.03, 144.82, 143.46, 136.86, 136.01, 135.25, 134.68, 133.63, 129.28, 127.58, 127.53, 125.18, 122.78, 120.66, 120.38, 119.89, 117.22, 109.39, 109.26, 85.83, 80.28, 69.21, 47.32, 41.19, 30.74, 26.75, 24.70. HRMS (ESI) *m*/*z* calcd for C_33_H_21_N_3_O_5_NaCl_2_^+^ (M + Na)^+^ 632.0748, found 632.0756.

**3′,6-dibromo-1,5′,7′-trimethyl-5b′,8a′-dihydro-5′H,6′H-spiro[indolne-3,15′-[5a,8b]methanonaphtho[2,3-c]pyrrolo[3,4-a]carbazole]-2,6′,8′,9′,14′(7′H)-pentaone (4g)**: An orange solid (42 mg, 60% yield); mp 351–353 °C; purified over a column of silica gel (petroleum ether/EtOAc = 3:1); ^1^H NMR (400 MHz, CDCl_3_) δ 8.35 (d, *J* = 8.3 Hz, 1H), 8.24 (d, *J* = 7.8 Hz, 1H), 8.01 (d, *J* = 7.6 Hz, 1H), 7.79 (t, *J* = 7.6 Hz, 1H), 7.69 (t, *J* = 7.4 Hz, 1H), 7.07 (d, *J* = 8.3 Hz, 1H), 6.87 (d, *J* = 7.1 Hz, 2H), 6.70 (d, *J* = 8.3 Hz, 1H), 6.47 (d, *J* = 8.2 Hz, 1H), 4.83 (d, *J* = 8.0 Hz, 1H), 4.60 (d, *J* = 8.0 Hz, 1H), 3.31 (s, 3H), 2.98 (s, 3H), 2.72 (s, 3H). ^13^C NMR (101 MHz, CDCl_3_) δ 192.77, 178.28, 178.24, 174.50, 173.54, 173.45, 171.03, 162.54, 159.29, 144.86, 136.93, 135.26, 134.74, 133.65, 132.32, 129.29, 127.60, 127.54, 125.75, 125.45, 124.01, 123.45, 120.68, 120.47, 119.51, 117.59, 112.32, 112.18, 85.62, 80.53, 69.13, 47.26, 41.19, 30.73, 26.75, 24.70. HRMS (ESI) *m*/*z* calcd for C_33_H_21_N_3_O_5_NaBr_2_^+^ (M + Na)^+^ 719.9745, found 719.9746.

**1,4′,5′,7,7′-pentamethyl-5b′,8a′-dihydro-5′H,6′H-spiro[indoline-3,15′-[5a,8b]methanonaphtho[2,3-c]pyrrolo[3,4-a]carbazole]-2,6′,8′,9′,14′(7′H)-pentaone (4h)**: A pink solid (37 mg, 65% yield); mp 286–288 °C; purified over a column of silica gel (petroleum ether/EtOAc = 3:1); ^1^H NMR (400 MHz, CDCl_3_) δ 8.48 (d, *J* = 8.0 Hz, 1H), 8.25 (d, *J* = 7.7 Hz, 1H), 7.99 (d, *J* = 7.6 Hz, 1H), 7.76 (t, *J* = 7.5 Hz, 1H), 7.64 (t, *J* = 7.6 Hz, 1H), 7.18 (d, *J* = 7.4 Hz, 1H), 6.89–6.81 (m, 2H), 6.50 (d, *J* = 7.9 Hz, 1H), 6.40 (t, *J* = 7.7 Hz, 1H), 4.92 (d, *J* = 7.9 Hz, 1H), 4.62 (d, *J* = 7.9 Hz, 1H), 3.63 (s, 3H), 3.28 (s, 3H), 2.71 (s, 3H), 2.51 (s, 3H), 2.42 (s, 3H). ^13^C NMR (101 MHz, CDCl_3_) δ 193.19, 178.31, 174.86, 173.70, 172.51, 161.32, 141.59, 139.97, 137.24, 136.98, 134.97, 134.70, 133.73, 133.14, 127.52, 127.29, 126.77, 122.55, 122.46, 122.29, 120.71, 120.32, 120.18, 119.84, 119.30, 86.46, 79.94, 69.28, 47.93, 41.50, 34.38, 30.18, 24.58, 19.77, 19.52. HRMS (ESI) *m*/*z* calcd for C_35_H_27_N_3_O_5_Na^+^ (M + Na)^+^ 592.1846, found 592.1848.

**7′-phenyl-1,5′-dipropyl-5b′,8a′-dihydro-5′H,6′H-spiro[indoline-3,15′-[5a,8b]methanonaphtho[2,3-c]pyrrolo[3,4-a]carbazole]-2,6′,8′,9′,14′(7′H)-pentaone (4j)**: A pink solid (38 mg, 58% yield); mp 341–343 °C; purified over a column of silica gel (petroleum ether/EtOAc = 3:1); ^1^H NMR (400 MHz, DMSO-*d_6_*) δ 8.48 (d, *J* = 7.6 Hz, 1H), 8.19–8.15 (m, 1H), 7.87 (dd, *J* = 22.9, 7.3 Hz, 2H), 7.73 (d, *J* = 7.3 Hz, 1H), 7.44 (t, *J* = 7.4 Hz, 1H), 7.33 (dd, *J* = 15.1, 7.4 Hz, 3H), 7.14–7.08 (m, 1H), 6.89 (ddd, *J* = 7.6, 8.1, 6.2 Hz, 5H), 6.54 (t, *J* = 6.1 Hz, 2H), 4.90 (d, *J* = 8.0 Hz, 1H), 4.64 (d, *J* = 7.9 Hz, 1H), 3.84–3.60 (m, 3H), 3.45–3.38 (m, 1H), 1.71 (dd, *J* = 14.5, 7.4 Hz, 2H), 1.28–1.23 (m, 2H), 1.04 (t, *J* = 7.4 Hz, 3H), 0.54 (t, *J* = 7.3 Hz, 3H). ^13^C NMR (101 MHz, DMSO-*d_6_*) δ 193.10, 177.80, 174.48, 172.90, 170.84, 162.47, 161.41, 143.66, 137.28, 136.98, 135.80, 134.66, 134.09, 132.09, 130.49, 129.33, 128.99, 127.97, 127.58, 127.33, 127.18, 123.90, 122.53, 121.90, 119.45, 118.46, 112.53, 109.55, 85.92, 81.08, 69.30, 48.03, 45.85, 41.81, 22.22, 20.67, 11.77, 10.88. HRMS (ESI) *m*/*z* calcd for C_42_H_33_N_3_O_5_Na^+^ (M + Na)^+^ 682.2325, found 682.2318.

**1,3′,5′,6-tetramethyl-7′-phenyl-5b′,8a′-dihydro-5′H,6′H-spiro[indolne-3,15′-[5a,8b]methanonaphtho[2,3-c]pyrrolo[3,4-a]carbazole]-2,6′,8′,9′,14′(7′H)-pentaone (4k)**: An orange solid (41 mg, 65% yield); mp 243–245 °C; purified over a column of silica gel (petroleum ether/EtOAc = 3:1); ^1^H NMR (400 MHz, CDCl_3_) δ 8.49 (d, *J* = 8.0 Hz, 1H), 8.24 (d, *J* = 7.8 Hz, 1H), 7.98 (d, *J* = 7.7 Hz, 1H), 7.72 (t, *J* = 7.6 Hz, 1H), 7.60 (t, *J* = 7.5 Hz, 1H), 7.38–7.21 (m, 3H), 6.93 (d, *J* = 7.5 Hz, 2H), 6.78 (d, *J* = 8.1 Hz, 1H), 6.63–6.55 (m, 2H), 6.47 (s, 1H), 6.37 (d, *J* = 7.9 Hz, 1H), 5.01 (d, *J* = 8.1 Hz, 1H), 4.77 (d, *J* = 8.1 Hz, 1H), 3.34 (s, 3H), 3.02 (s, 3H), 2.35 (s, 3H), 2.17 (s, 3H). ^13^C NMR (101 MHz, CDCl_3_) δ 193.47, 178.22, 174.12, 173.03, 171.84, 163.29, 160.81, 148.90, 143.88, 140.25, 137.32, 134.94, 133.06, 131.30, 129.21, 128.73, 128.16, 127.54, 127.31, 126.63, 124.24, 123.43, 121.77, 118.90, 118.76, 116.90, 109.43, 86.51, 79.75, 69.51, 47.86, 41.30, 30.79, 26.59, 22.96, 21.60. HRMS (ESI) *m*/*z* calcd for C_40_H_29_N_3_O_5_Na^+^ (M + Na)^+^ 654.2012, found 654.2005.

**3′,6-dimethoxy-1,5′-dimethyl-7′-phenyl-5b′,8a′-dihydro-5′H,6′H-spiro[indoline-3,15′-[5a,8b]methanonaphtho[2,3-c]pyrrolo[3,4-a]carbazole]-2,6′,8′,9′,14′(7′H)-pentaone (4l):** An orange solid (48 mg, 73% yield); mp 232–234 °C; purified over a column of silica gel (petroleum ether/EtOAc = 3:1); ^1^H NMR (400 MHz, CDCl_3_) δ 8.54 (d, *J* = 8.7 Hz, 1H), 8.23 (d, *J* = 7.6 Hz, 1H), 7.98 (d, *J* = 7.7 Hz, 1H), 7.72 (t, *J* = 7.4 Hz, 1H), 7.59 (t, *J* = 7.4 Hz, 1H), 7.39–7.20 (m, 3H), 6.94 (d, *J* = 7.4 Hz, 2H), 6.64 (d, *J* = 8.5 Hz, 1H), 6.52 (d, *J* = 8.7 Hz, 1H), 6.32 (s, 1H), 6.07 (d, *J* = 8.8 Hz, 2H), 4.97 (d, *J* = 8.1 Hz, 1H), 4.74 (d, *J* = 8.1 Hz, 1H), 3.83 (s, 3H), 3.64 (s, 3H), 3.33 (s, 3H), 3.02 (s, 3H). ^13^C NMR (101 MHz, CDCl_3_) δ 193.77, 173.99, 173.31, 172.13, 167.80, 161.22, 160.24, 145.20, 137.44, 134.90, 134.80, 132.92, 132.45, 130.07, 129.22, 128.61, 127.40, 127.24, 126.63, 125.55, 116.43, 113.66, 112.98, 110.02, 109.14, 106.50, 96.66, 92.78, 86.84, 55.57, 55.26, 48.03, 41.27, 30.65, 26.56. HRMS (ESI) *m*/*z* calcd for C_40_H_29_N_3_O_7_Na^+^ (M + Na)^+^ 686.1898, found 686.1903.

**3′,6-difluoro-1,5′-dimethyl-7′-phenyl-5b′,8a′-dihydro-5′H,6′H-spro[indoline-3,15′-[5a,8b]methanonaphtho[2,3-c]pyrrolo[3,4-a]carbazole]-2,6′,8′,9′,14′(7′H)-pentaone (4m)**: An orange solid (35 mg, 54% yield); mp 165–167 °C; purified over a column of silica gel (petroleum ether/EtOAc = 3:1); ^1^H NMR (400 MHz, CDCl_3_) δ 8.61 (dd, *J* = 8.6, 5.9 Hz, 1H), 8.24 (d, *J* = 7.7 Hz, 1H), 8.00 (d, *J* = 7.6 Hz, 1H), 7.70 (dt, *J* = 43.3, 7.5 Hz, 3H), 7.32 (dt, *J* = 21.8, 7.2 Hz, 2H), 6.92 (d, *J* = 7.3 Hz, 2H), 6.67 (dd, *J* = 8.0, 6.0 Hz, 2H), 6.51 (dd, *J* = 8.5, 2.1 Hz, 1H), 6.37–6.26 (m, 2H), 4.99 (d, *J* = 8.1 Hz, 1H), 4.76 (d, *J* = 8.1 Hz, 1H), 3.34 (s, 3H), 3.02 (s, 3H). ^13^C NMR (101 MHz, CDCl_3_) δ 192.84, 178.38, 173.88, 172.65 (d, *J_F_* = 119 Hz), 171.46, 170.58, 167.85 (d, *J_F_* = 279 Hz), 165.06, 164.47, 164.25, 159.35, 145.53, 136.99, 135.19, 134.86, 133.49, 131.17(d, *J_F_* = 59 Hz), 130.58, 130.46, 129.29, 128.89, 127.65 (d, *J_F_* = 20 Hz), 127.45, 126.50, 125.76 (d, *J_F_* = 9 Hz), 125.67 (d, *J* = 19.8 Hz), 109.18, 108.96 (d, *J_F_* = 22 Hz), 108.67, 108.40, 97.67, 97.39, 96.68, 96.39, 86.81, 69.60, 47.54, 41.23, 30.85, 29.68, 26.81. ^19^F NMR (376 MHz, CDCl_3_) δ −96.76, −108.84. HRMS (ESI) *m*/*z* calcd for C_38_H_23_N_3_O_5_NaF_2_^+^ (M + Na)^+^ 662.1500, found 662.1503.

**3′,6-dichloro-1,5′-dimethyl-7′-phenyl-5b′,8a′-dihydro-5′H,6′H-spro[indoline-3,15′-[5a,8b]methanonaphtho[2,3-c]pyrrolo[3,4-a]carbazole]-2,6′,8′,9′,14′(7′H)-pentaone (4n)**: An orange solid (46 mg, 68% yield); mp 347–349 °C; purified over a column of silica gel (petroleum ether/EtOAc = 3:1); ^1^H NMR (400 MHz, CDCl_3_) δ 8.52 (d, *J* = 8.1 Hz, 1H), 8.24 (d, *J* = 7.2 Hz, 1H), 8.00 (d, *J* = 7.3 Hz, 1H), 7.70 (dd, *J* = 34.4, 7.1 Hz, 2H), 7.32 (dd, *J* = 14.1, 6.7 Hz, 3H), 6.91 (s, 3H), 6.77 (s, 1H), 6.62 (dd, *J* = 22.1, 14.2 Hz, 3H), 4.99 (d, *J* = 7.8 Hz, 1H), 4.77 (d, *J* = 7.7 Hz, 1H), 3.34 (s, 3H), 3.01 (s, 3H). ^13^C NMR (101 MHz, CDCl_3_) δ 192.84, 178.33, 173.83, 172.60, 171.11, 162.68, 159.21, 144.95, 143.63, 136.91, 136.08, 135.25, 134.86, 133.63, 131.09, 129.31, 129.09, 128.93, 127.71, 127.52, 126.47, 125.22, 122.80, 120.88, 120.56, 119.87, 117.35, 109.45, 109.40, 86.24, 79.50, 69.53, 47.41, 41.23, 30.83, 26.79. HRMS (ESI) *m*/*z* calcd for C_38_H_23_N_3_O_5_NaCl^+^ (M + Na)^+^ 694.0917, found 694.0912.

## 4. Conclusions

In summary, a strategy for the direct synthesis of novel polycyclic *N*-heterocycle-fused naphthoquinones by merging intramolecular oxidative coupling and cascade [4 + 2] cycloaddition is presented. In the protocol, mechanistic investigations suggest that the cascade reaction involves the intermediate spiro polycyclic *N*-heterocycles and [4 + 2] cycloaddition processes. The notable features of this method include easily obtainable substrates and compatibility of good functional groups in the formation of the products. Further application of the method for the preparation of new luminescent material molecules is ongoing in our group. The synthetic compounds exhibit interesting fluorescence properties, and the selective sensing of TFA experiments was conducted to show the application in fluorescent probes.

## Figures and Tables

**Table 1:** Optimization of the reaction conditions ^a^.

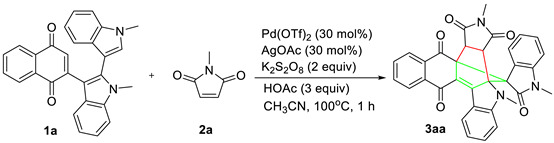		
**Entry**	**Variation from the Standard Conditions**	**Yield (%)** **^b^ 3a** **a**
1	Standard conditions	82
2	Other palladium catalyst instead of Pd(OTf)_2_	<50
3	Other catalyst instead of AgOAc	<40
4	Other oxidants instead of K_2_S_2_O_8_	<60
5	1 equiv instead of 3 equiv of HOAc	76
6	80 °C instead of 100 °C	61
7	DMSO instead of CH_3_CN	<10

^a^ Standard conditions: **1a** (0.1 mmol), **2a** (0.3 mmol) Pd(OTf)_2_ (30 mol%), K_2_S_2_O_8_ (2 equiv), AgOAc (30 mol%), HOAc (3 equiv), CH_3_CN (2 mL), 100 °C, sealed tube for 1 h. ^b^ Isolated yields.

**Table 2:** Scope of maleimides ^a^.

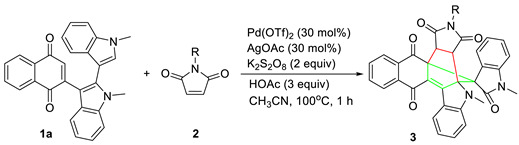
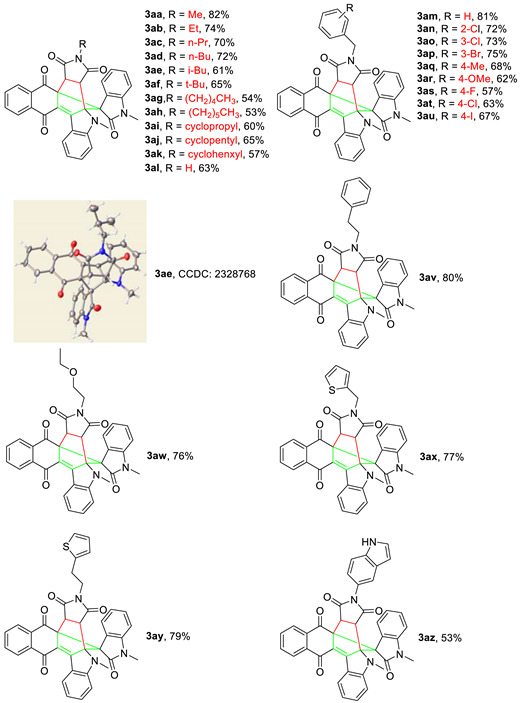

^a^ Reaction conditions: **1a** (0.1 mmol), **2** (0.3 mmol) Pd(OTf)_2_ (30 mol%), K_2_S_2_O_8_ (2 equiv), AgOAc (30 mol%), HOAc (3 equiv), CH_3_CN (2 mL), 100 °C, sealed tube for 1 h. Isolated yields are indicated.

**Table 3:** Scope for bi-indolylnaphthoquinones ^a^.

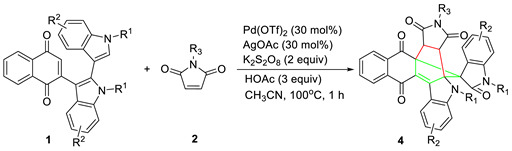
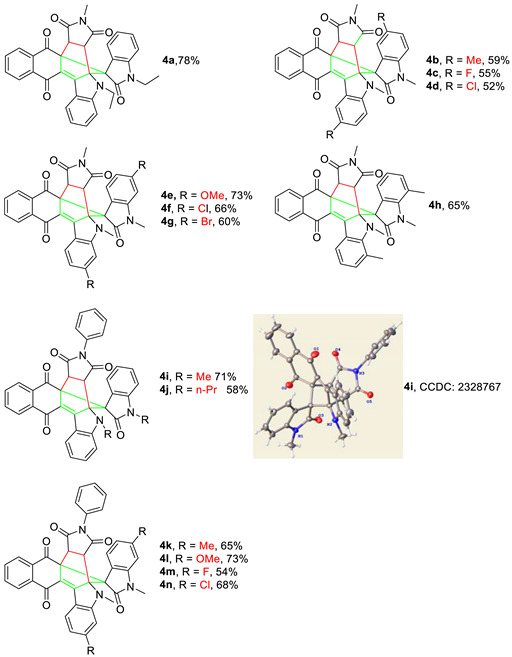

^a^ Reaction conditions: **1** (0.1 mmol), **2** (0.3 mmol) Pd(OTf)_2_ (30 mol%), K_2_S_2_O_8_ (2 equiv), AgOAc (30 mol%), HOAc (3 equiv), CH_3_CN (2 mL), 100 °C, sealed tube for 1 h. Isolated yields are indicated.

## Data Availability

The original contributions presented in the study are included in the article/Supplementary Materials, further inquiries can be directed to the corresponding author/s.

## Supplementary Materials

The following supporting information can be downloaded at https://www.mdpi.com/article/10.3390/molecules29235639/s1: Figure S1: DFT calculation; Figures S2–S8: Experimental Section for Photo-Physical Studies; Figures S9–S10: Crystal data and structure refinement of product **5ae** and **6i**; Figures S11–S92: Copies of ^1^H, ^13^C, and ^19^F NMR spectra for all compounds. Tables S1–S6: Optimization of reaction conditions; Table S7: Photophysical characterization data of all compounds; Tables S8–S22: Crystal data and structure refinement of product 5ae and 6i (see [36,37,38]).
