# Supplementary material for: Pd/Ag-Cocatalyzed Merging Intramolecular Oxidative Coupling and Cascade [4 + 2] Cycloaddition: Synthesis and Photophysical Properties of Novel Polycyclic N-Heterocycles Fused Naphthoquinones
Source: Molecules. 2024 Nov 28;29(23):5639. doi: 10.3390/molecules29235639 (PMC11643544; doi:10.3390/molecules29235639)

Pd/Ag-Cocatalyzed Merging Intramolecular Oxidative Coupling and Cascade [4 + 2] Cycloaddition: Synthesis and Photophysical Properties of Novel Polycyclic *N*-Heterocycles Fused Naphthoquinones

Yu Dong [1], Lin Chen [1], Han-Qing Wu [1], Li Xie [2], Jing-Hao Yu [1], Fan Yang [1], Yu-Ting Wang [1], Yu-Rong Liu [1], Guo-Wei Deng [1,*] and Zhi-Fan Wang [1,*]

[1] Sichuan Provincial Key Laboratory for Structural Optimization and Application of Functional Molecules, College of Chemistry and Life Science, Chengdu Normal University, Chengdu 611130, China; ssdy1990@126.com (Y.D.); cl13649000476@163.com (L.C.); 18070442587@163.com (J.-H.Y.); 18153805241@163.com (F.Y.); yutingwang0919@126.com (Y.-T.W.); 18884569093@163.com (Y.-R.L.)

[2] Chengdu Institute for Drug Control, Chengdu 610061, China

* Correspondence: guoweideng86@163.com (G.-W.D.); wangzhifan1988@gmail.com (Z.-F.W.)

Table of Content

1. General information **S2**

2. Preparation of the starting materials **S2**

3. Optimization of reaction conditions **S6**

4. DFT calculation **S10**

5. Experimental Section for Photo-Physical Studies **S12**

6. Crystal data and structure refinement of product 5ae **S16**

7. Crystal data and structure refinement of product 6i **S26**

8. References **S36**

9. Copies of ^{1}H, ^{13}C and ^{19}F NMR spectra for all compound **S37**

1 General information

Measurement of fluorescence quantum yield (Φ_F)

Fluorescence quantum yields (Φ_F) of our synthesized polycyclic *N*-heterocycles fused naphthoquinones were calculated using rhodamine 6G as a standard (Φ = 0.95). Emission spectra of **3** or **4** solutions were recorded from 500 to 825 nm with excitation at λ_{max}(nm). Absorbance (optical density, OD) of all the samples **3** or **4**were recorded at λ_{max}(nm) and quantum yields were calculated according to equation (1), in which Φ_{ref} is the quantum yield of the reference, A_{sample} and A_{ref} are the areas under the emission spectra of the sample **3** or **4** and the reference, respectively, and OD_{ref} and OD_{sample} are the absorbances of the reference and the sample **3** or **4**, respectively, measured at the excitation wavelength; λ_{sample} and λ_{ref} are the refractive indices of the sample **3** or **4** and the reference, respectively, in solution.

$$\emptyset_{sample} = \emptyset_{ref}\left(\frac{A_{sample}}{A_{ref}}\right) \times \left(\frac{OD_{ref}}{OD_{sample}}\right) \times \left(\frac{\lambda_{ref}}{\lambda_{sample}}\right) \quad (1)$$

2 Preparation of the starting materials

a) **The synthesis of *N*-protected indoles** [1]

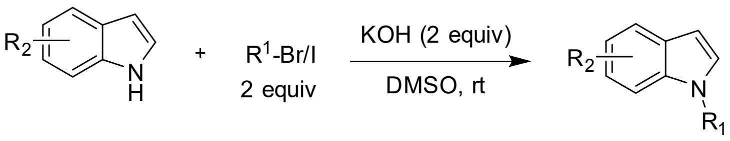

General procedure for the synthesis of *N*-protected indoles from substituted indoles with alkyl bromides (iodomethane used for methyl-protected reagent). A 50 mL flask equipped with a stir-bar was charged with indole (2.5 mmol) and KOH (5.0 mmol). 10 mL of DMSO was added to the flask and the solution was stirred under room temperature, then alkyl bromides (5.0 mmol) was added. The reaction mixture was stirred at room temperature and monitored by TLC. Upon finished the reaction mixture was quenched by water (10 mL) and extracted by ethyl acetate (3×15 mL). Combined organic phase were dried over anhydrous Na_2SO_4, and concentrated under reduced pressure. The residue was then purified by chromatography on silica gel with a mixture eluent of petroleum ether, ethyl acetate.

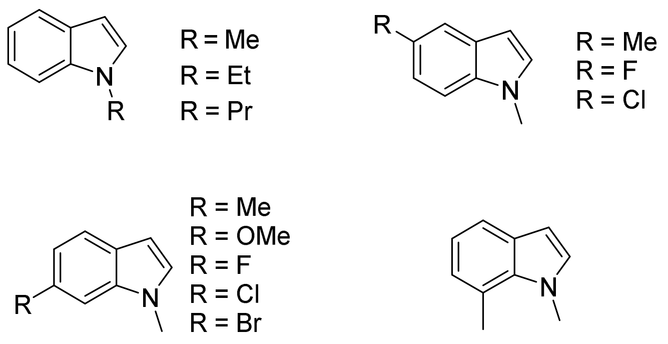

b) The synthesis of bi-indolnaphthoquinones 3 [2]

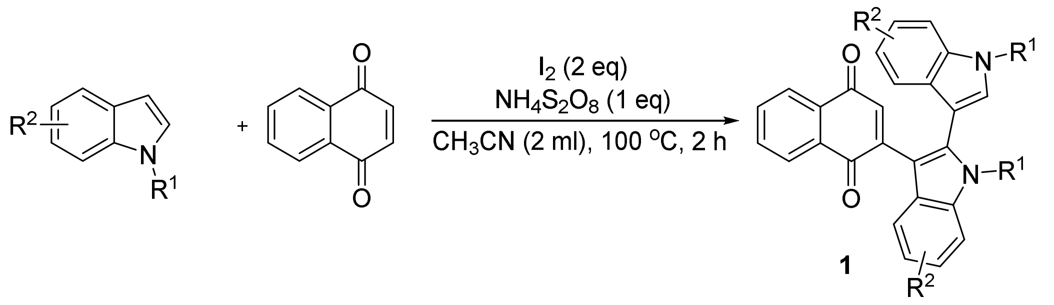

To a solution of indoles (0.6 mmol), and I_2 (0.6 mmol), $(NH_4)_2S_2O_8$ (0.3 mmol), in CH_3CN (2 mL) was added naphthoquinones (0.3 mmol, 1 equiv). The reaction mixture was stirred at 100 °C under sealed tube for 2 h. After the completion of the reaction (monitored by TLC). The reaction was quenched with saturated salt water (6 ml) and the mixture was extracted with EtOAc (3 × 3 mL). The organic extracts were washed with brine, dried over Na_2SO_4, filtered and the solvent was removed in vacuo. The crude product was purified by silica gel column chromatography to give bi-indolnaphthoquinones **1**.

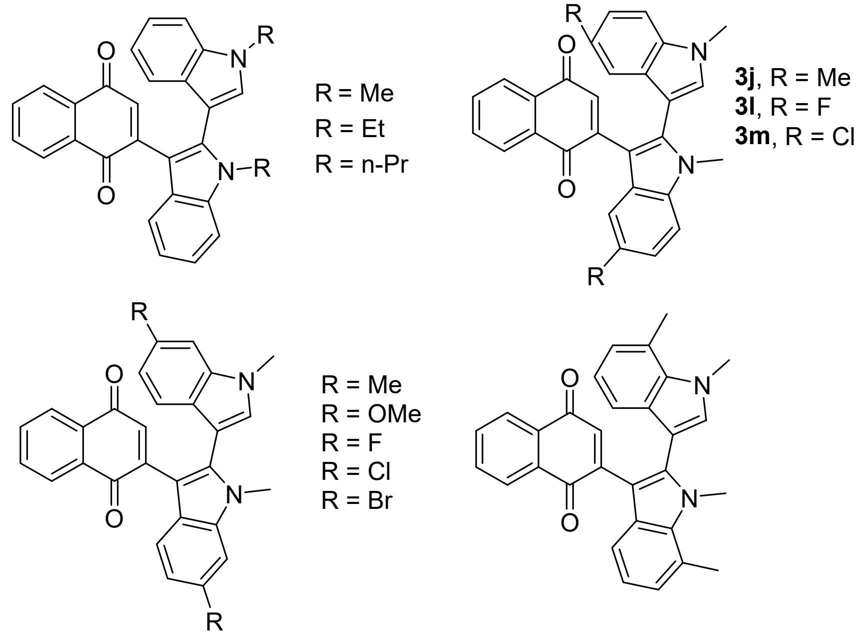

c) The synthesis of *N*-aryl/*N*-alkyl maleimide derivatives [3]

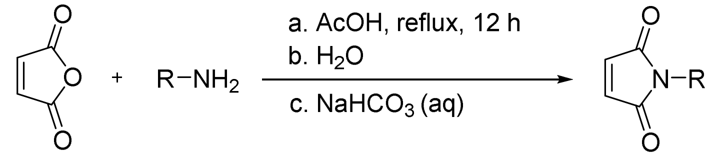

2

Compound **2** was prepared according to literature report.[2] Maleic anhydride (2.0 equiv.) and primary amine (10 mmol.) were added in a 50 mL flask with 15 mL AcOH. The reaction mixture was refluxed for 12 h at 125 °C. Progress of the reaction was monitored by TLC, the mixture was moved to room temperature after completion of the reaction. Saturated $NaHCO_3$ was slowly add to adjust the pH to around 9, extracted with ethyl acetate (3 × 40 mL), and dried over anhydrous Na_2SO_4. Then the solvent was completely removed, and **2** was purified by column chromatography.

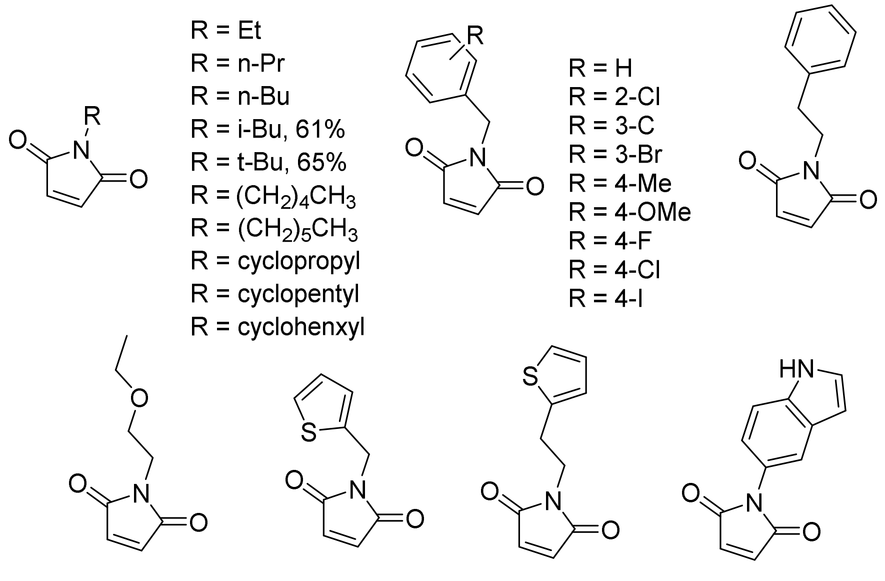

d) The synthesis of 5

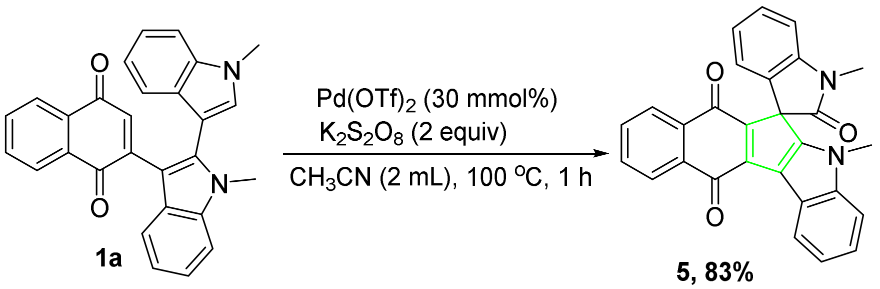

To a solution of bi-indolnaphthoquinones **1a** (0.1 mmol), and $Pd(OTf)_2$ (0.03 mmol), in CH_3CN (2 mL) was added $K_2S_2O_8$ (0.2 mmol). The reaction mixture was stirred at 100 °C under sealed tube for 1 h. After the completion of the reaction (monitored by TLC). The reaction was quenched with saturated salt water (6 ml) and the mixture was extracted with EtOAc (3 × 3 mL). The organic extracts were washed with brine, dried over Na_2SO_4, filtered and the solvent was removed in vacuo. The crude product was purified by silica gel column chromatography to give **5**.

3 Optimization of reaction conditions

Table S1. Optimization of Pd-Catalyst[a, b]

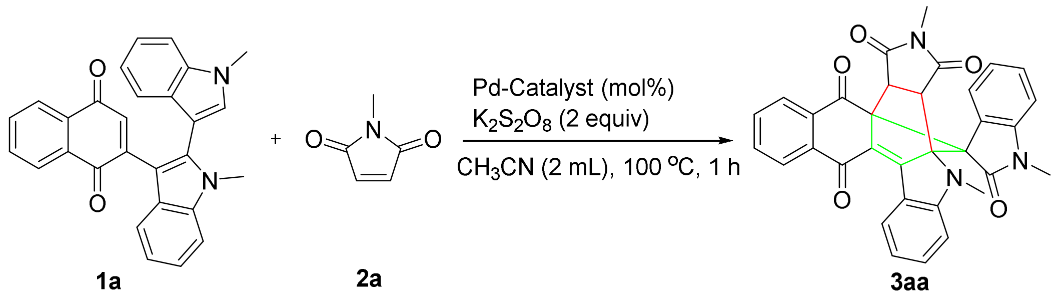

Entry	Pd-Catalyst (mol%)	Yield (%)
1	$Pd(OTf)_2$ (30)	61
2	$PdCl_2$ (30)	15
3	$Pd(OAc)_2$ (30)	46
4	$Pd(PPh_3)_4$ (30)	12
5	$Pd(PPh)_2(OAc)_2$ (30)	18
6	No	15
7	$Pd(OTf)_2$ (10)	23
8	$Pd(OTf)_2$ (20)	30
9	$Pd(OTf)_2$ (40)	40

[a]Reaction conditions: **1a** (0.1 mmol), **2a** (0.3 mmol), catalysts (mmol%), AgOAc(30 mmol%), $K_2S_2O_8$ (3 equiv), CH_3CN (2 mL) as solvent, 100 °C, sealed tube for 1 h, [b]Isolated yields.

Table S2. Optimization of Catalyst[a, b]

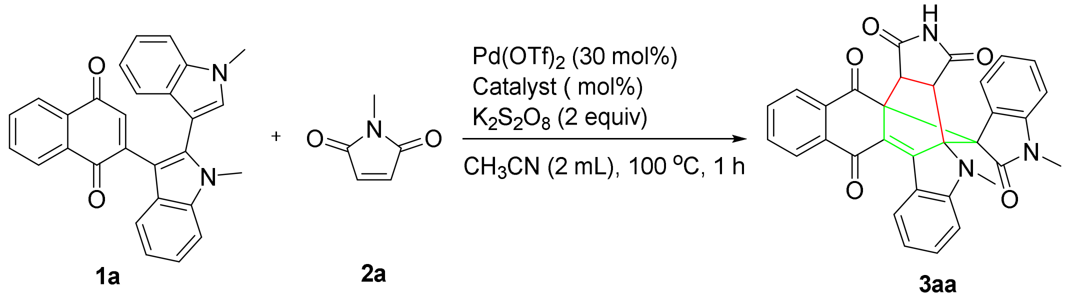

Entry	Catalyst (mol%)	Yield (%)
1	AgOAc (30)	73
2	$AgNO_3$ (30)	35
3	$Zn(OAc)_2$ (30)	Trace

4	$ZnCl_2$ (30)	16
5	$Cu(OAc)_2$ (30)	Trace
6	CuI (30)	18
7	$CuSO_4.5H_2O$ (30)	16
8	$Ni(OAc)_2$ (30)	18
9	MnO_2 (30)	25
10	$MnSO_4$ (30)	Trace
11	$FeCl_3$ (30)	ND[c]
12	$FeSO_4.7H_2O$ (30)	Trace
13	$AlCl_3$ (30)	ND
14	$BiCl_3$ (30)	ND
15	$CoCl_2$ (30)	ND
16	$SnCl_2$ (30)	ND
17	$AuCl_3$ (30)	ND
18	No	61
19	AgOAc (10)	41
20	AgOAc (20)	54
21	AgOAc (40)	58

[a]Reaction conditions: **1a** (0.1 mmol), **2a** (0.3 mmol), $Pd(OTf)_2$ (30 mol%), catalysts (mol%), $K_2S_2O_8$ (2 equiv), CH_3CN (2 mL) as solvent, 100 °C, sealed tube for 1 h, [b] Isolated yields. [c] ND means not detected.

Table S3. Optimization of Oxidants[a, b]

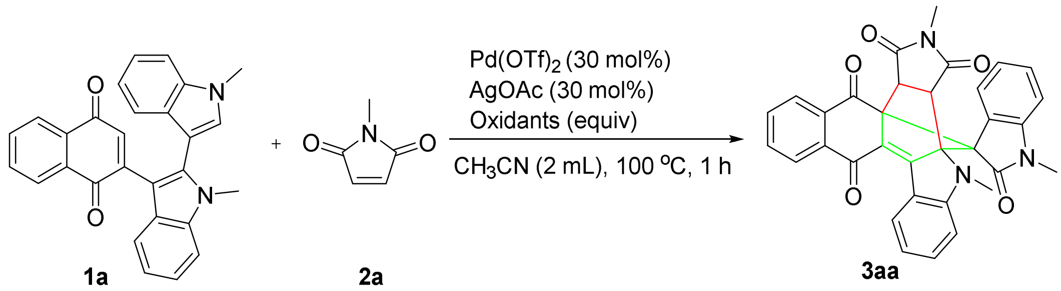

Entry	Oxidants (equiv)	Yield (%)
1	$K_2S_2O_8$ (2)	73
2	$(NH_4)_2S_2O_8$ (2)	57
3	Oxone (2)	ND[d]
4	KIO_3 (2)	ND
5	H_2O_2 (2)	NR[c]

6	TBHP (2)	NR
7	No	NR
8	$K_2S_2O_8$ (1)	60
9	$K_2S_2O_8$ (3)	65
10	$K_2S_2O_8$ (4)	68

[a]Reaction conditions: **1a** (0.1 mmol), **2a** (0.3 mmol), $Pd(OTf)_2$ (30 mol%), AgOAc (30 mol%), oxidants (equiv), CH_3CN (2 mL) as solvent, 100 °C, sealed tube for 1h, [b]Isolated yields. [c]NR means not reaction. [d] ND means not detected.

Table S4. Optimization of Solvents[a, b]

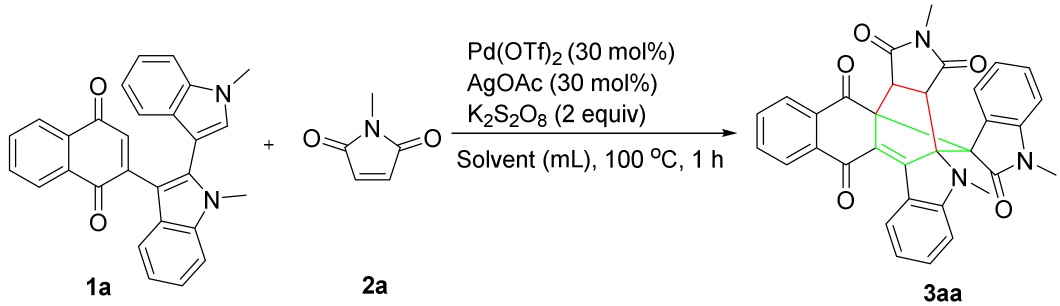

Entry	Solvent（mL）	Yield (%)
1	CH_3CN（2）	73
2	DCM（2）	Trace
3	DCE（2）	Trace
4	Toluene（2）	ND[c]
5	THF（2）	NR[d]
6	1,4-Dioxane（2）	ND
7	Bromobenzene（2）	NR
8	DMSO（2）	10
9	DMF（2）	Trace
10	CH_3CN（1）	55
11	CH_3CN（3）	50

[a]Reaction conditions: **1a** (0.1 mmol), **2a** (0.3 mmol), $Pd(OTf)_2$ (30 mol%), AgOAc (30 mol%), $K_2S_2O_8$ (2 equiv), solvent (mL), 100 °C, sealed tube for 1h, [b]Isolated yields.[c]NR means not reaction. [d] ND means not detected.

Table S5. Optimization of Additive[a, b]

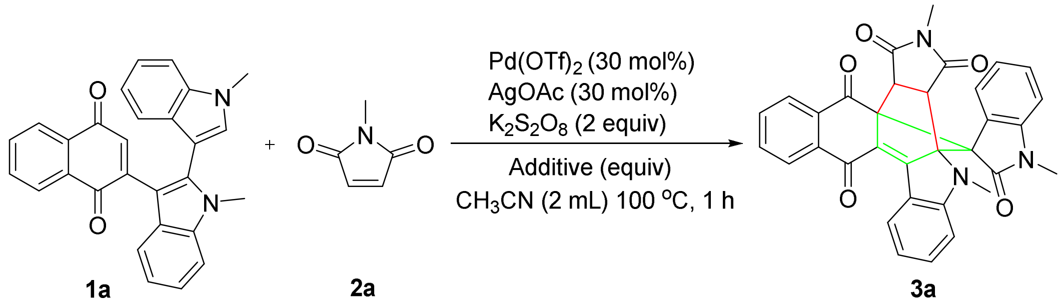

Entry	Additive (equiv)	Yield (%)
1	HOAc（1）	76
2	HCOOH（1）	Trace
3	PhCOOH（1）	35
4	$PhB(OH)_2$（1）	Trace
5	KOAc（1）	ND[c]
6	NaOAc（1）	37
7	NaOH（1）	ND
8	KOH（1）	20
9	Cs_2CO_3（1）	ND
10	K_2CO_3（1）	18
11	Na_2CO_3（1）	28
12	$NaHCO_3$（1）	30
13	No	73
14	HOAc（2）	77
15	HOAc（3）	82
16	HOAc（4）	80

[a] Reaction conditions: **1a** (0.1 mmol), **2a** (0.3 mmol) $Pd(OTf)_2$ (30 mol%), $K_2S_2O_8$ (2 equiv), AgOAc (30 mol%), additive (equiv), CH_3CN (2 mL) as solvent, 100 °C sealed tube for 1h. [b] Isolated yields.[c] ND means not detected.

Table S6. Optimization of dosages、temperature and time[a, b]

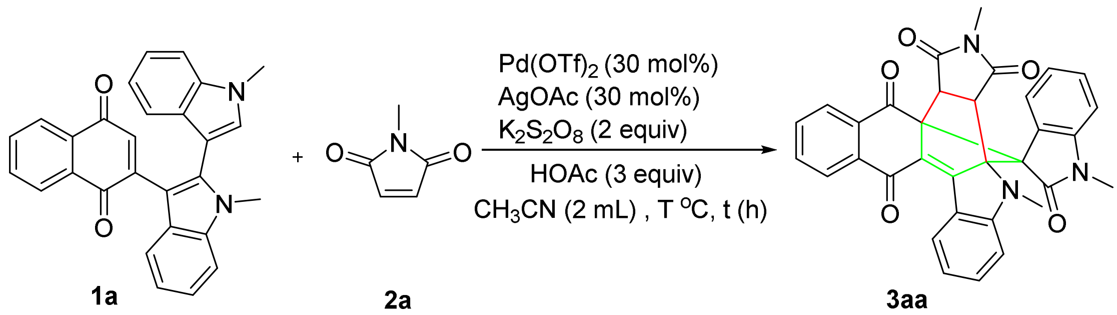

Entry	**2a** (equiv）	Temp (°C)	Time (h)	Yield (%)

1	1	100	1	53
2	2	100	1	70
3	3	100	1	82
4	4	100	1	80
5	3	80	1	61
6	3	110	1	53
7	3	120	1	48
8	3	100	0.5	65
9	3	100	1.5	79
10	3	100	2	68

[a]Reaction conditions: **1a** (0.1 mmol), **2a** (mmol), $Pd(OTf)_2$ (30 mol%), AgOAc (30 mol%), $K_2S_2O_8$ (2 equiv), HOAc (3 equiv), CH_3CN (2 mL) as solvent, T °C, sealed tube for time, [b]Isolated yields.

4 DFT calculation

Performed DFT calculation helped to estimate quantum chemical reactivity parameters including electronic structure and reactivity of the synthesized compounds. FMO for the synthesized compounds (3aa, 3av, 3ax;3az;4a, 4i) are represented in Figure S1. According to the FMO theory, reactions occurs when interaction of electrons from highest occupied molecular orbital (HOMO) of one compound with the electrons from lowest unoccupied molecular orbital (LUMO) of another compound takes place. The HOMO energy represents the electron donating potential of compound, while the LUMO energy represents the electron accepting potential of compound. Results of current DFT investigation indicated that compound 3az exerted strong HOMO energy while compound 4i showed lowest values for HOMO energy. The order of HOMO energy from low to high was found to be as 3az>3ax>4a>3av>3aa>4i. Similarly, compound 3az showed highest values for LUMO energy while compound 3aa showed minimum value for LUMO energy and the order of LUMO energy from low to was fond to be as 3az>4i>3ax>4a>3av>3aa. The highest HOMO-LUMO gaps (HLG) was exerted by compound indicating the energy required to excitation of an electron from the HOMO orbital to the LUMO orbital.

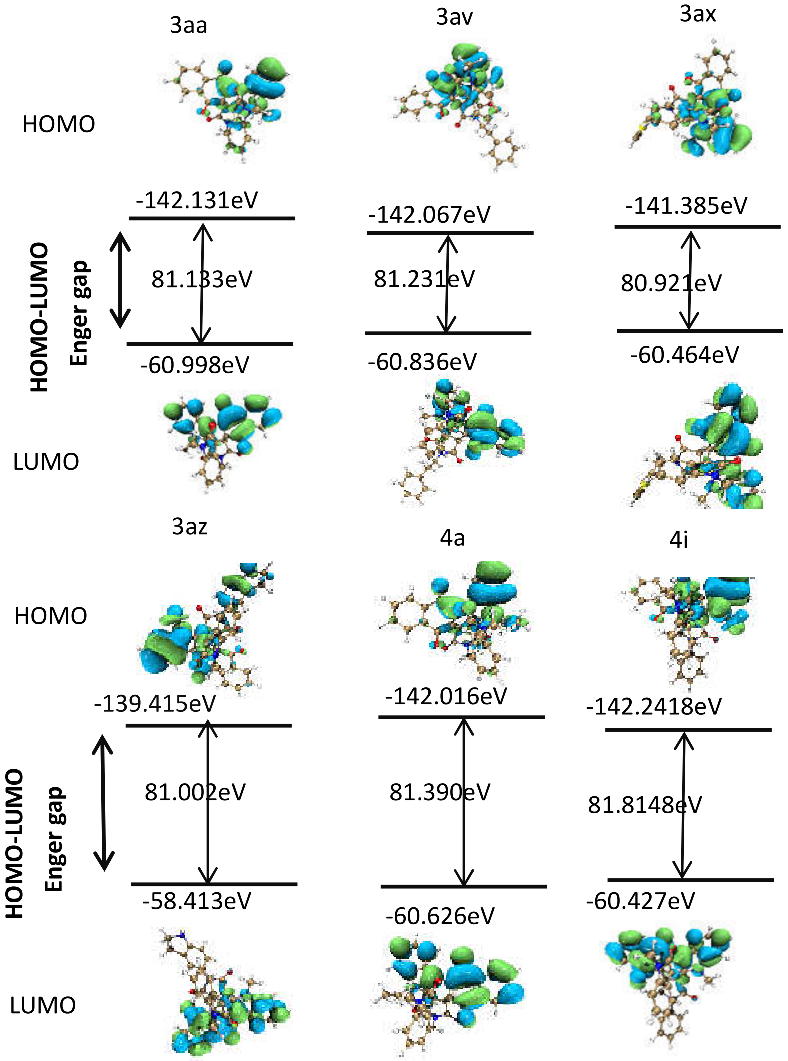

Figure S1. Frontier molecular orbitals including HOMO, LUMO and HOMO-LUMO energy gaps calculated via DFT method.

5 Experimental Section for Photo-Physical Studies

Table S7. Photophysical characterization data of all compounds

compound	λ_{max}(nm)	λ_{em}(nm)	Φ_F
**3aa**	502	586	0.43
**3ab**	503	611	0.59
**3ac**	503	607	0.26
**3ad**	503	607	0.25
**3ae**	505	613	0.37
**3af**	503	606	0.19
**3ag**	503	643	0.24
**3ah**	503	608	0.17
**3ai**	504	638	0.32
**3aj**	503	636	0.26
**3ak**	503	616	0.18
**3al**	501	661	0.34
**3am**	502	661	0.25
**3an**	505	641	0.24
**3ao**	503	615	0.28
**3ap**	501	592	0.14
**3aq**	503	609	0.19
**3ar**	502	608	0.16
**3as**	505	620	0.49
**3au**	503	662	0.23
**3av**	503	607	0.13
**3aw**	503	608	0.12
**3ax**	501	659	0.22
**3ay**	503	613	0.24
**3az**	501	585	0.83
**4a**	519	637	0.17
**4b**	517	648	0.41
**4c**	511	602	0.14
**4d**	511	618	0.16
**4e**	499	570	0.17
**4f**	497	570	0.14
**4g**	500	601	0.21
**4h**	501	649	0.19
**4i**	502	650	0.25
**4j**	515	582	0.19
**4k**	505	611	0.35
**4l**	499	565	0.10
**4m**	490	611	0.16
**4n**	497	599	0.27

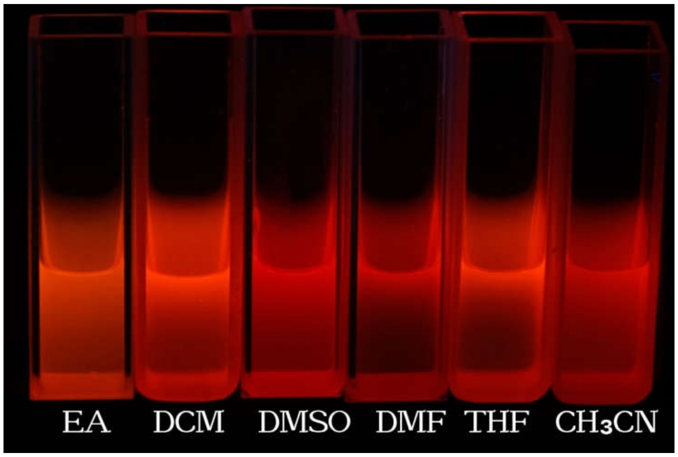

Figure S2. Compounds (C=5x10^{-4}M) in different solvents under 365nm UV,

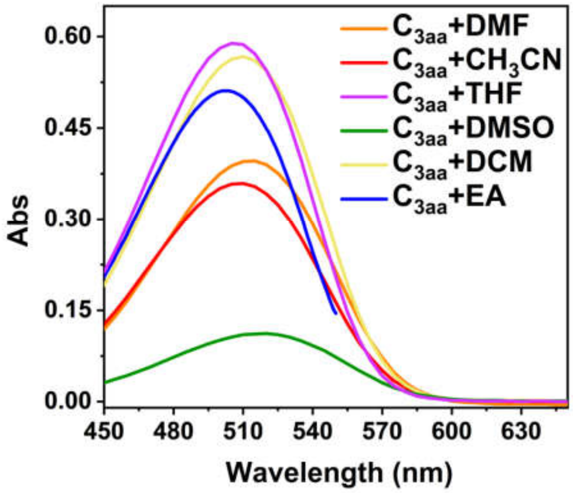

Figure S3. The UV/VIS spectrum of compound **3aa** (C=5x10^{-5}M) in different solvents

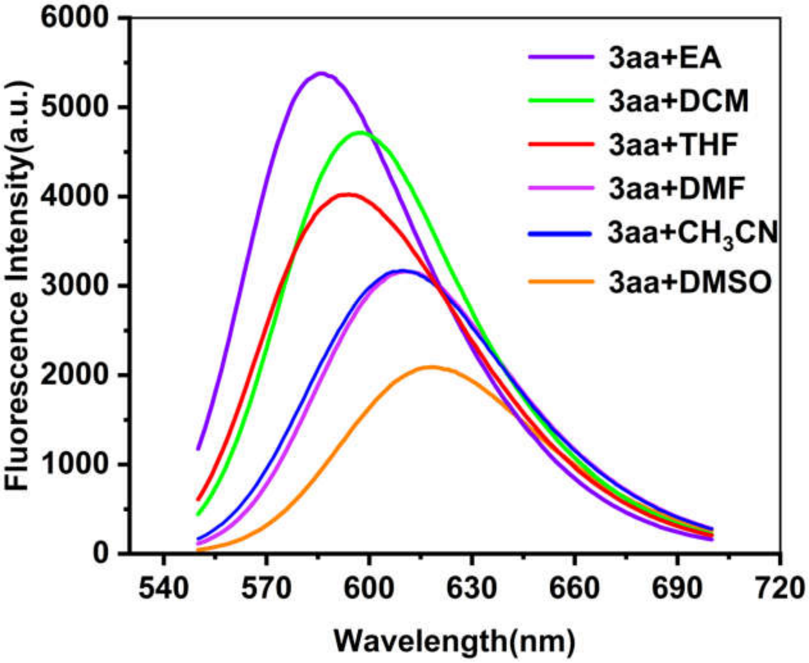

Figure S4. The fluorescence emission spectrum of **3aa**(C=5x10^{-5}M) in different solvents

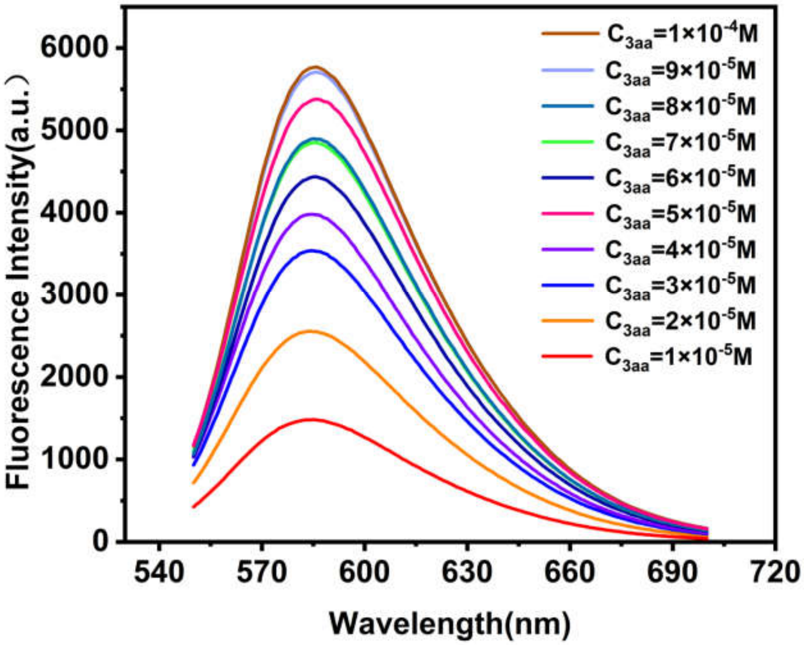

Figure S5. The fluorescence emission spectrum of gradient concentration of **3aa** in **EA**

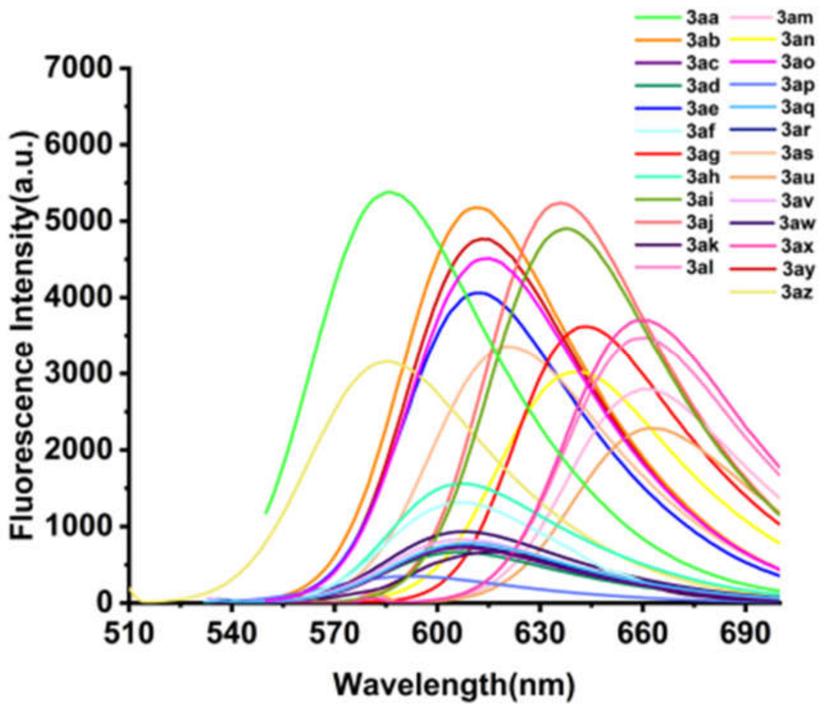

Figure S6. The fluorescence emission spectrum of compounds **3** (C=5x10^{-5}M) in **EA**

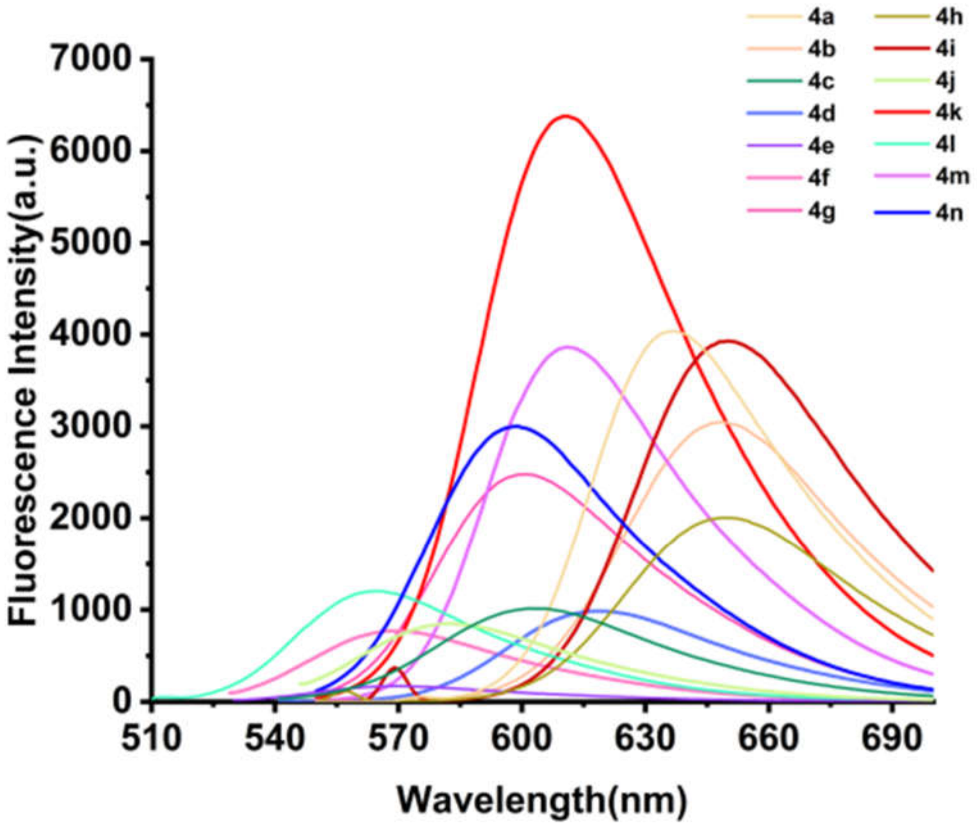

Figure S7. The fluorescence emission spectrum of compounds **4** (C=5x10^{-5}M) in **EA**

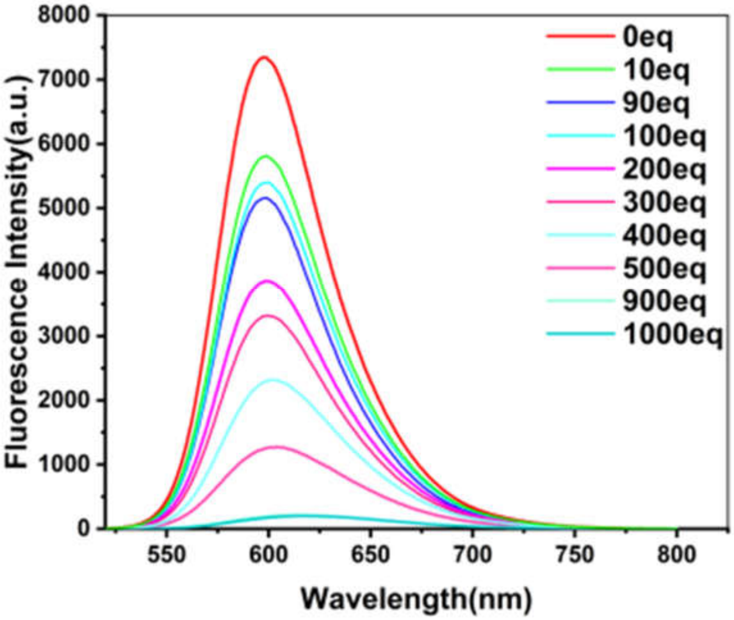

Figure S8. The fluorescence emission spectrum of **3aa**(C=5x10^{-5}M) with the increasing concentrations of **TFA** (0-1000eq)

6 Crystal data and structure refinement of product 5ae

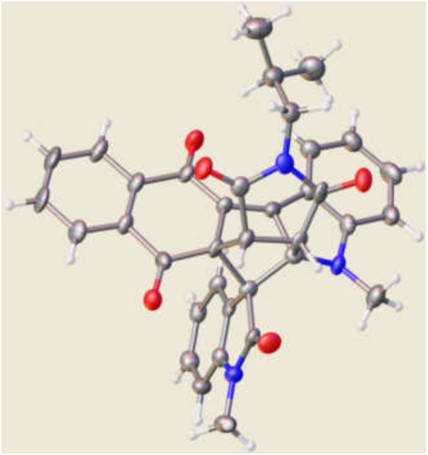

Figure S9. Crystal data and structure refinement of product **5ae** (CCDC：2328768)

Table S8. Crystal data and structure refinement for 5ae.

Identification code	5ae
Empirical formula	$C_{37}H_{30}Cl_3N_3O_5$
Formula weight	702.99

Temperature/K	150.00(10)
Crystal system	orthorhombic
Space group	$Pca2_1$
a/Å	22.3191(12)
b/Å	9.7227(5)
c/Å	15.3351(8)
α/°	90
β/°	90
γ/°	90
Volume/Å^3	3327.7(3)
Z	4
ρ_{calc}g/cm^3	1.403
μ/mm^{-1}	2.897
F(000)	1456.0
Crystal size/mm^3	0.16 × 0.14 × 0.11
Radiation	Cu Kα (λ = 1.54184)
2Θ range for data collection/°	7.922 to 133.176
Index ranges	$-25 \leq h \leq 26, -8 \leq k \leq 11, -18 \leq l \leq 16$
Reflections collected	10748
Independent reflections	4722 [R_{int} = 0.0907, R_{sigma} = 0.0749]
Data/restraints/parameters	4722/1/456
Goodness-of-fit on F^2	1.056
Final R indexes [I>=2σ (I)]	R_1 = 0.1027, wR_2 = 0.2724
Final R indexes [all data]	R_1 = 0.1113, wR_2 = 0.2837
Largest diff. peak/hole / e Å^{-3}	1.24/-0.46
Flack parameter	0.05(3)

Crystal structure determination of 5ae

Crystal Data for $C_{37}H_{30}Cl_3N_3O_5$ (*M* =702.99 g/mol): orthorhombic, space group $Pca2_1$ (no. 29), *a* = 22.3191(12) Å, *b* = 9.7227(5) Å, *c* = 15.3351(8) Å, *V* = 3327.7(3) Å^3, *Z* = 4, *T* = 150.00(10) K, μ(Cu Kα) = 2.897 mm^{-1}, *Dcalc* = 1.403 g/cm^3, 10748 reflections measured (7.922° ≤ 2Θ ≤ 133.176°), 4722 unique (R_{int} = 0.0907, R_{sigma} = 0.0749) which were used in all calculations. The final R_1 was 0.1027 (I > 2σ(I)) and wR_2 was 0.2837 (all data).

Refinement model description

Table S9. Fractional Atomic Coordinates (×10^4) and Equivalent Isotropic Displacement Parameters (Å^2×10^3) for 5ae. U_{eq} is defined as 1/3 of the trace of the orthogonalised U_{IJ} tensor.

Atom	*x*	*y*	*z*	U(eq)
Cl1	8862.9(7)	5228.2(18)	6485.2(13)	62.9(4)
Cl2	10127(2)	5774(8)	6469(4)	101(2)

Table S9. Fractional Atomic Coordinates (×10^4) and Equivalent Isotropic Displacement Parameters (Å^2×10^3) for 5ae. U_{eq} is defined as 1/3 of the trace of the orthogonalised U_{IJ} tensor.

Atom	*x*	*y*	*z*	U(eq)
Cl02	9454.9(16)	2984(3)	7477(2)	73.1(9)
Cl3	9647(3)	3240(6)	7103(7)	144(3)
Cl4	10150.7(13)	4696(5)	6374(2)	81.8(11)
C37	9529(3)	4806(8)	7044(5)	64(2)
O1	5790.0(16)	5703(4)	3886(3)	39.6(10)
O2	7634.5(17)	6465(4)	6127(3)	44.3(10)
O3	7544.6(18)	4752(3)	3355(3)	39.1(9)
O4	8273.1(18)	9067(4)	2899(3)	42.0(10)
O5	5995.4(17)	8526(4)	2582(2)	41.6(10)
N1	5389.3(18)	8969(4)	3742(3)	33.4(10)
N2	7129.8(19)	10550(4)	4015(3)	31.8(10)
N3	8034.7(18)	6777(5)	3115(3)	34.1(11)
C1	6897(2)	4941(5)	5557(4)	33.5(12)
C2	7026(3)	3939(5)	6168(4)	38.9(14)
C3	6695(3)	2754(5)	6178(5)	52.9(17)
C4	6221(3)	2554(5)	5610(5)	50.7(17)
C5	6087(3)	3519(5)	4997(4)	40.7(14)
C6	6422(2)	4731(4)	4958(3)	29.2(11)
C7	6254(2)	5772(5)	4286(4)	35.0(13)
C8	6672(2)	6958(4)	4155(3)	26.1(10)
C9	7267(2)	6213(4)	5540(4)	29.8(11)
C10	7149(2)	7169(4)	4849(3)	25.9(10)
C11	7361.0(19)	8482(5)	4721(3)	28.4(11)
C12	7019(2)	9099(5)	3948(3)	29.0(11)
C13	6386(2)	8439(5)	4097(3)	29.6(11)
C14	5919(2)	8648(5)	3373(4)	32.5(12)
C15	6035(2)	8849(4)	4900(3)	28.5(11)
C16	6182(2)	8936(5)	5787(3)	28.4(11)
C17	5731(2)	9300(5)	6371(3)	31.3(12)
C18	5169(2)	9559(5)	6130(4)	37.5(13)
C19	5006(2)	9500(5)	5227(4)	35.5(13)
C20	5454(2)	9113(5)	4660(3)	29.8(11)
C21	4824(2)	9073(6)	3293(4)	44.1(15)
C22	7021(3)	11475(6)	3311(4)	43.8(15)
C23	7591(2)	10735(5)	4610(3)	31.4(12)
C24	7909(2)	11963(5)	4784(4)	37.8(14)

Table S9. Fractional Atomic Coordinates (×10^4) and Equivalent Isotropic Displacement Parameters (Å^2×10^3) for 5ae. U_{eq} is defined as 1/3 of the trace of the orthogonalised U_{IJ} tensor.

Atom	*x*	*y*	*z*	U(eq)
C25	8335(2)	11920(6)	5443(5)	48.6(16)
C26	8459(2)	10750(6)	5914(4)	40.4(14)
C27	8165(2)	9532(5)	5734(4)	34.9(12)
C28	7732(2)	9524(5)	5069(3)	28.8(11)
C29	6995(2)	6893(5)	3232(3)	30.0(11)
C30	7249(2)	8327(5)	3108(3)	29.3(11)
C31	7900(2)	8172(5)	3039(3)	31.0(12)
C32	7538(2)	5967(5)	3241(3)	31.3(12)
C33	8635(2)	6184(5)	3091(4)	37.4(13)
C34	8885(3)	5949(7)	4011(4)	47.6(16)
C35	9152(3)	7235(8)	4379(5)	61.2(19)
C36	9338(4)	4783(8)	4007(6)	69(2)

Table S10. Anisotropic Displacement Parameters (Å^2×10^3) for 5ae. The Anisotropic displacement factor exponent takes the form: $-2\pi^2[h^2a^{*2}U_{11}+2hka^*b^*U_{12}+\ldots]$.

Atom	U_{11}	U_{22}	U_{33}	U_{23}	U_{13}	U_{12}
Cl1	50.7(7)	79.3(9)	58.7(8)	-31.1(8)	-3.5(7)	2.7(7)
Cl2	41.7(19)	196(6)	66(3)	17(4)	8(2)	-20(3)
Cl02	93.7(19)	57.6(15)	68.0(19)	-17.4(14)	9.7(16)	-14.1(14)
Cl3	137(4)	59(3)	235(8)	-35(4)	-95(4)	25(3)
Cl4	52.0(14)	144(3)	49.6(16)	22.2(18)	8.7(13)	1.6(16)
C37	53(3)	92(5)	47(4)	-25(3)	12(3)	-8(3)
O1	44.7(18)	36.4(17)	38(2)	1.6(16)	-3.7(17)	-6.8(15)
O2	56(2)	41.4(19)	35(2)	12.0(17)	-13.5(17)	-7.2(15)
O3	57(2)	22.2(14)	38(2)	-3.0(15)	4.8(17)	1.5(14)
O4	54(2)	32.5(17)	39(2)	-1.4(16)	12.1(17)	-6.5(15)
O5	53.0(19)	54(2)	18.0(16)	-1.3(16)	-2.6(15)	6.5(17)
N1	31.3(18)	33.6(19)	35(2)	2.7(18)	-4.8(18)	0.8(15)
N2	43(2)	22.6(16)	30(2)	4.8(16)	1.3(18)	-2.2(15)
N3	43(2)	41(2)	18.4(19)	-2.1(17)	2.6(17)	-5.3(17)
C1	51(3)	17.6(18)	32(2)	-2.4(19)	2(2)	3.4(17)
C2	67(3)	21(2)	29(3)	3(2)	0(2)	6(2)
C3	73(4)	22(2)	64(4)	15(2)	6(3)	3(2)
C4	78(4)	15(2)	59(4)	8(2)	2(3)	-6(2)
C5	60(3)	20(2)	42(3)	-10(2)	3(2)	-3(2)

Table S10. Anisotropic Displacement Parameters (Å^2×10^3) for 5ae. The Anisotropic displacement factor exponent takes the form: $-2\pi^2[h^2a^{*2}U_{11}+2hka^*b^*U_{12}+\ldots]$.

Atom	U_{11}	U_{22}	U_{33}	U_{23}	U_{13}	U_{12}
C6	47(2)	21.8(19)	18(2)	0.0(17)	2.8(19)	1.1(17)
C7	31(2)	24(2)	50(3)	-3(2)	2(2)	-2.5(17)
C8	43(2)	20.4(19)	14.4(19)	3.0(17)	0.0(18)	0.9(16)
C9	40(2)	18.2(18)	31(2)	-0.1(19)	-2(2)	2.8(16)
C10	35(2)	23.7(19)	19(2)	-2.1(17)	-2.5(18)	1.3(16)
C11	28(2)	30(2)	27(2)	3(2)	0.8(19)	2.7(16)
C12	35(2)	30(2)	22(2)	-7.8(19)	3.6(19)	3.7(17)
C13	40(2)	30(2)	19(2)	-4.2(18)	-2.9(19)	3.6(18)
C14	43(2)	23.9(19)	31(2)	9.2(19)	-6(2)	-2.7(18)
C15	43(2)	12.9(17)	29(2)	-0.8(17)	2(2)	-2.3(16)
C16	40(2)	23.3(19)	22(2)	-5.1(18)	-4.3(19)	0.6(16)
C17	45(2)	24(2)	25(2)	-4.9(18)	6(2)	-1.0(18)
C18	42(3)	33(2)	38(3)	-9(2)	9(2)	-0.4(19)
C19	29(2)	40(2)	37(3)	3(2)	3(2)	4.8(19)
C20	35(2)	26(2)	28(2)	-5.9(19)	2(2)	-4.6(17)
C21	34(2)	50(3)	49(3)	1(3)	-8(2)	3(2)
C22	62(3)	28(2)	41(3)	0(2)	-3(3)	-1(2)
C23	42(2)	21.9(19)	31(3)	-9.5(19)	6(2)	-4.7(17)
C24	39(2)	25(2)	50(3)	-5(2)	3(2)	-3.2(18)
C25	42(3)	41(3)	63(4)	-22(3)	-1(3)	-10(2)
C26	37(2)	43(3)	41(3)	-9(2)	1(2)	-5(2)
C27	37(2)	38(2)	29(3)	-9(2)	0(2)	-0.1(19)
C28	32(2)	28(2)	26(2)	-5.4(19)	2.6(19)	-2.7(17)
C29	41(2)	23(2)	26(2)	0.2(19)	1(2)	1.0(17)
C30	51(3)	26(2)	10.7(19)	1.7(17)	-4.1(19)	-0.3(18)
C31	54(3)	26(2)	13(2)	4.5(18)	1.0(19)	-4.7(19)
C32	45(2)	32(2)	17(2)	-5.6(19)	-2(2)	-0.2(19)
C33	41(2)	39(2)	32(3)	-2(2)	4(2)	-1(2)
C34	45(3)	50(3)	48(3)	6(3)	1(3)	2(2)
C35	65(4)	65(4)	54(4)	-10(3)	-16(3)	-3(3)
C36	60(4)	84(5)	65(5)	-2(4)	-7(4)	20(3)

Table S11. Bond Lengths for 5ae.

Atom	Atom	Length/Å	Atom	Atom	Length/Å
Cl1	C37	1.763(8)	C8	C13	1.578(6)
Cl2	C37	1.856(9)	C8	C29	1.590(7)

Table S11. Bond Lengths for 5ae.

Atom	Atom	Length/Å	Atom	Atom	Length/Å
Cl02	C37	1.899(9)	C9	C10	1.434(7)
Cl3	C37	1.548(10)	C10	C11	1.376(6)
Cl4	C37	1.730(8)	C11	C12	1.532(7)
O1	C7	1.206(6)	C11	C28	1.413(6)
O2	C9	1.242(6)	C12	C13	1.569(7)
O3	C32	1.195(6)	C12	C30	1.576(7)
O4	C31	1.223(6)	C13	C14	1.535(7)
O5	C14	1.231(7)	C13	C15	1.513(7)
N1	C14	1.347(7)	C15	C16	1.402(7)
N1	C20	1.422(7)	C15	C20	1.372(7)
N1	C21	1.440(7)	C16	C17	1.394(7)
N2	C12	1.436(6)	C17	C18	1.332(7)
N2	C22	1.427(7)	C18	C19	1.432(8)
N2	C23	1.388(7)	C19	C20	1.379(7)
N3	C31	1.394(6)	C23	C24	1.414(7)
N3	C32	1.374(7)	C23	C28	1.407(7)
N3	C33	1.458(7)	C24	C25	1.387(9)
C1	C2	1.383(7)	C25	C26	1.376(9)
C1	C6	1.417(7)	C26	C27	1.382(7)
C1	C9	1.488(6)	C27	C28	1.405(7)
C2	C3	1.369(8)	C29	C30	1.517(6)
C3	C4	1.384(10)	C29	C32	1.510(7)
C4	C5	1.361(8)	C30	C31	1.465(7)
C5	C6	1.397(7)	C33	C34	1.535(9)
C6	C7	1.493(7)	C34	C35	1.495(10)
C7	C8	1.496(6)	C34	C36	1.519(10)
C8	C10	1.519(6)			

Table S12. Bond Angles for 5ae.

Atom	Atom	Atom	Angle/°	Atom	Atom	Atom	Angle/°
Cl1	C37	Cl2	104.9(5)	C11	C12	C30	106.5(4)
Cl1	C37	Cl02	108.3(4)	C13	C12	C30	102.6(4)
Cl3	C37	Cl1	113.7(5)	C12	C13	C8	90.9(3)
Cl3	C37	Cl2	113.8(6)	C14	C13	C8	115.9(4)
Cl4	C37	Cl1	113.7(5)	C14	C13	C12	116.9(4)
Cl4	C37	Cl02	102.7(4)	C15	C13	C8	113.8(4)
C14	N1	C20	110.4(4)	C15	C13	C12	118.5(4)

Table S12. Bond Angles for 5ae.

Atom	Atom	Atom	Angle/°	Atom	Atom	Atom	Angle/°
C14	N1	C21	125.8(5)	C15	C13	C14	101.7(4)
C20	N1	C21	123.7(4)	O5	C14	N1	123.9(5)
C22	N2	C12	122.4(4)	O5	C14	C13	127.3(5)
C23	N2	C12	107.6(4)	N1	C14	C13	108.8(4)
C23	N2	C22	122.8(4)	C16	C15	C13	133.2(4)
C31	N3	C33	125.5(4)	C20	C15	C13	108.6(4)
C32	N3	C31	113.3(4)	C20	C15	C16	118.1(5)
C32	N3	C33	121.2(4)	C17	C16	C15	118.0(4)
C2	C1	C6	119.6(4)	C18	C17	C16	123.3(5)
C2	C1	C9	118.8(5)	C17	C18	C19	120.0(5)
C6	C1	C9	121.6(4)	C20	C19	C18	115.9(5)
C3	C2	C1	119.2(6)	C15	C20	N1	110.1(4)
C2	C3	C4	121.6(6)	C15	C20	C19	124.6(5)
C5	C4	C3	120.4(5)	C19	C20	N1	125.3(4)
C4	C5	C6	119.5(5)	N2	C23	C24	127.3(5)
C1	C6	C7	122.5(4)	N2	C23	C28	112.7(4)
C5	C6	C1	119.6(5)	C28	C23	C24	120.0(5)
C5	C6	C7	117.9(5)	C25	C24	C23	117.1(5)
O1	C7	C6	121.9(4)	C26	C25	C24	123.1(5)
O1	C7	C8	120.7(5)	C25	C26	C27	120.5(5)
C6	C7	C8	117.3(4)	C26	C27	C28	118.4(5)
C7	C8	C10	116.5(4)	C23	C28	C11	106.3(4)
C7	C8	C13	117.3(4)	C27	C28	C11	132.9(5)
C7	C8	C29	111.8(4)	C27	C28	C23	120.8(4)
C10	C8	C13	101.6(3)	C30	C29	C8	104.2(4)
C10	C8	C29	108.2(4)	C32	C29	C8	112.3(4)
C13	C8	C29	99.8(3)	C32	C29	C30	104.4(4)
O2	C9	C1	121.2(5)	C29	C30	C12	102.3(4)
O2	C9	C10	122.0(4)	C31	C30	C12	115.5(4)
C10	C9	C1	116.7(4)	C31	C30	C29	106.6(4)
C9	C10	C8	124.0(4)	O4	C31	N3	124.1(5)
C11	C10	C8	105.4(4)	O4	C31	C30	127.9(5)
C11	C10	C9	130.1(4)	N3	C31	C30	107.9(4)
C10	C11	C12	107.6(4)	O3	C32	N3	125.2(5)
C10	C11	C28	144.4(5)	O3	C32	C29	127.0(5)
C28	C11	C12	107.7(4)	N3	C32	C29	107.8(4)
N2	C12	C11	104.0(4)	N3	C33	C34	111.7(5)
N2	C12	C13	123.1(4)	C35	C34	C33	111.5(5)

Table S12. Bond Angles for 5ae.

Atom	Atom	Atom	Angle/°	Atom	Atom	Atom	Angle/°
N2	C12	C30	118.1(4)	C35	C34	C36	111.2(6)
C11	C12	C13	100.2(4)	C36	C34	C33	110.5(6)

Table S13. Torsion Angles for 5ae.

A	B	C	D	Angle/°	A	B	C	D	Angle/°
O1	C7	C8	C10	163.5(5)	C12	C13	C14	N1	-136.4(4)
O1	C7	C8	C13	42.9(7)	C12	C13	C15	C16	-48.9(7)
O1	C7	C8	C29	-71.4(6)	C12	C13	C15	C20	134.5(4)
O2	C9	C10	C8	-175.3(5)	C12	C30	C31	O4	-70.4(6)
O2	C9	C10	C11	-4.5(8)	C12	C30	C31	N3	112.7(4)
N2	C12	C13	C8	-166.3(4)	C13	C8	C10	C9	137.2(4)
N2	C12	C13	C14	73.9(6)	C13	C8	C10	C11	-35.5(5)
N2	C12	C13	C15	-48.4(6)	C13	C8	C29	C30	39.7(4)
N2	C12	C30	C29	-174.3(4)	C13	C8	C29	C32	152.0(4)
N2	C12	C30	C31	70.4(6)	C13	C12	C30	C29	-35.4(4)
N2	C23	C24	C25	176.7(5)	C13	C12	C30	C31	-150.8(4)
N2	C23	C28	C11	3.0(6)	C13	C15	C16	C17	-177.0(5)
N2	C23	C28	C27	-176.3(5)	C13	C15	C20	N1	-2.5(5)
N3	C33	C34	C35	-82.6(6)	C13	C15	C20	C19	179.2(4)
N3	C33	C34	C36	153.2(5)	C14	N1	C20	C15	-1.5(5)
C1	C2	C3	C4	-2.1(9)	C14	N1	C20	C19	176.8(5)
C1	C6	C7	O1	-166.5(5)	C14	C13	C15	C16	-178.5(5)
C1	C6	C7	C8	10.5(7)	C14	C13	C15	C20	4.9(5)
C1	C9	C10	C8	0.6(7)	C15	C13	C14	O5	175.5(5)
C1	C9	C10	C11	171.4(5)	C15	C13	C14	N1	-5.8(5)
C2	C1	C6	C5	0.7(8)	C15	C16	C17	C18	0.4(7)
C2	C1	C6	C7	179.5(5)	C16	C15	C20	N1	-179.7(4)
C2	C1	C9	O2	-9.2(8)	C16	C15	C20	C19	2.0(7)
C2	C1	C9	C10	174.9(5)	C16	C17	C18	C19	-1.4(8)
C2	C3	C4	C5	2.6(10)	C17	C18	C19	C20	2.5(7)
C3	C4	C5	C6	-1.4(9)	C18	C19	C20	N1	179.1(4)
C4	C5	C6	C1	-0.2(8)	C18	C19	C20	C15	-2.9(7)
C4	C5	C6	C7	-179.1(5)	C20	N1	C14	O5	-176.5(5)
C5	C6	C7	O1	12.4(8)	C20	N1	C14	C13	4.7(5)
C5	C6	C7	C8	-170.7(5)	C20	C15	C16	C17	-0.7(6)
C6	C1	C2	C3	0.5(8)	C21	N1	C14	O5	7.1(8)
C6	C1	C9	O2	171.5(5)	C21	N1	C14	C13	-171.7(4)

Table S13. Torsion Angles for 5ae.

A	B	C	D	Angle/°	A	B	C	D	Angle/°
C6	C1	C9	C10	-4.4(7)	C21	N1	C20	C15	175.0(4)
C6	C7	C8	C10	-13.5(6)	C21	N1	C20	C19	-6.7(7)
C6	C7	C8	C13	-134.1(4)	C22	N2	C12	C11	164.0(5)
C6	C7	C8	C29	111.6(5)	C22	N2	C12	C13	-83.7(6)
C7	C8	C10	C9	8.5(7)	C22	N2	C12	C30	46.2(6)
C7	C8	C10	C11	-164.2(4)	C22	N2	C23	C24	18.9(8)
C7	C8	C13	C12	-178.6(4)	C22	N2	C23	C28	-161.5(5)
C7	C8	C13	C14	-58.0(6)	C23	N2	C12	C11	12.9(5)
C7	C8	C13	C15	59.5(6)	C23	N2	C12	C13	125.3(5)
C7	C8	C29	C30	164.4(4)	C23	N2	C12	C30	-104.8(5)
C7	C8	C29	C32	-83.2(5)	C23	C24	C25	C26	0.7(9)
C8	C10	C11	C12	0.0(5)	C24	C23	C28	C11	-177.3(5)
C8	C10	C11	C28	171.8(6)	C24	C23	C28	C27	3.4(7)
C8	C13	C14	O5	-60.5(7)	C24	C25	C26	C27	1.1(9)
C8	C13	C14	N1	118.2(4)	C25	C26	C27	C28	-0.7(8)
C8	C13	C15	C16	56.1(7)	C26	C27	C28	C11	179.4(5)
C8	C13	C15	C20	-120.5(4)	C26	C27	C28	C23	-1.5(8)
C8	C29	C30	C12	-2.8(5)	C28	C11	C12	N2	-11.4(5)
C8	C29	C30	C31	118.8(4)	C28	C11	C12	C13	-139.5(4)
C8	C29	C32	O3	65.0(7)	C28	C11	C12	C30	114.1(4)
C8	C29	C32	N3	-113.6(4)	C28	C23	C24	C25	-2.9(8)
C9	C1	C2	C3	-178.8(5)	C29	C8	C10	C9	-118.4(5)
C9	C1	C6	C5	180.0(5)	C29	C8	C10	C11	68.9(4)
C9	C1	C6	C7	-1.2(8)	C29	C8	C13	C12	-57.8(4)
C9	C10	C11	C12	-172.1(5)	C29	C8	C13	C14	62.8(5)
C9	C10	C11	C28	-0.3(11)	C29	C8	C13	C15	-179.7(4)
C10	C8	C13	C12	53.2(4)	C29	C30	C31	O4	176.7(5)
C10	C8	C13	C14	173.8(4)	C29	C30	C31	N3	-0.1(5)
C10	C8	C13	C15	-68.7(5)	C30	C12	C13	C8	57.6(4)
C10	C8	C29	C30	-66.0(4)	C30	C12	C13	C14	-62.2(5)
C10	C8	C29	C32	46.3(5)	C30	C12	C13	C15	175.5(4)
C10	C11	C12	N2	163.6(4)	C30	C29	C32	O3	177.3(5)
C10	C11	C12	C13	35.5(5)	C30	C29	C32	N3	-1.4(5)
C10	C11	C12	C30	-71.0(5)	C31	N3	C32	O3	-177.3(5)
C10	C11	C28	C23	-166.4(6)	C31	N3	C32	C29	1.4(6)
C10	C11	C28	C27	12.7(11)	C31	N3	C33	C34	96.5(6)
C11	C12	C13	C8	-52.0(4)	C32	N3	C31	O4	-177.8(5)
C11	C12	C13	C14	-171.8(4)	C32	N3	C31	C30	-0.8(6)

Table S13. Torsion Angles for 5ae.

A	B	C	D	Angle/°	A	B	C	D	Angle/°
C11	C12	C13	C15	65.9(5)	C32	N3	C33	C34	-81.9(6)
C11	C12	C30	C29	69.3(4)	C32	C29	C30	C12	-120.8(4)
C11	C12	C30	C31	-46.0(5)	C32	C29	C30	C31	0.9(5)
C12	N2	C23	C24	169.7(5)	C33	N3	C31	O4	3.7(8)
C12	N2	C23	C28	-10.6(6)	C33	N3	C31	C30	-179.3(4)
C12	C11	C28	C23	5.4(5)	C33	N3	C32	O3	1.3(8)
C12	C11	C28	C27	-175.5(5)	C33	N3	C32	C29	180.0(4)
C12	C13	C14	O5	44.9(7)					

Table S14. Hydrogen Atom Coordinates (Å×10^4) and Isotropic Displacement Parameters (Å^2×10^3) for 5ae.

Atom	*x*	*y*	*z*	U(eq)
H37A	9602.31	5467.15	7531.36	77
H37	9497.54	5174.54	7650.59	77
H2	7341.49	4070.76	6575.77	47
H3	6793.03	2051.79	6584.95	64
H4	5988.66	1738.34	5647.68	61
H5	5766.95	3369.84	4599.76	49
H16	6577.71	8751.94	5984.18	34
H17	5828.99	9365.87	6972.82	38
H18	4875.87	9783.41	6556.22	45
H19	4612.64	9714.23	5031.5	43
H21A	4515.92	8581.89	3624.62	66
H21B	4861.12	8663.72	2711.41	66
H21C	4711.77	10043.01	3237.79	66
H22A	7007.72	12419.38	3532.51	66
H22B	6637.47	11248.09	3035.35	66
H22C	7343.78	11391.61	2880.44	66
H24	7834.5	12781.64	4463.92	45
H25	8549.88	12736.28	5575.2	58
H26	8750.03	10779.72	6365.91	49
H27	8252.74	8719.44	6053.74	42
H29	6709.38	6640.75	2755.3	36
H30	7082.24	8761.74	2568.99	35
H33A	8905.1	6808.19	2767.44	45
H33B	8621.4	5295.6	2775.94	45
H34	8544.74	5669.94	4396.27	57

Table S14. Hydrogen Atom Coordinates (Å×10^4) and Isotropic Displacement Parameters (Å^2×10^3) for 5ae.

Atom	*x*	*y*	*z*	U(eq)
H35A	9553.94	7368.98	4137.37	92
H35B	9176.72	7157	5014.63	92
H35C	8898.49	8022.01	4224.5	92
H36A	9164.35	3979.3	3715.12	104
H36B	9442.99	4541.36	4608.21	104
H36C	9699.39	5074.56	3694.15	104

Table S15. Atomic Occupancy for 5ae.

Atom	*Occupancy*	Atom	*Occupancy*	Atom	*Occupancy*
Cl2	0.407(3)	Cl02	0.593(3)	Cl3	0.407(3)
Cl4	0.593(3)	H37A	0.593(3)	H37	0.407(3)

7 Crystal data and structure refinement of product 6i

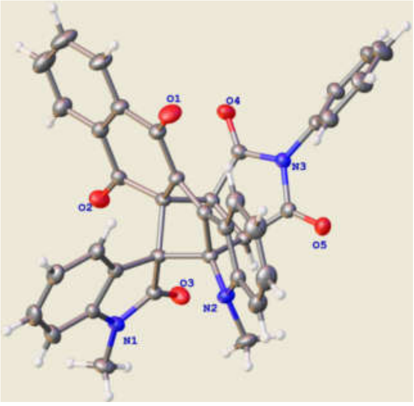

Figure S10. Crystal data and structure refinement of product **6i** (CCDC：2328767)

Table S16. Crystal data and structure refinement for 6i.

Identification code	189-1

Empirical formula	$C_{38}H_{25}N_3O_5$
Formula weight	603.61
Temperature/K	199.99(10)
Crystal system	monoclinic
Space group	$P2_1/c$
a/Å	11.1938(7)
b/Å	15.3426(9)
c/Å	17.2928(11)
α/°	90
β/°	102.276(6)
γ/°	90
Volume/Å^3	2902.0(3)
Z	4
ρ_{calc}g/cm^3	1.382
μ/mm^{-1}	0.754
F(000)	1256.0
Crystal size/mm^3	0.14 × 0.12 × 0.1
Radiation	Cu Kα (λ = 1.54184)
2Θ range for data collection/°	7.784 to 147.886
Index ranges	$-11 \leq h \leq 13$, $-18 \leq k \leq 17$, $-21 \leq l \leq 20$
Reflections collected	11530
Independent reflections	5724 [R_{int} = 0.0619, R_{sigma} = 0.0927]
Data/restraints/parameters	5724/0/417
Goodness-of-fit on F^2	1.033
Final R indexes [I>=2σ (I)]	R_1 = 0.0614, wR_2 = 0.1293
Final R indexes [all data]	R_1 = 0.0991, wR_2 = 0.1552
Largest diff. peak/hole / e Å^{-3}	0.34/-0.22

Crystal structure determination of 6i

Crystal Data for $C_{38}H_{25}N_3O_5$ (*M* =603.61 g/mol): monoclinic, space group $P2_1/c$ (no. 14), *a* = 11.1938(7) Å, *b* = 15.3426(9) Å, *c* = 17.2928(11) Å, *β* = 102.276(6)°, *V* = 2902.0(3) Å^3, *Z* = 4, *T* = 199.99(10) K, μ(Cu Kα) = 0.754 mm^{-1}, *Dcalc* = 1.382 g/cm^3, 11530 reflections measured (7.784° ≤ 2Θ ≤ 147.886°), 5724 unique (R_{int} = 0.0619, R_{sigma} = 0.0927) which were used in all calculations. The final R_1 was 0.0614 (I > 2σ(I)) and wR_2 was 0.1552 (all data).

Refinement model description

Table S17. Fractional Atomic Coordinates (×10^4) and Equivalent Isotropic Displacement Parameters (Å^2×10^3) for 6i. U_{eq} is defined as 1/3 of the trace of the orthogonalised U_{IJ} tensor.

Atom	*x*	*y*	*z*	U(eq)
O1	1982(2)	7993.5(14)	2225.1(13)	55.2(6)

Table S17. Fractional Atomic Coordinates (×10[4]) and Equivalent Isotropic Displacement Parameters (Å[2]×10[3]) for 6i. U_{eq} is defined as 1/3 of the trace of the orthogonalised U_{IJ} tensor.

Atom	*x*	*y*	*z*	U(eq)
O2	1639(2)	4849.3(13)	3591.9(13)	49.7(6)
O3	3557(2)	5223.6(14)	5378.4(12)	46.5(5)
O4	4525(2)	5797.6(13)	2630.2(12)	44.9(5)
O5	6106.0(18)	7717.9(13)	4605.6(12)	43.5(5)
N1	1672(2)	5655.6(15)	5531.3(13)	36.3(5)
N2	3540(2)	7754.3(15)	5317.6(13)	37.3(5)
N3	5502(2)	6837.8(15)	3506.3(13)	34.5(5)
C1	1384(3)	6506(2)	2066.8(17)	41.9(7)
C2	846(4)	6602(2)	1268.5(19)	57.7(9)
C3	212(4)	5929(3)	848(2)	72.6(12)
C4	69(4)	5150(3)	1201(2)	68.5(11)
C5	596(3)	5030(2)	1987(2)	52.1(8)
C6	1264(3)	5690.8(18)	2426.8(17)	38.4(6)
C7	1834(3)	5523.8(17)	3263.3(17)	35.0(6)
C8	2661(2)	6197.4(17)	3712.5(15)	30.5(5)
C9	2575(2)	7102.8(17)	3344.1(15)	31.9(6)
C10	2003(3)	7266.7(19)	2523.4(16)	37.9(6)
C11	3032(2)	7665.6(17)	3935.9(15)	31.6(5)
C12	3425(2)	7136.8(17)	4686.7(15)	31.4(5)
C13	2418(2)	6417.1(17)	4556.6(15)	30.6(5)
C14	2660(2)	5679.1(17)	5184.4(16)	33.2(6)
C15	1145(2)	6715.6(17)	4589.6(15)	33.2(6)
C16	355(3)	7325(2)	4159.0(17)	40.4(6)
C17	-793(3)	7454(2)	4346(2)	48.1(8)
C18	-1131(3)	6988(2)	4950(2)	52.1(8)
C19	-354(3)	6374(2)	5375(2)	47.3(7)
C20	770(2)	6240.0(18)	5183.7(15)	32.3(6)
C21	1617(3)	5104(2)	6199.1(18)	49.0(8)
C22	4054(2)	5942.5(17)	3935.4(15)	32.6(6)
C23	4580(2)	6592.6(18)	4590.2(15)	33.2(6)
C24	5488(2)	7128.9(18)	4273.7(16)	33.6(6)
C25	4692(3)	6145.1(17)	3269.2(16)	33.5(6)
C26	6188(3)	7257.8(19)	3004.6(16)	37.1(6)
C27	6085(3)	8149(2)	2899.2(19)	47.2(7)
C28	6755(4)	8552(2)	2416(2)	61.1(10)
C29	7528(4)	8068(3)	2052(2)	69.6(12)

Table S17. Fractional Atomic Coordinates (×10^4) and Equivalent Isotropic Displacement Parameters (Å^2×10^3) for 6i. U_{eq} is defined as 1/3 of the trace of the orthogonalised U_{IJ} tensor.

Atom	*x*	*y*	*z*	U(eq)
C30	7616(3)	7189(2)	2158(2)	56.8(9)
C31	6936(3)	6765(2)	2631.0(18)	44.3(7)
C32	3157(3)	8561.5(17)	4156.8(17)	36.2(6)
C33	3453(3)	8584.4(18)	5001.5(17)	38.0(6)
C34	3584(3)	9377(2)	5402(2)	51.9(8)
C35	3449(4)	10129(2)	4960(3)	64.0(10)
C36	3179(4)	10104(2)	4130(2)	60.7(9)
C37	3027(3)	9338.9(19)	3723(2)	49.0(8)
C38	4163(3)	7559(2)	6122.4(17)	52.0(8)

Table S18. Anisotropic Displacement Parameters (Å^2×10^3) for 6i. The Anisotropic displacement factor exponent takes the form: $-2\pi^2[h^2a^{*2}U_{11}+2hka^*b^*U_{12}+\ldots]$.

Atom	U_{11}	U_{22}	U_{33}	U_{23}	U_{13}	U_{12}
O1	75.9(16)	42.9(12)	40.9(12)	15.1(10)	-1.0(11)	-2.7(12)
O2	67.9(15)	39.1(11)	44.7(12)	1.8(10)	18.3(11)	-12.2(11)
O3	45.4(12)	50.4(12)	45.0(12)	18.7(10)	12.6(10)	12.7(10)
O4	52.9(12)	48.3(12)	36.9(11)	-4.7(9)	17.3(9)	-11.1(10)
O5	36.2(10)	49.5(12)	43.9(11)	-5.2(10)	7.0(9)	-3.6(10)
N1	39.3(12)	38.7(12)	33.5(12)	4.1(10)	13.7(10)	-1.3(10)
N2	40.6(13)	41.7(13)	28.5(11)	-3.5(10)	4.9(10)	1.1(11)
N3	34.1(11)	36.6(12)	33.9(12)	1.5(10)	9.4(10)	-2.8(10)
C1	44.3(16)	48.0(17)	31.6(14)	3.1(13)	4.3(12)	0.8(14)
C2	75(2)	57(2)	33.8(16)	4.8(16)	-4.9(16)	-0.6(19)
C3	100(3)	65(2)	38.3(19)	-3.9(18)	-18(2)	-2(2)
C4	78(3)	63(2)	53(2)	-11.3(19)	-10.6(19)	-10(2)
C5	57(2)	47.3(18)	48.3(18)	-3.9(15)	2.6(16)	-7.7(16)
C6	40.0(15)	38.6(15)	35.8(15)	-1.7(12)	6.2(12)	0.2(12)
C7	36.7(14)	34.3(14)	36.7(14)	-0.6(12)	14.3(12)	-1.6(12)
C8	31.9(13)	32.8(13)	26.7(12)	5.3(11)	5.9(10)	3.4(11)
C9	35.3(13)	32.2(13)	27.0(12)	4.7(11)	3.8(11)	0.5(11)
C10	41.7(15)	38.9(15)	31.5(14)	6.1(12)	4.3(12)	-0.1(12)
C11	31.0(13)	31.8(13)	32.1(13)	4.9(11)	7.5(11)	3.0(11)
C12	32.1(13)	33.5(13)	27.4(12)	4.1(11)	3.5(10)	3.4(11)
C13	32.0(13)	33.5(13)	26.6(12)	4.8(11)	6.7(10)	5.2(11)
C14	34.7(14)	34.0(13)	31.5(13)	4.4(11)	8.3(11)	2.0(12)

Table S18. Anisotropic Displacement Parameters (Å^2×10^3) for 6i. The Anisotropic displacement factor exponent takes the form: $-2\pi^2[h^2a^{*2}U_{11}+2hka^*b^*U_{12}+…]$.

Atom	U_{11}	U_{22}	U_{33}	U_{23}	U_{13}	U_{12}
C15	33.1(13)	35.9(14)	30.4(13)	-0.8(11)	6.3(11)	-0.6(11)
C16	33.2(14)	47.4(16)	38.8(15)	-1.9(13)	3.9(12)	4.5(13)
C17	33.9(15)	54.3(19)	52.9(18)	-5.0(16)	1.9(14)	10.7(14)
C18	30.8(14)	62(2)	65(2)	-12.5(17)	12.7(14)	1.9(15)
C19	39.6(15)	52.9(18)	53.8(19)	-0.3(15)	20.0(14)	-4.4(14)
C20	29.5(13)	36.7(13)	31.4(13)	-2.1(11)	8.3(11)	-2.0(11)
C21	65(2)	46.6(17)	40.2(16)	9.5(14)	21.0(16)	-2.9(16)
C22	34.9(14)	31.6(13)	32.9(14)	4.9(11)	11.0(11)	3.2(11)
C23	33.2(13)	37.9(14)	28.5(13)	5.1(11)	6.1(11)	3.2(11)
C24	30.0(13)	37.1(14)	32.4(13)	1.3(12)	4.0(11)	4.7(12)
C25	35.7(14)	32.8(13)	33.6(14)	2.9(11)	11.0(11)	1.8(11)
C26	36.6(14)	42.3(15)	32.7(14)	6.0(12)	7.7(11)	-5.4(12)
C27	51.7(18)	44.9(17)	46.9(18)	5.2(14)	14.8(15)	-7.1(15)
C28	80(3)	44.3(18)	64(2)	10.3(17)	27(2)	-17.1(18)
C29	85(3)	70(2)	64(2)	-2(2)	38(2)	-36(2)
C30	56(2)	72(2)	49.3(19)	-8.1(18)	25.2(16)	-16.6(18)
C31	43.6(16)	51.0(18)	41.3(16)	4.7(14)	15.8(13)	-2.2(14)
C32	37.5(14)	30.1(13)	39.8(15)	0.3(12)	5.2(12)	1.8(12)
C33	32.6(13)	38.1(15)	43.6(16)	-4.5(13)	8.7(12)	-1.0(12)
C34	52.0(18)	50.5(18)	52.1(19)	-13.5(16)	8.2(15)	-4.3(16)
C35	67(2)	37.0(18)	86(3)	-12.5(19)	11(2)	-5.1(17)
C36	72(2)	34.3(16)	73(2)	4.6(17)	9(2)	0.3(16)
C37	50.6(18)	37.7(15)	56(2)	8.8(15)	5.7(15)	-1.5(14)
C38	61(2)	63(2)	28.8(15)	-3.2(15)	2.1(14)	0.2(17)

Table S19. Bond Lengths for 6i.

Atom	Atom	Length/Å	Atom	Atom	Length/Å
O1	C10	1.227(3)	C11	C12	1.515(3)
O2	C7	1.222(3)	C11	C32	1.426(4)
O3	C14	1.211(3)	C12	C13	1.560(4)
O4	C25	1.205(3)	C12	C23	1.577(4)
O5	C24	1.206(3)	C13	C14	1.552(3)
N1	C14	1.366(3)	C13	C15	1.510(4)
N1	C20	1.388(4)	C15	C16	1.389(4)
N1	C21	1.444(3)	C15	C20	1.395(4)
N2	C12	1.430(3)	C16	C17	1.404(4)

Table S19. Bond Lengths for 6i.

Atom	Atom	Length/Å	Atom	Atom	Length/Å
N2	C33	1.381(4)	C17	C18	1.383(5)
N2	C38	1.449(4)	C18	C19	1.383(5)
N3	C24	1.403(3)	C19	C20	1.382(4)
N3	C25	1.401(4)	C22	C23	1.530(4)
N3	C26	1.429(3)	C22	C25	1.511(3)
C1	C2	1.392(4)	C23	C24	1.499(4)
C1	C6	1.416(4)	C26	C27	1.382(4)
C1	C10	1.494(4)	C26	C31	1.385(4)
C2	C3	1.370(5)	C27	C28	1.381(4)
C3	C4	1.367(5)	C28	C29	1.389(5)
C4	C5	1.374(5)	C29	C30	1.361(5)
C5	C6	1.387(4)	C30	C31	1.392(4)
C6	C7	1.474(4)	C32	C33	1.428(4)
C7	C8	1.491(4)	C32	C37	1.400(4)
C8	C9	1.523(3)	C33	C34	1.392(4)
C8	C13	1.577(3)	C34	C35	1.375(5)
C8	C22	1.573(4)	C35	C36	1.403(5)
C9	C10	1.449(4)	C36	C37	1.360(5)
C9	C11	1.354(4)			

Table S20. Bond Angles for 6i.

Atom	Atom	Atom	Angle/°	Atom	Atom	Atom	Angle/°
C14	N1	C20	112.0(2)	C15	C13	C8	116.7(2)
C14	N1	C21	122.9(3)	C15	C13	C12	116.0(2)
C20	N1	C21	125.1(2)	C15	C13	C14	102.7(2)
C12	N2	C38	122.9(2)	O3	C14	N1	124.4(2)
C33	N2	C12	108.8(2)	O3	C14	C13	128.7(2)
C33	N2	C38	123.3(3)	N1	C14	C13	106.8(2)
C24	N3	C26	123.1(2)	C16	C15	C13	133.0(2)
C25	N3	C24	112.4(2)	C16	C15	C20	119.3(3)
C25	N3	C26	124.2(2)	C20	C15	C13	107.6(2)
C2	C1	C6	117.9(3)	C15	C16	C17	118.8(3)
C2	C1	C10	119.7(3)	C18	C17	C16	120.8(3)
C6	C1	C10	122.3(3)	C17	C18	C19	120.7(3)
C3	C2	C1	120.8(3)	C20	C19	C18	118.4(3)
C4	C3	C2	121.2(3)	N1	C20	C15	110.6(2)
C3	C4	C5	119.5(4)	C19	C20	N1	127.4(3)

Table S20. Bond Angles for 6i.

Atom	Atom	Atom	Angle/°	Atom	Atom	Atom	Angle/°
C4	C5	C6	120.8(3)	C19	C20	C15	122.0(3)
C1	C6	C7	121.6(3)	C23	C22	C8	103.0(2)
C5	C6	C1	119.7(3)	C25	C22	C8	111.5(2)
C5	C6	C7	118.8(3)	C25	C22	C23	105.1(2)
O2	C7	C6	121.4(3)	C22	C23	C12	103.2(2)
O2	C7	C8	119.8(3)	C24	C23	C12	112.5(2)
C6	C7	C8	118.8(2)	C24	C23	C22	105.8(2)
C7	C8	C9	115.9(2)	O5	C24	N3	124.6(3)
C7	C8	C13	114.8(2)	O5	C24	C23	127.0(3)
C7	C8	C22	115.4(2)	N3	C24	C23	108.4(2)
C9	C8	C13	100.6(2)	O4	C25	N3	124.6(2)
C9	C8	C22	107.5(2)	O4	C25	C22	127.0(3)
C22	C8	C13	100.6(2)	N3	C25	C22	108.3(2)
C10	C9	C8	122.9(2)	C27	C26	N3	119.1(3)
C11	C9	C8	106.6(2)	C27	C26	C31	121.4(3)
C11	C9	C10	130.3(3)	C31	C26	N3	119.6(3)
O1	C10	C1	121.5(3)	C28	C27	C26	118.8(3)
O1	C10	C9	122.3(3)	C27	C28	C29	120.4(3)
C9	C10	C1	116.1(2)	C30	C29	C28	120.2(3)
C9	C11	C12	107.5(2)	C29	C30	C31	120.6(3)
C9	C11	C32	144.8(3)	C26	C31	C30	118.6(3)
C32	C11	C12	107.0(2)	C11	C32	C33	106.6(2)
N2	C12	C11	105.2(2)	C37	C32	C11	133.2(3)
N2	C12	C13	121.6(2)	C37	C32	C33	120.1(3)
N2	C12	C23	119.3(2)	N2	C33	C32	111.3(2)
C11	C12	C13	100.7(2)	N2	C33	C34	128.1(3)
C11	C12	C23	106.7(2)	C34	C33	C32	120.5(3)
C13	C12	C23	101.1(2)	C35	C34	C33	117.9(3)
C12	C13	C8	91.34(19)	C34	C35	C36	121.4(3)
C14	C13	C8	116.9(2)	C37	C36	C35	121.9(3)
C14	C13	C12	113.7(2)	C36	C37	C32	118.1(3)

Table S21. Torsion Angles for 6i.

A	B	C	D	Angle/°	A	B	C	D	Angle/°
O2	C7	C8	C9	-163.4(3)	C12	C23	C24	O5	67.9(4)
O2	C7	C8	C13	-46.6(4)	C12	C23	C24	N3	-111.5(2)
O2	C7	C8	C22	69.7(3)	C13	C8	C9	C10	-141.1(3)

Table S21. Torsion Angles for 6i.

A	B	C	D	Angle/˚	A	B	C	D	Angle/˚
N2	C12	C13	C8	167.2(2)	C13	C8	C9	C11	34.4(3)
N2	C12	C13	C14	-72.4(3)	C13	C8	C22	C23	-38.4(2)
N2	C12	C13	C15	46.4(3)	C13	C8	C22	C25	-150.6(2)
N2	C12	C23	C22	173.0(2)	C13	C12	C23	C22	36.8(2)
N2	C12	C23	C24	-73.4(3)	C13	C12	C23	C24	150.3(2)
N2	C33	C34	C35	-179.5(3)	C13	C15	C16	C17	-179.6(3)
N3	C26	C27	C28	179.8(3)	C13	C15	C20	N1	-0.7(3)
N3	C26	C31	C30	-178.7(3)	C13	C15	C20	C19	178.6(3)
C1	C2	C3	C4	1.3(7)	C14	N1	C20	C15	-2.1(3)
C1	C6	C7	O2	173.3(3)	C14	N1	C20	C19	178.6(3)
C1	C6	C7	C8	-6.6(4)	C14	C13	C15	C16	-176.5(3)
C2	C1	C6	C5	-1.8(5)	C14	C13	C15	C20	2.9(3)
C2	C1	C6	C7	178.3(3)	C15	C13	C14	O3	179.3(3)
C2	C1	C10	O1	4.8(5)	C15	C13	C14	N1	-4.1(3)
C2	C1	C10	C9	-178.0(3)	C15	C16	C17	C18	0.4(5)
C2	C3	C4	C5	-1.8(7)	C16	C15	C20	N1	178.7(2)
C3	C4	C5	C6	0.5(6)	C16	C15	C20	C19	-2.0(4)
C4	C5	C6	C1	1.3(5)	C16	C17	C18	C19	-1.1(5)
C4	C5	C6	C7	-178.7(3)	C17	C18	C19	C20	0.3(5)
C5	C6	C7	O2	-6.6(4)	C18	C19	C20	N1	-179.6(3)
C5	C6	C7	C8	173.5(3)	C18	C19	C20	C15	1.2(5)
C6	C1	C2	C3	0.5(6)	C20	N1	C14	O3	-179.2(3)
C6	C1	C10	O1	-171.9(3)	C20	N1	C14	C13	3.9(3)
C6	C1	C10	C9	5.4(4)	C20	C15	C16	C17	1.1(4)
C6	C7	C8	C9	16.5(4)	C21	N1	C14	O3	2.9(5)
C6	C7	C8	C13	133.3(2)	C21	N1	C14	C13	-174.0(2)
C6	C7	C8	C22	-110.4(3)	C21	N1	C20	C15	175.7(3)
C7	C8	C9	C10	-16.6(4)	C21	N1	C20	C19	-3.6(5)
C7	C8	C9	C11	158.9(2)	C22	C8	C9	C10	114.2(3)
C7	C8	C13	C12	-176.8(2)	C22	C8	C9	C11	-70.3(3)
C7	C8	C13	C14	65.5(3)	C22	C8	C13	C12	58.6(2)
C7	C8	C13	C15	-56.6(3)	C22	C8	C13	C14	-59.0(3)
C7	C8	C22	C23	-162.6(2)	C22	C8	C13	C15	178.9(2)
C7	C8	C22	C25	85.3(3)	C22	C23	C24	O5	179.8(3)
C8	C9	C10	O1	-177.0(3)	C22	C23	C24	N3	0.4(3)
C8	C9	C10	C1	5.8(4)	C23	C12	C13	C8	-57.8(2)
C8	C9	C11	C12	0.4(3)	C23	C12	C13	C14	62.5(3)
C8	C9	C11	C32	-168.2(4)	C23	C12	C13	C15	-178.7(2)

Table S21. Torsion Angles for 6i.

A	B	C	D	Angle/˚	A	B	C	D	Angle/˚
C8	C13	C14	O3	50.1(4)	C23	C22	C25	O4	-178.1(3)
C8	C13	C14	N1	-133.2(2)	C23	C22	C25	N3	-0.1(3)
C8	C13	C15	C16	-47.2(4)	C24	N3	C25	O4	178.5(3)
C8	C13	C15	C20	132.2(2)	C24	N3	C25	C22	0.3(3)
C8	C22	C23	C12	1.3(2)	C24	N3	C26	C27	-49.8(4)
C8	C22	C23	C24	-117.1(2)	C24	N3	C26	C31	130.6(3)
C8	C22	C25	O4	-67.2(4)	C25	N3	C24	O5	-179.9(3)
C8	C22	C25	N3	110.8(2)	C25	N3	C24	C23	-0.5(3)
C9	C8	C13	C12	-51.6(2)	C25	N3	C26	C27	124.4(3)
C9	C8	C13	C14	-169.2(2)	C25	N3	C26	C31	-55.2(4)
C9	C8	C13	C15	68.6(3)	C25	C22	C23	C12	118.1(2)
C9	C8	C22	C23	66.4(2)	C25	C22	C23	C24	-0.2(3)
C9	C8	C22	C25	-45.8(3)	C26	N3	C24	O5	-5.1(4)
C9	C11	C12	N2	-162.7(2)	C26	N3	C24	C23	174.3(2)
C9	C11	C12	C13	-35.6(3)	C26	N3	C25	O4	3.7(4)
C9	C11	C12	C23	69.6(3)	C26	N3	C25	C22	-174.4(2)
C9	C11	C32	C33	161.6(4)	C26	C27	C28	C29	-0.8(6)
C9	C11	C32	C37	-17.2(7)	C27	C26	C31	C30	1.7(5)
C10	C1	C2	C3	-176.2(4)	C27	C28	C29	C30	1.2(7)
C10	C1	C6	C5	174.9(3)	C28	C29	C30	C31	-0.1(6)
C10	C1	C6	C7	-5.1(4)	C29	C30	C31	C26	-1.3(5)
C10	C9	C11	C12	175.5(3)	C31	C26	C27	C28	-0.6(5)
C10	C9	C11	C32	6.9(6)	C32	C11	C12	N2	10.4(3)
C11	C9	C10	O1	8.6(5)	C32	C11	C12	C13	137.6(2)
C11	C9	C10	C1	-168.6(3)	C32	C11	C12	C23	-117.2(2)
C11	C12	C13	C8	51.8(2)	C32	C33	C34	C35	-1.6(5)
C11	C12	C13	C14	172.1(2)	C33	N2	C12	C11	-9.9(3)
C11	C12	C13	C15	-69.0(3)	C33	N2	C12	C13	-123.0(3)
C11	C12	C23	C22	-68.1(2)	C33	N2	C12	C23	109.7(3)
C11	C12	C23	C24	45.4(3)	C33	C32	C37	C36	-0.7(5)
C11	C32	C33	N2	1.0(3)	C33	C34	C35	C36	0.5(6)
C11	C32	C33	C34	-177.2(3)	C34	C35	C36	C37	0.5(6)
C11	C32	C37	C36	177.9(3)	C35	C36	C37	C32	-0.4(6)
C12	N2	C33	C32	5.9(3)	C37	C32	C33	N2	180.0(3)
C12	N2	C33	C34	-176.0(3)	C37	C32	C33	C34	1.7(4)
C12	C11	C32	C33	-7.1(3)	C38	N2	C12	C11	-165.4(3)
C12	C11	C32	C37	174.2(3)	C38	N2	C12	C13	81.5(3)
C12	C13	C14	O3	-54.5(4)	C38	N2	C12	C23	-45.8(4)

Table S21. Torsion Angles for 6i.

A	B	C	D	Angle/°	A	B	C	D	Angle/°
C12	C13	C14	N1	122.2(2)	C38	N2	C33	C32	161.3(3)
C12	C13	C15	C16	58.8(4)	C38	N2	C33	C34	-20.7(5)
C12	C13	C15	C20	-121.9(2)					

Table S22. Hydrogen Atom Coordinates (Å×10^4) and Isotropic Displacement Parameters (Å^2×10^3) for 6i.

Atom	*x*	*y*	*z*	U(eq)
H2	918.44	7141.06	1011.73	69
H3	-133.36	6005.54	300.94	87
H4	-392.32	4695.45	905.48	82
H5	501.48	4487.09	2232.2	63
H16	586.7	7647.78	3745.79	48
H17	-1344.18	7866.04	4054.02	58
H18	-1905.21	7091.78	5074.57	63
H19	-586.7	6051.64	5788.13	57
H21A	1539.25	5465.87	6652.51	74
H21B	2366.17	4755.16	6335.9	74
H21C	908.72	4715.04	6062.13	74
H22	4178.94	5324.38	4118	39
H23	4968.63	6289.94	5092.97	40
H27	5563.69	8479.65	3154.61	57
H28	6685.62	9163.76	2332.11	73
H29	7997.4	8351.26	1728.32	83
H30	8146.12	6862.74	1906.42	68
H31	6983.86	6150.54	2696.43	53
H34	3760.74	9397.68	5964	62
H35	3541.12	10676.72	5221.34	77
H36	3099.16	10636.77	3843.32	73
H37	2838.84	9332.25	3160.87	59
H38A	3895.42	6988.94	6276.72	78
H38B	3967.21	8007.84	6479.38	78
H38C	5047.7	7548.89	6155.27	78

8 References

1. L. Zhang, C. Peng, D. Zhao, Y. Wang, H.-J. Fu, Q. Shen and J.-X. Li, Cu (ii)-catalyzed C–H (SP3) oxidation and C–N cleavage: base-switched methylenation and formylation using tetramethylethylenediamine as a carbon source, *Chem. Commun.*, 2012, **48**, 5928-5930.
2. S. Kirchberg, R. Fröhlich, A. Studer, Stereoselective Palladium - Catalyzed Carboaminoxylations of Indoles with Arylboronic Acids and TEMPO, *Angew. Chem. Int. Ed.*, 2009, **48**, 4235-4238.
3. R. Mandal, B. Emayavaramban and B. Sundararaju, Cp*Co(III)-Catalyzed C–H Alkylation with Maleimides Using Weakly Coordinating Carbonyl Directing Groups, *Org. Lett.*, 2018, **20**, 2835-2838.

9 Copies of ^{1}H, ^{13}C and ^{19}F NMR spectra for all compounds

Figure S11. :^{1}H NMR (400 MHz, CDCl$_3$) spectrum of 3aa

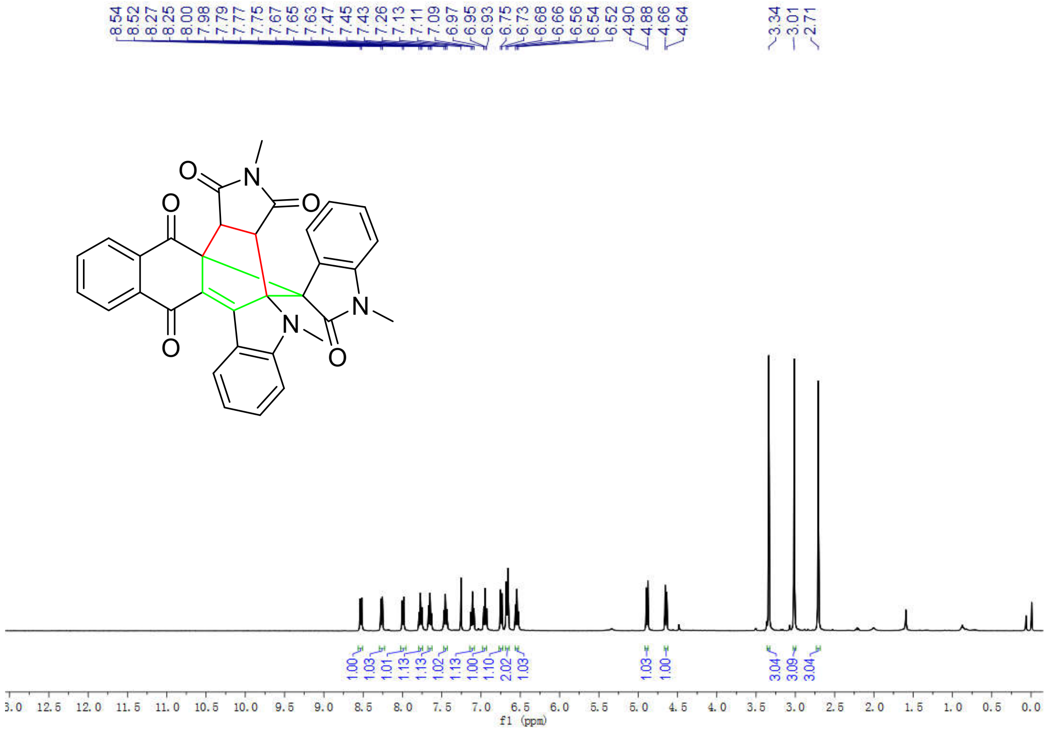

Figure S12. ^{13}C NMR (101 MHz, CDCl$_3$) spectrum of 3aa

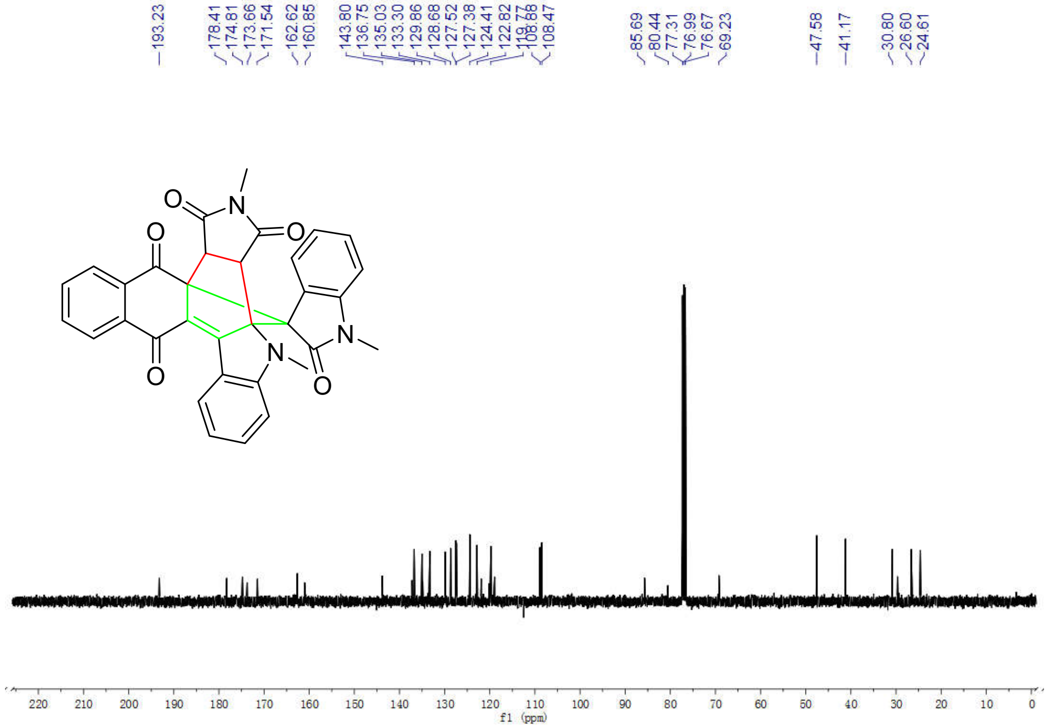

Figure S13. ^{1}H NMR (400 MHz, CDCl$_3$) spectrum of 3ab

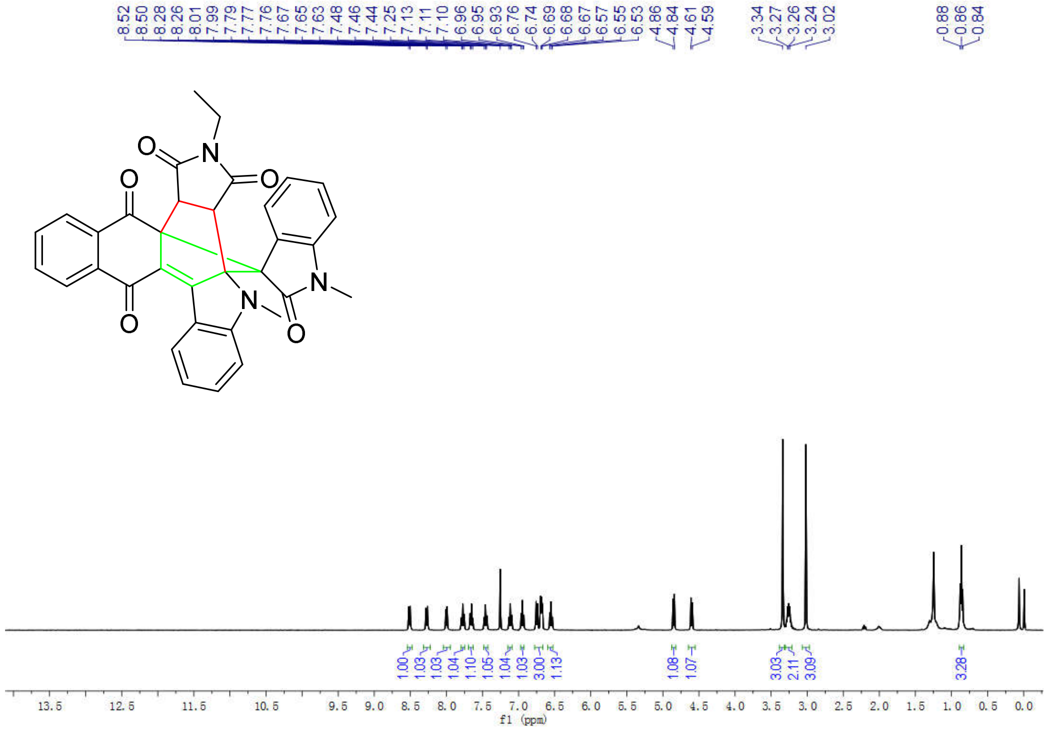

Figure S14. **^{13}C NMR (101 MHz, CDCl3) spectrum of 3ab**

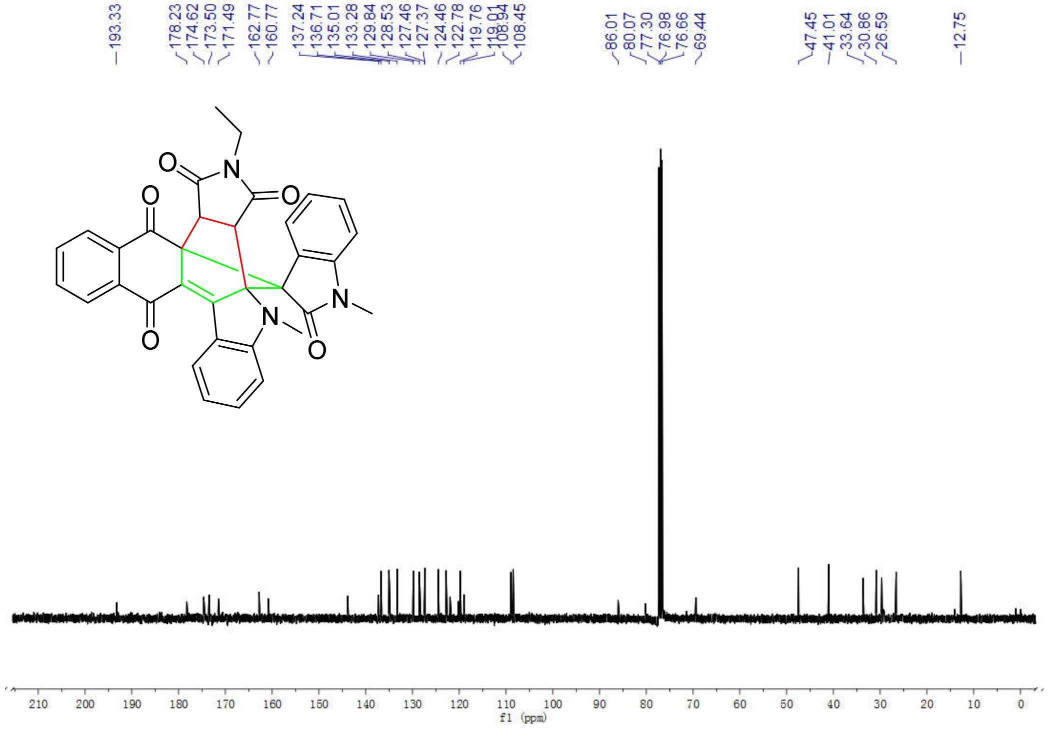

Figure S15. **^{1}H NMR (400 MHz, CDCl3) spectrum of 3ac**

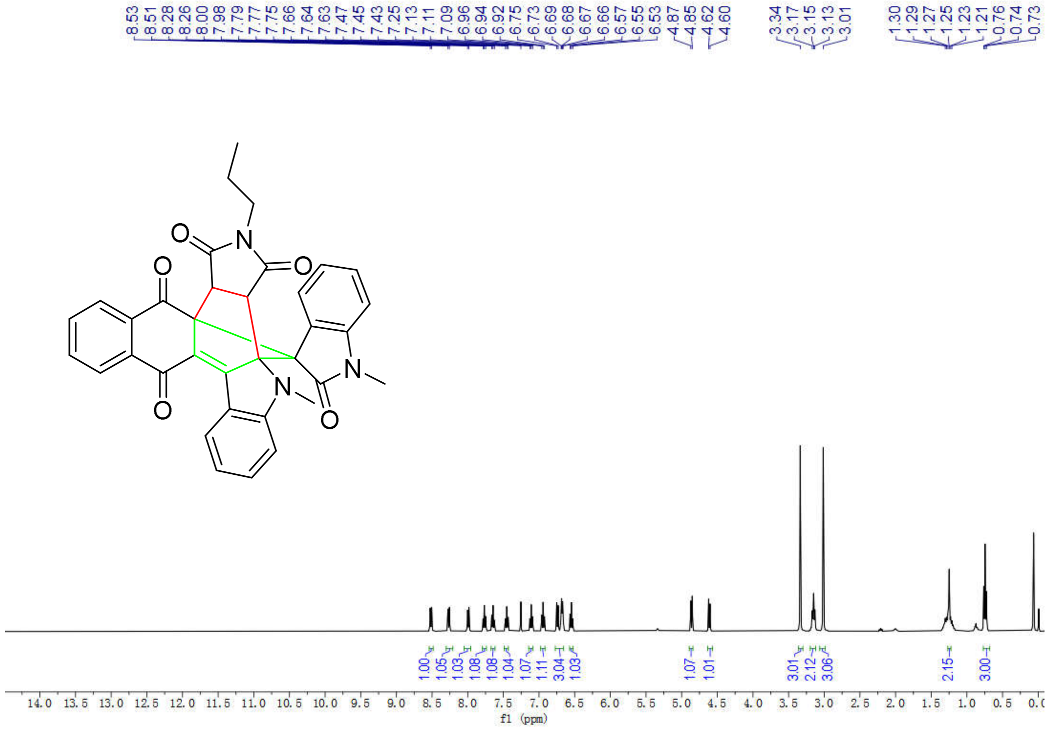

Figure S16. ^{13}C NMR (101 MHz, $CDCl_3$) spectrum of 3ac

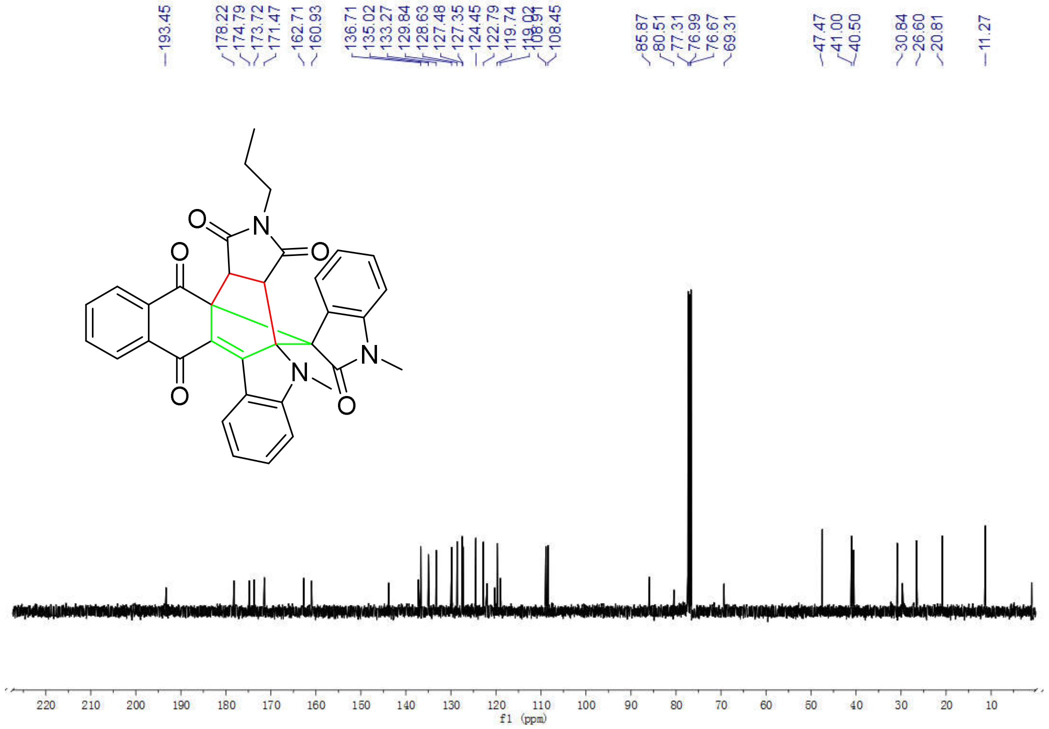

Figure S17. ^{1}H NMR (400 MHz, CDCl$_3$) spectrum of 3ad

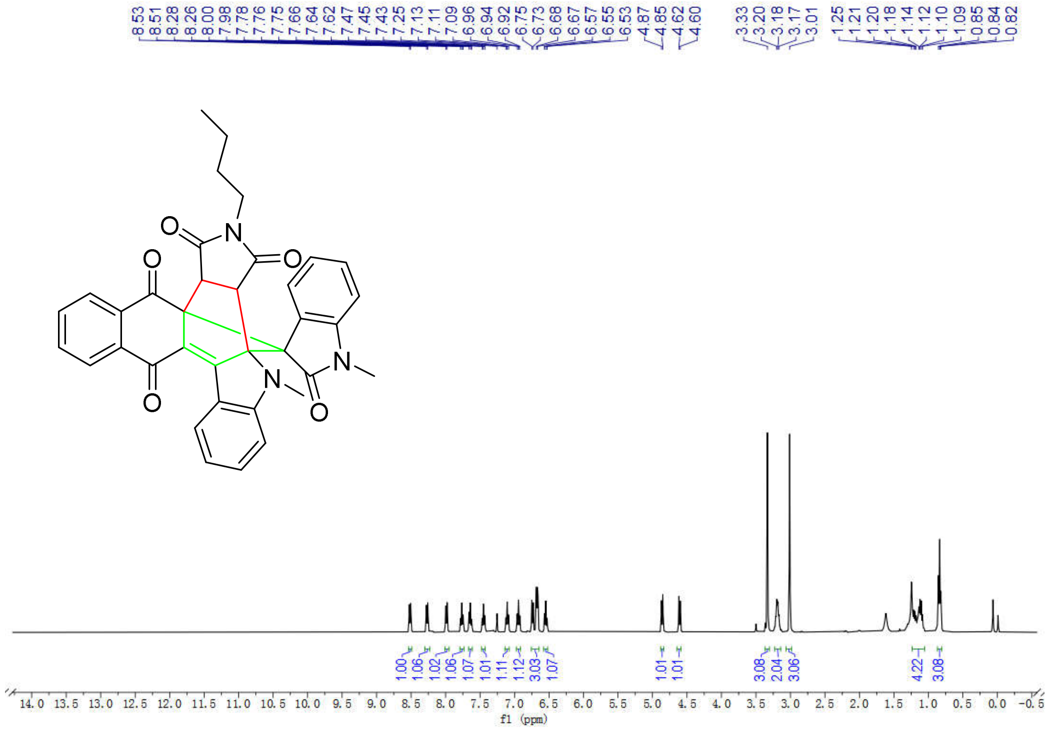

Figure S18. ^{13}C NMR (101 MHz, CDCl$_3$) spectrum of 3ad

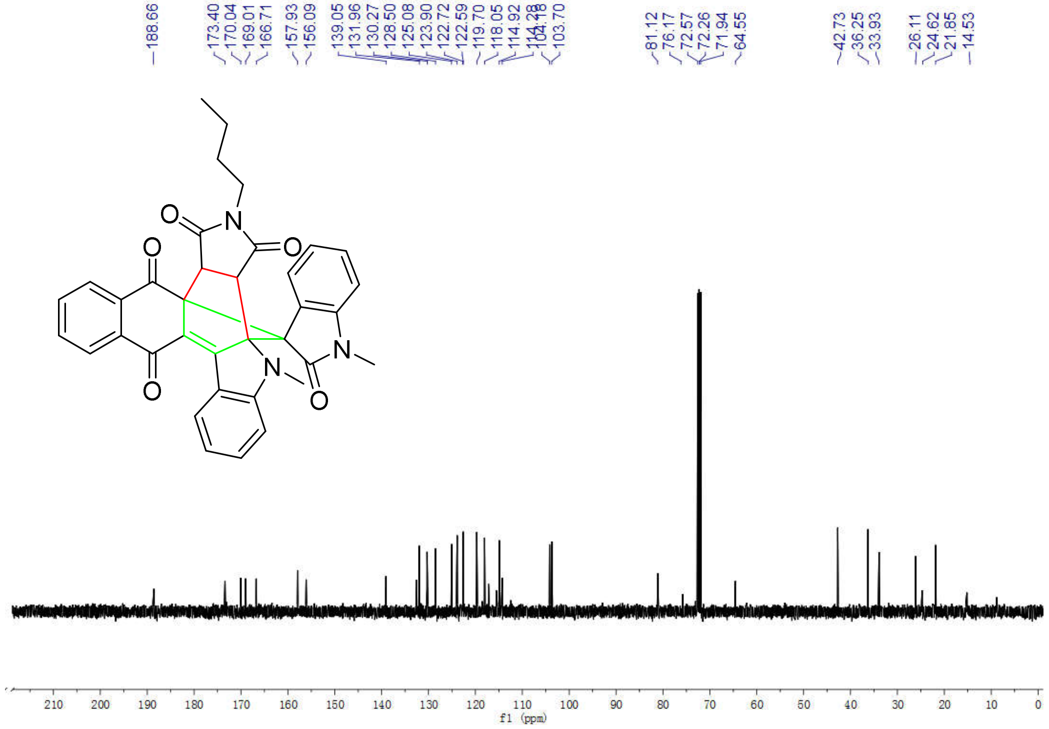

Figure S19. ^{1}H NMR (400 MHz, CDCl$_3$) spectrum of 3ae

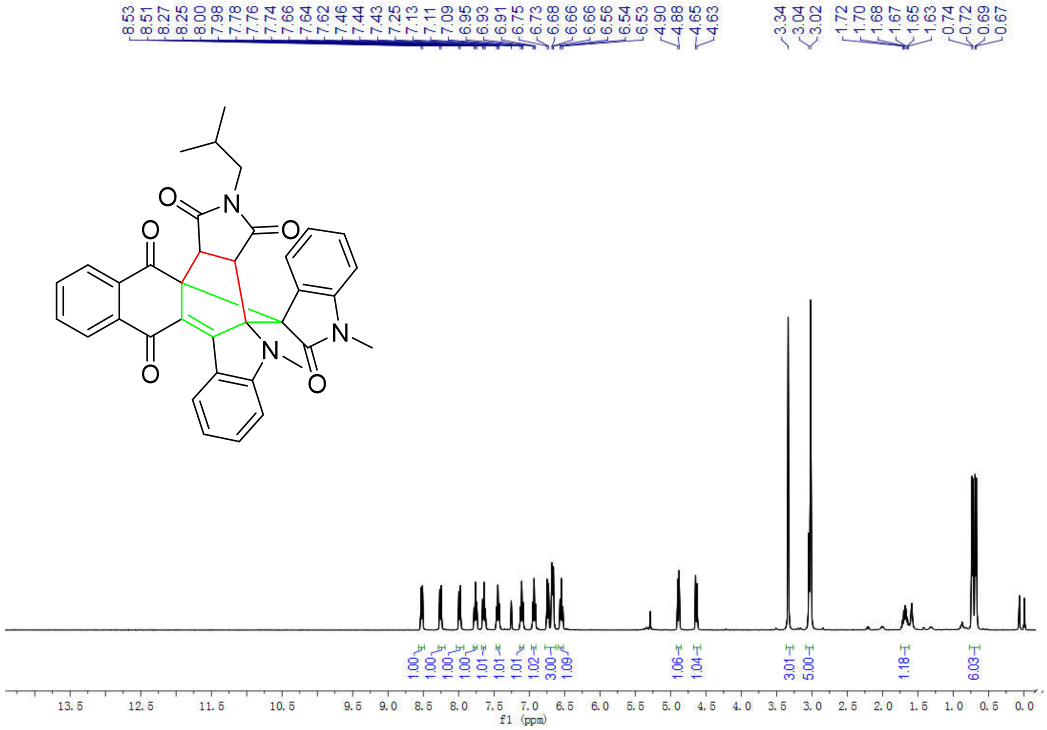

Figure S20. **^{13}C NMR (101 MHz, CDCl$_3$) spectrum of 3ae**

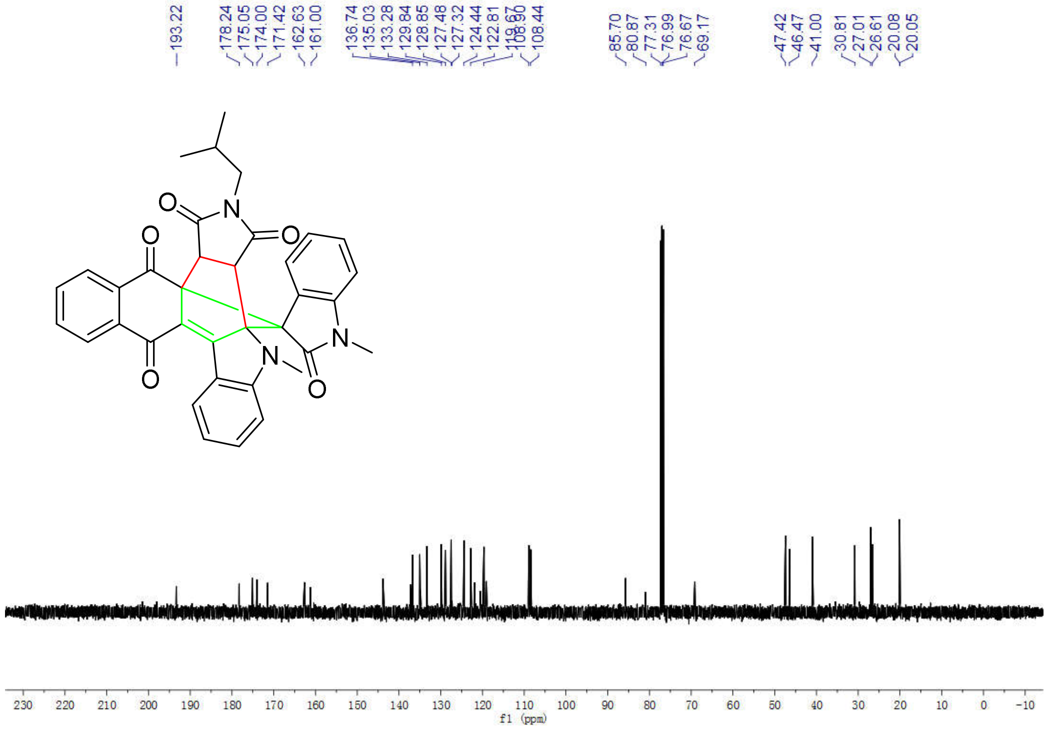

Figure S21. **^{1}H NMR (400 MHz, CDCl$_3$) spectrum of 3af**

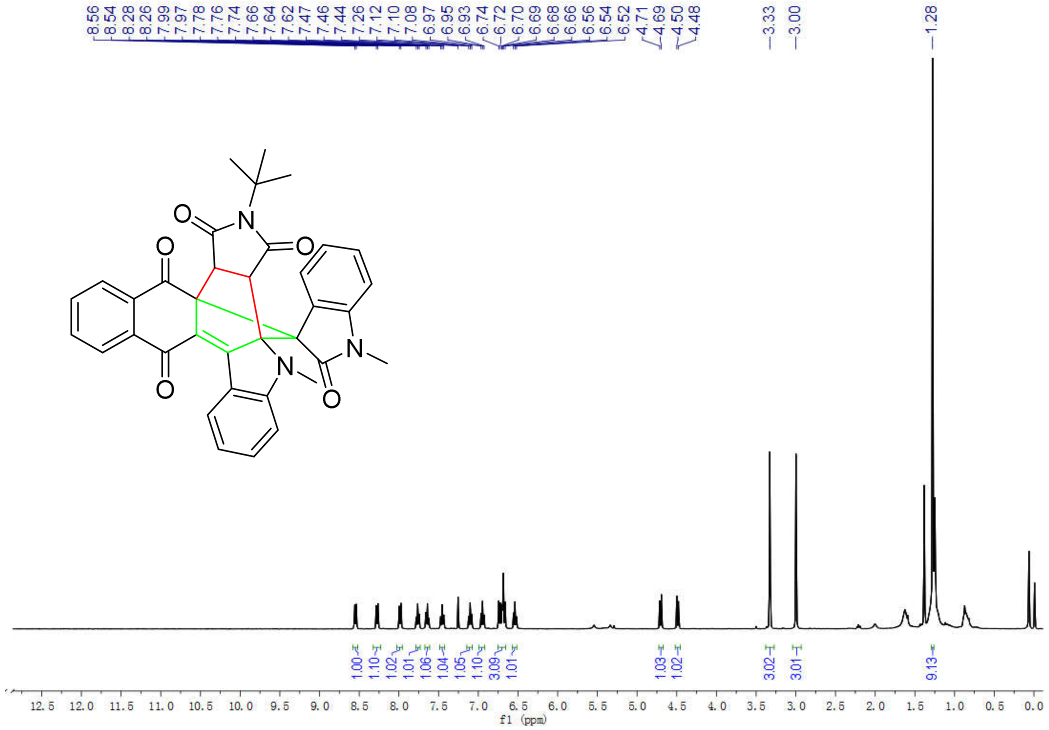

Figure S22. ^{13}C NMR (101 MHz, $CDCl_3$) spectrum of 3af

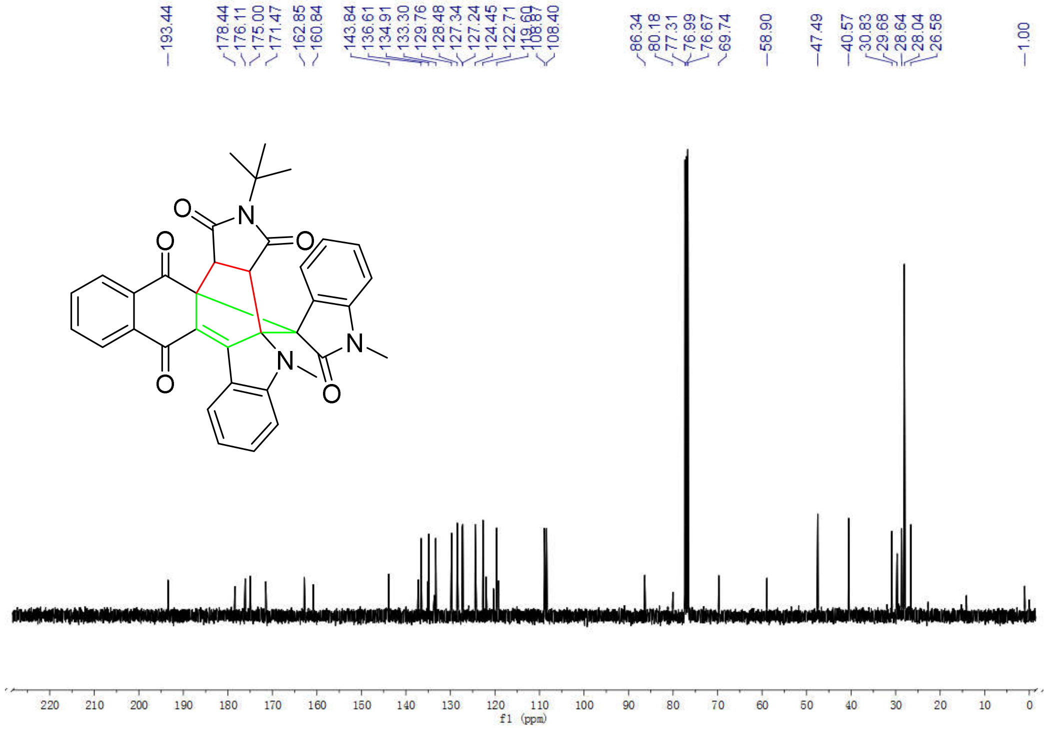

Figure S23. ^{1}H NMR (400 MHz, $CDCl_3$) spectrum of 3ag

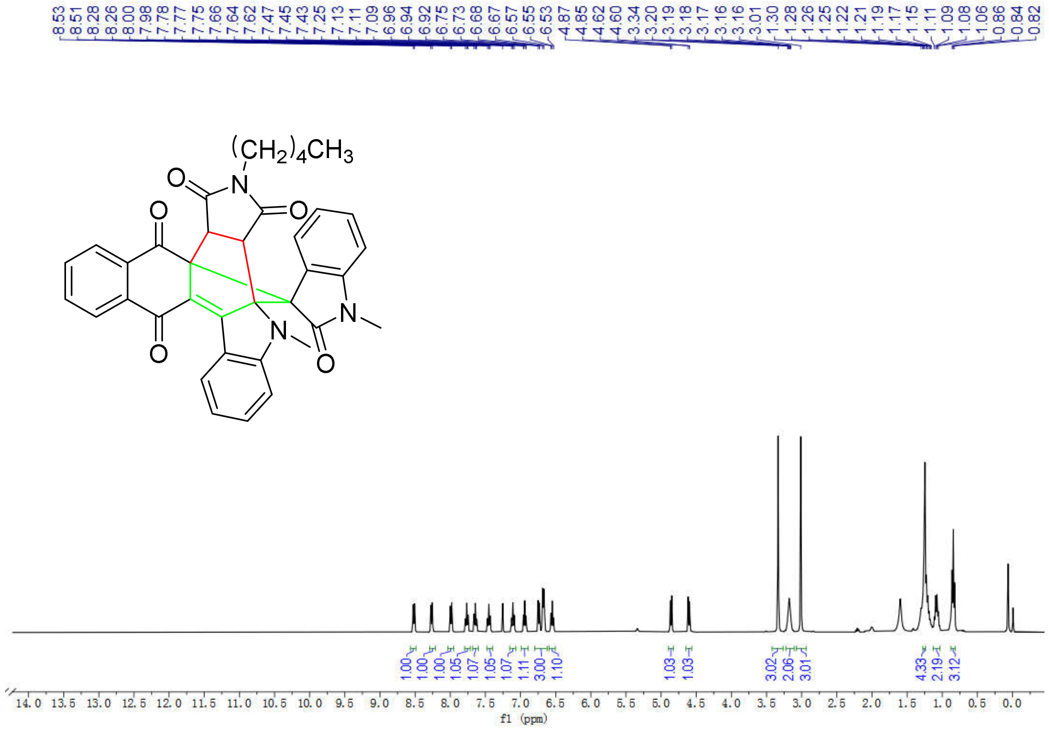

Figure S24. ^{13}**C NMR (101 MHz, CDCl**$_3$**) spectrum of 3ag**

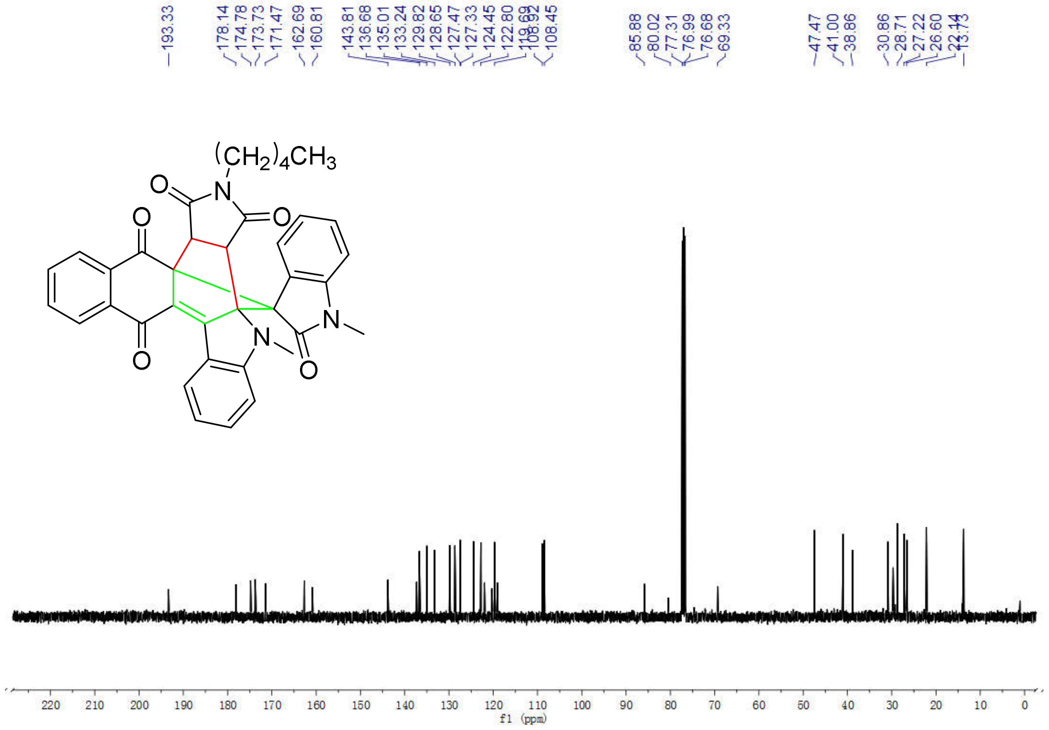

Figure S25. ^{1}**H NMR (400 MHz, CDCl**$_3$**) spectrum of 3ah**

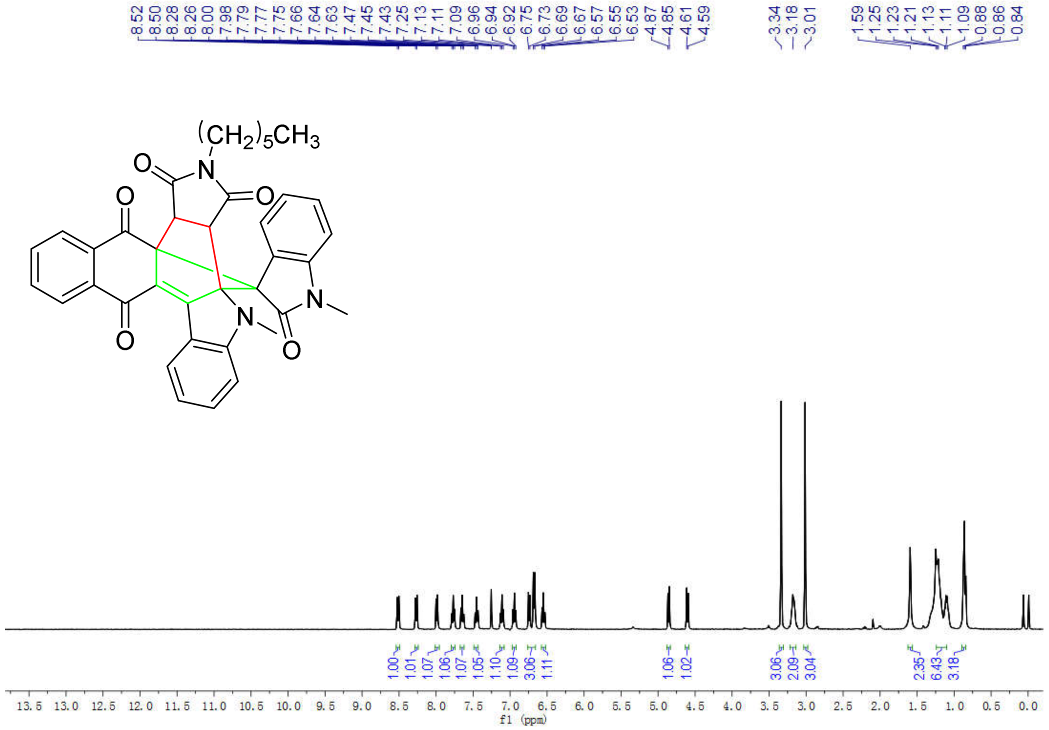

Figure S26. ^{13}C NMR (101 MHz, $CDCl_3$) spectrum of 3ah

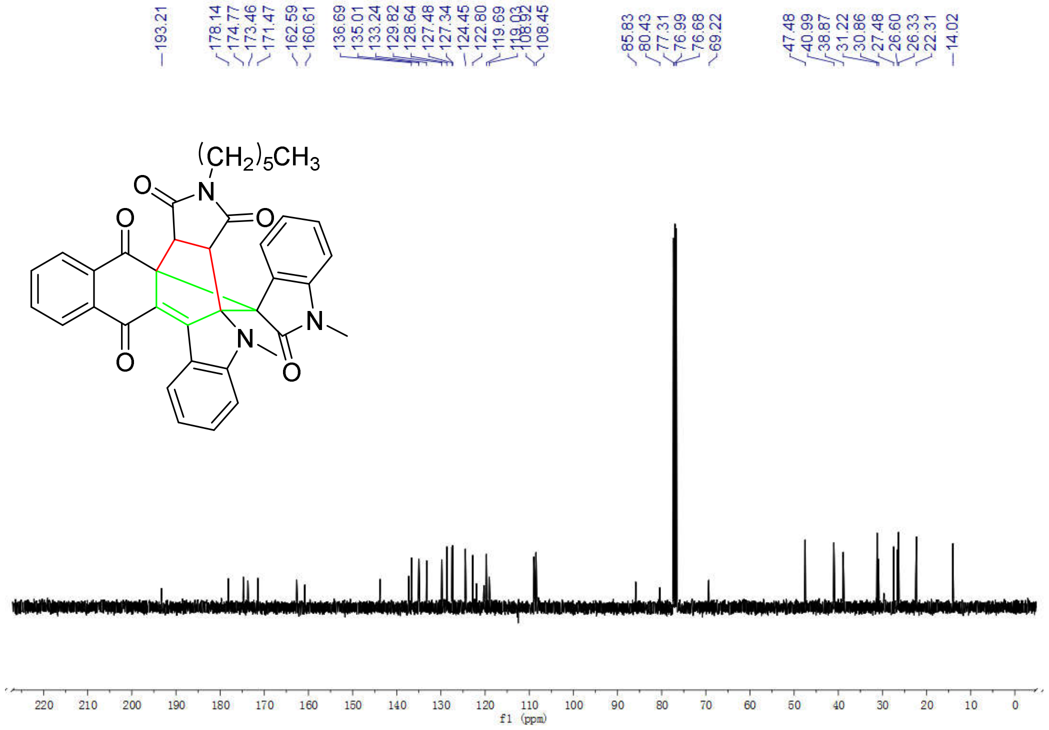

Figure S27. ^{1}H NMR (400 MHz, $CDCl_3$) spectrum of 3ai

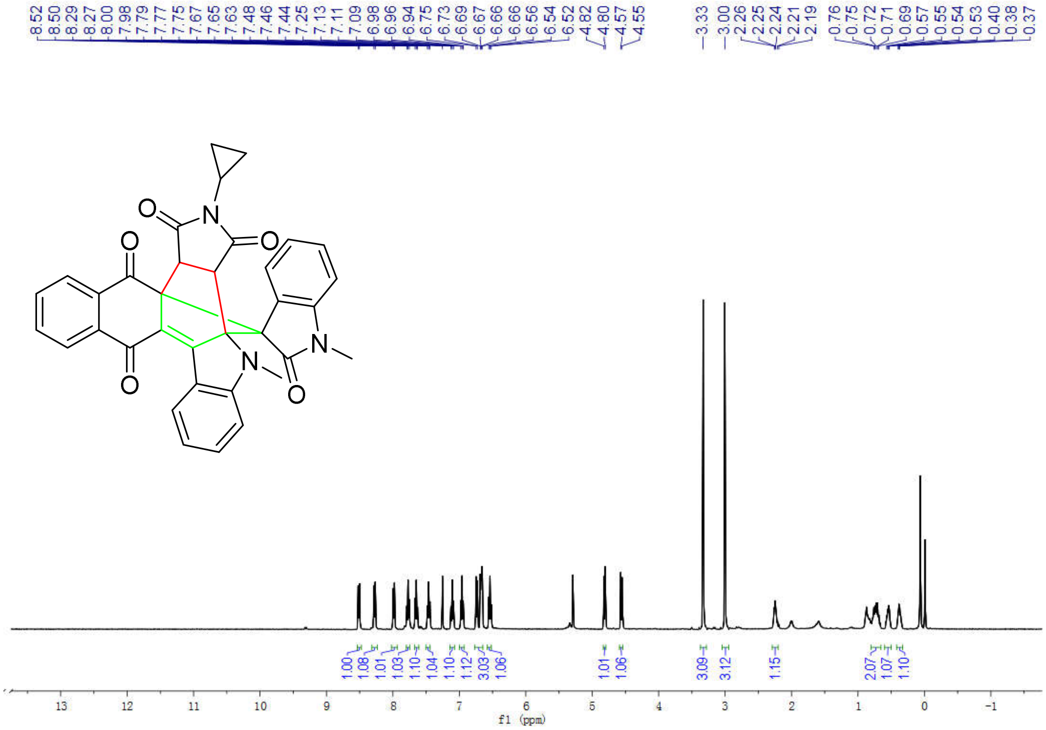

Figure S28. ^{13}**C NMR (101 MHz, CDCl$_3$) spectrum of 3ai**

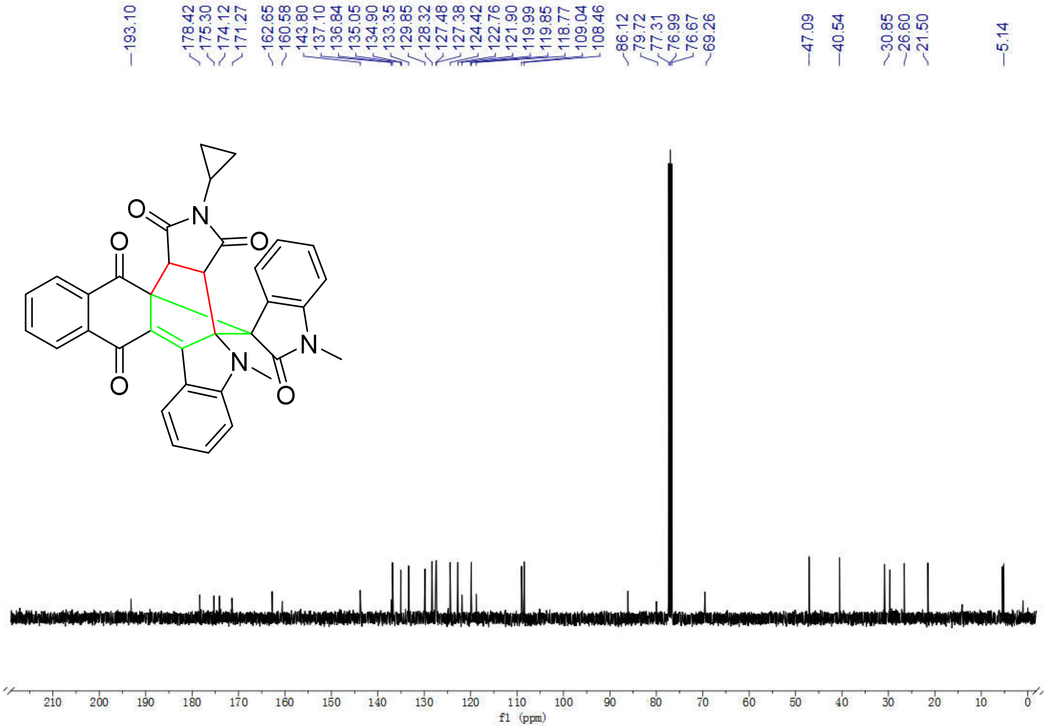

Figure S29. ^{1}**H NMR (400 MHz, CDCl$_3$) spectrum of 3aj**

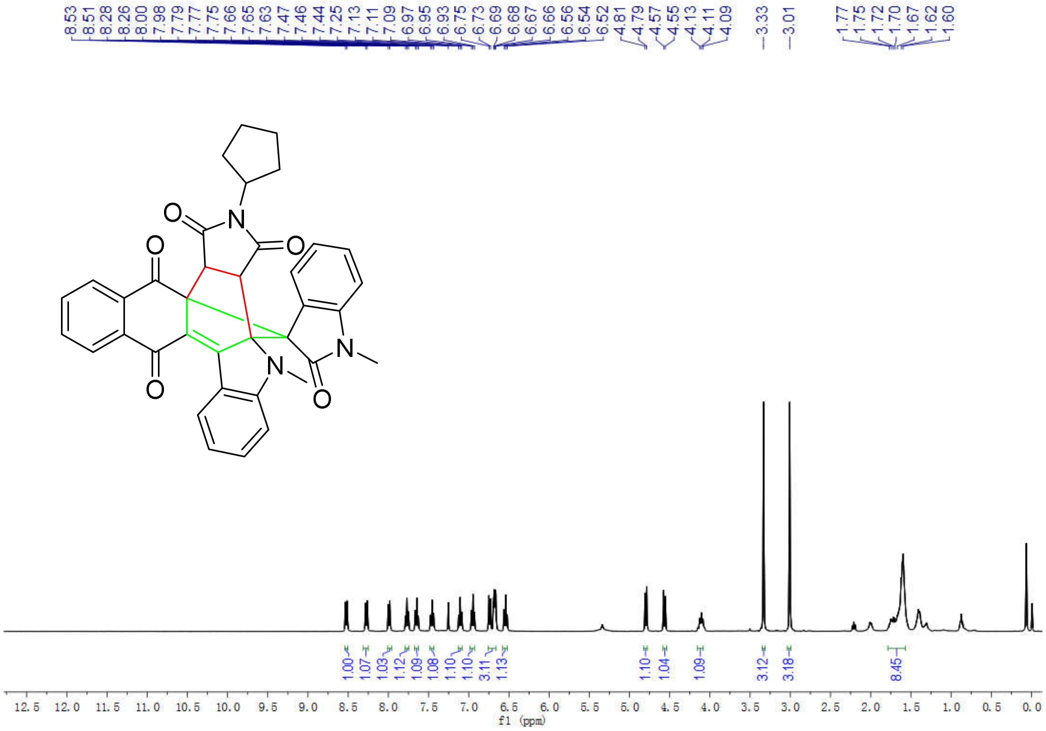

Figure S30. ^{13}C NMR (101 MHz, CDCl$_3$) spectrum of 3aj

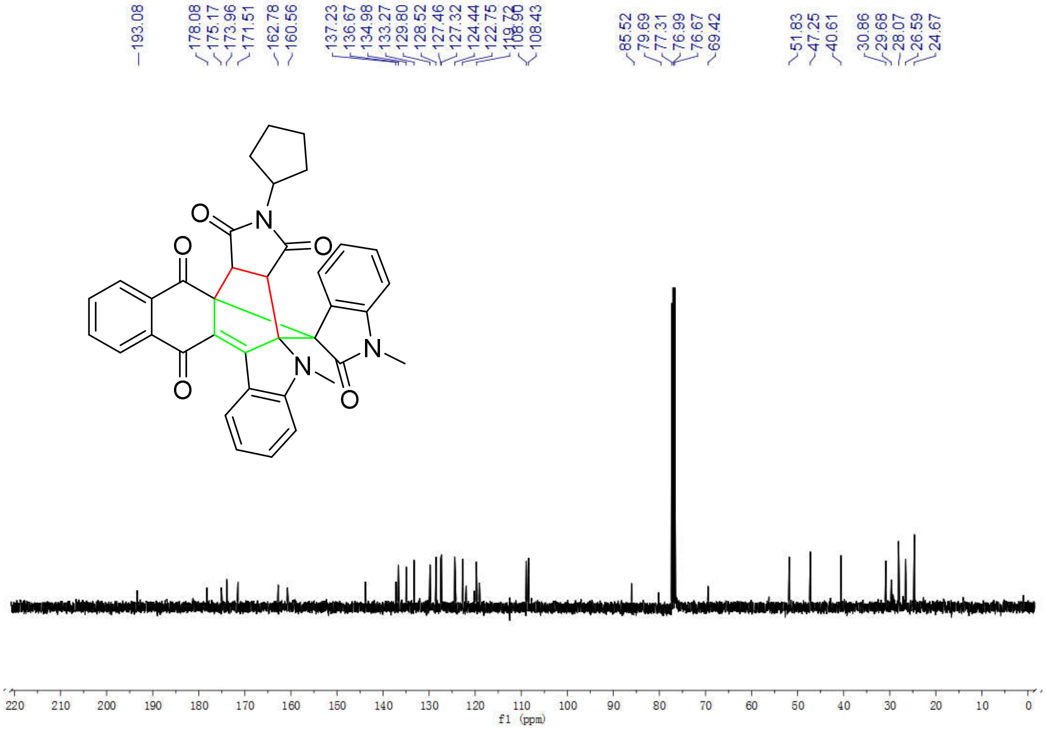

Figure S31. ^{1}H NMR (400 MHz, CDCl$_3$) spectrum of 3ak

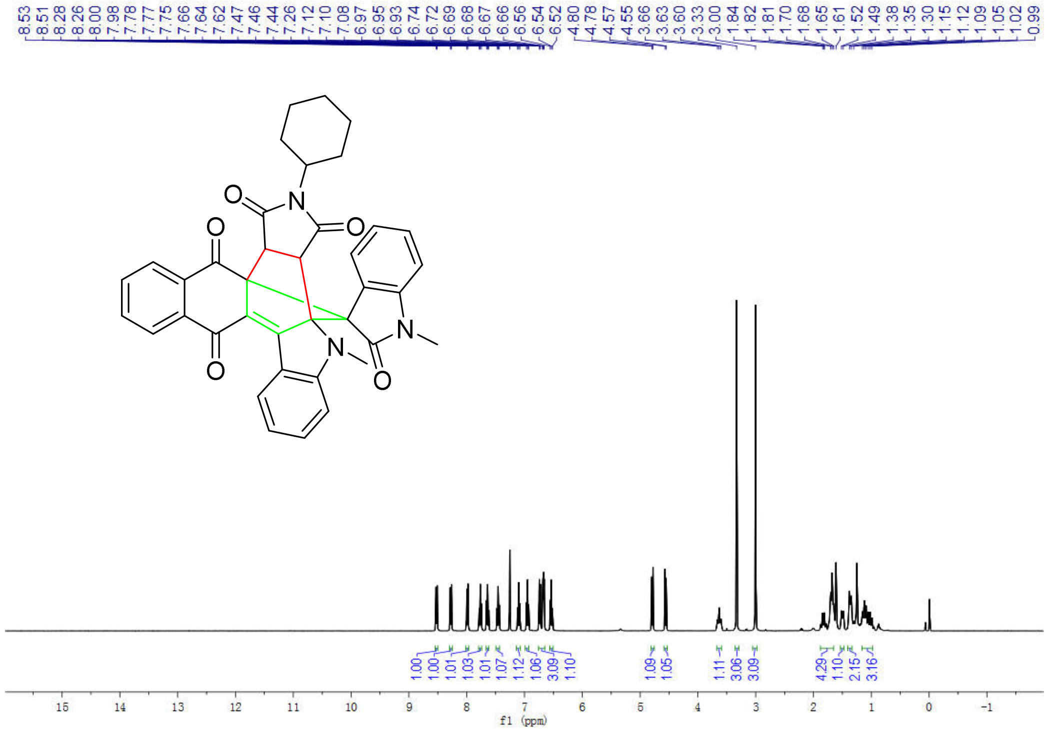

Figure S32. ^{13}C NMR (101 MHz, CDCl3) spectrum of 3ak

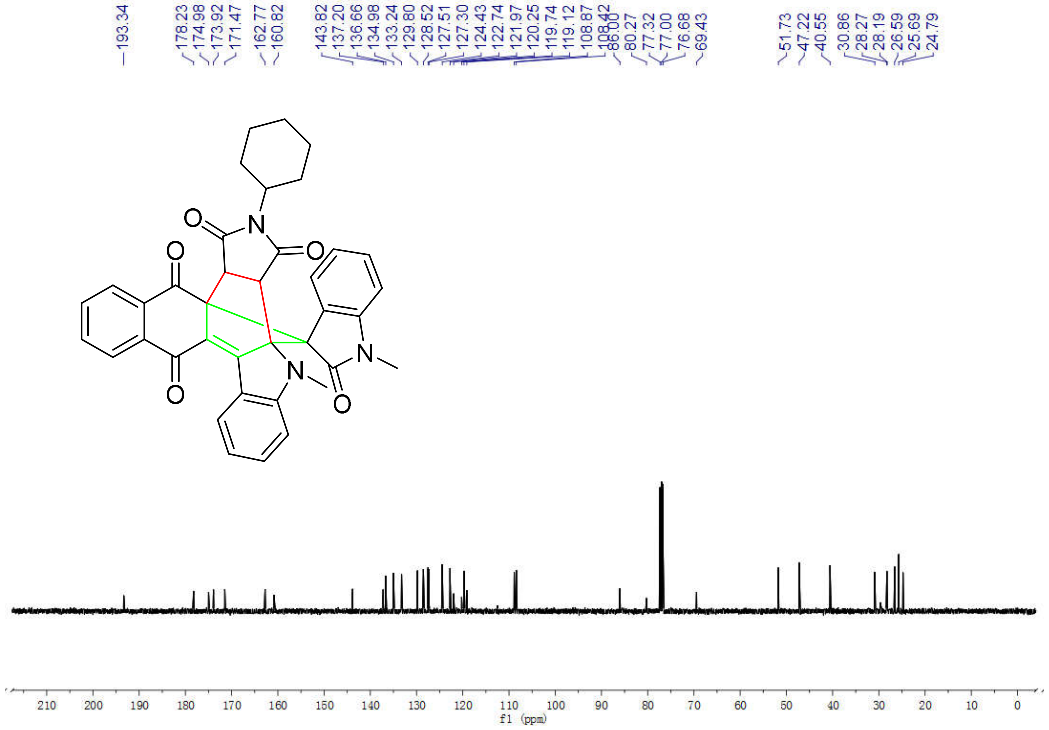

Figure S33. ^{1}H NMR (400 MHz, CDCl3) spectrum of 3al

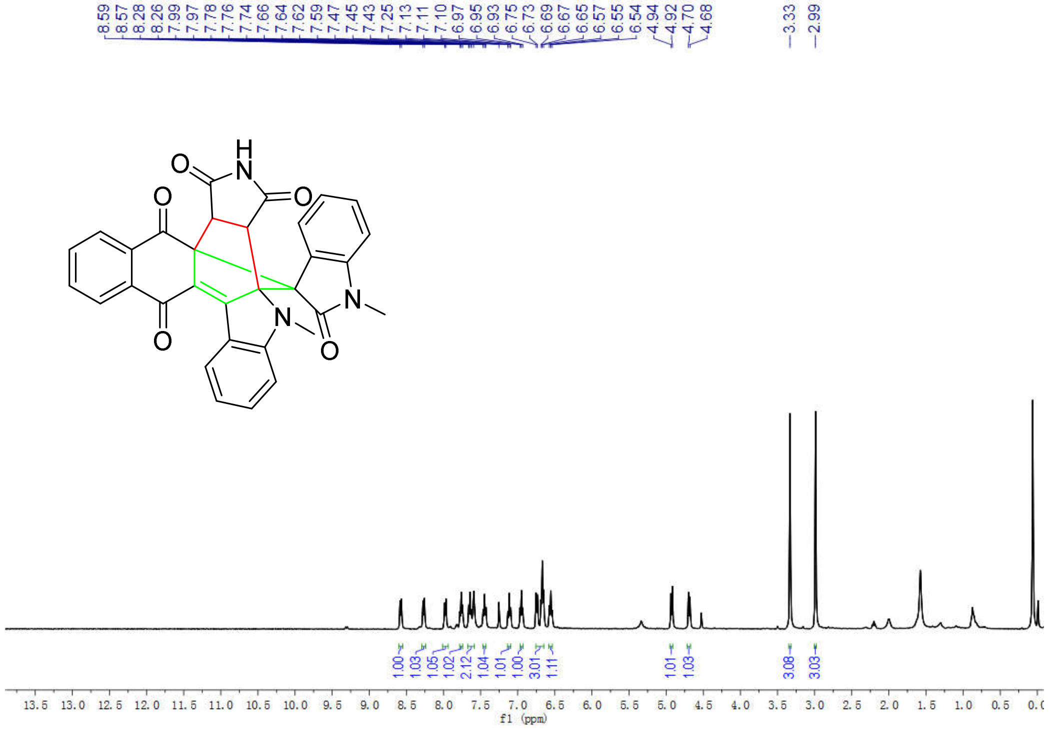

Figure S34. ^{13}C NMR (101 MHz, $CDCl_3$) spectrum of 3al

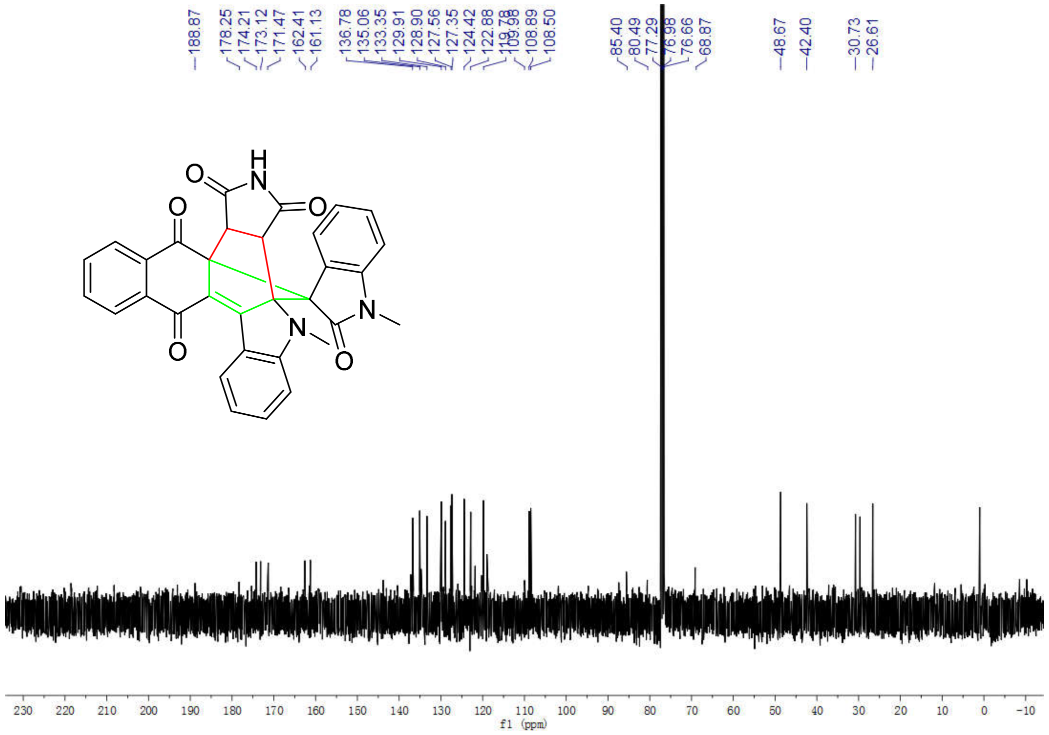

Figure S35. ^{1}H NMR (400 MHz, $CDCl_3$) spectrum of 3am

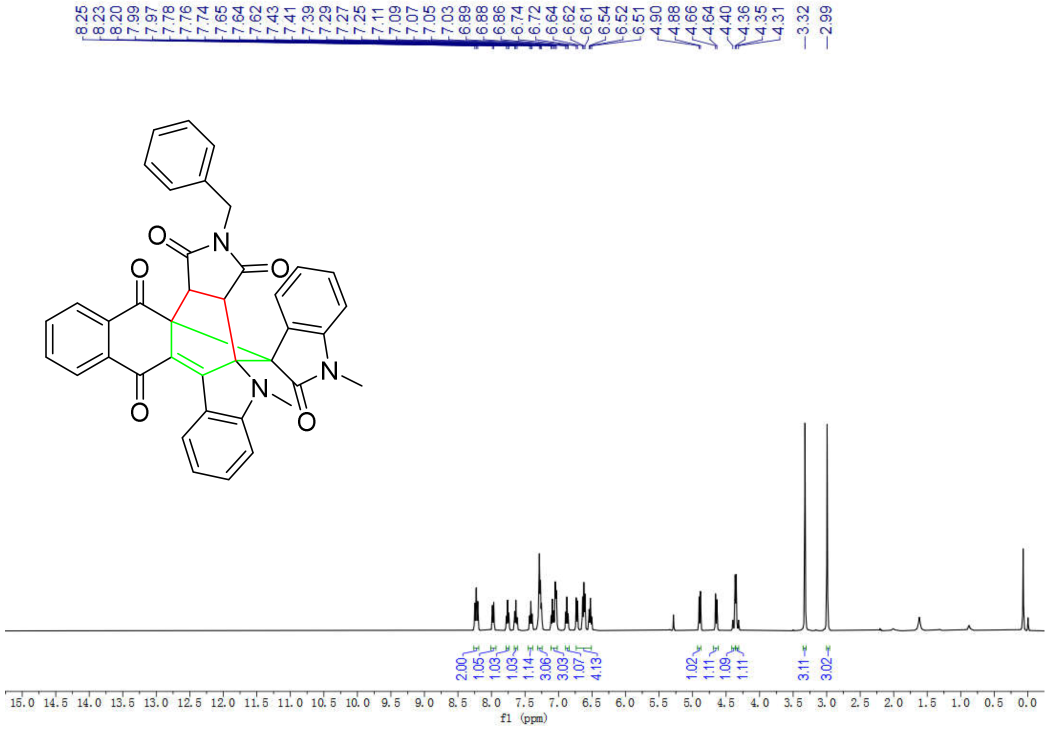

Figure S36. ^{13}C NMR (101 MHz, CDCl$_3$) spectrum of 3am

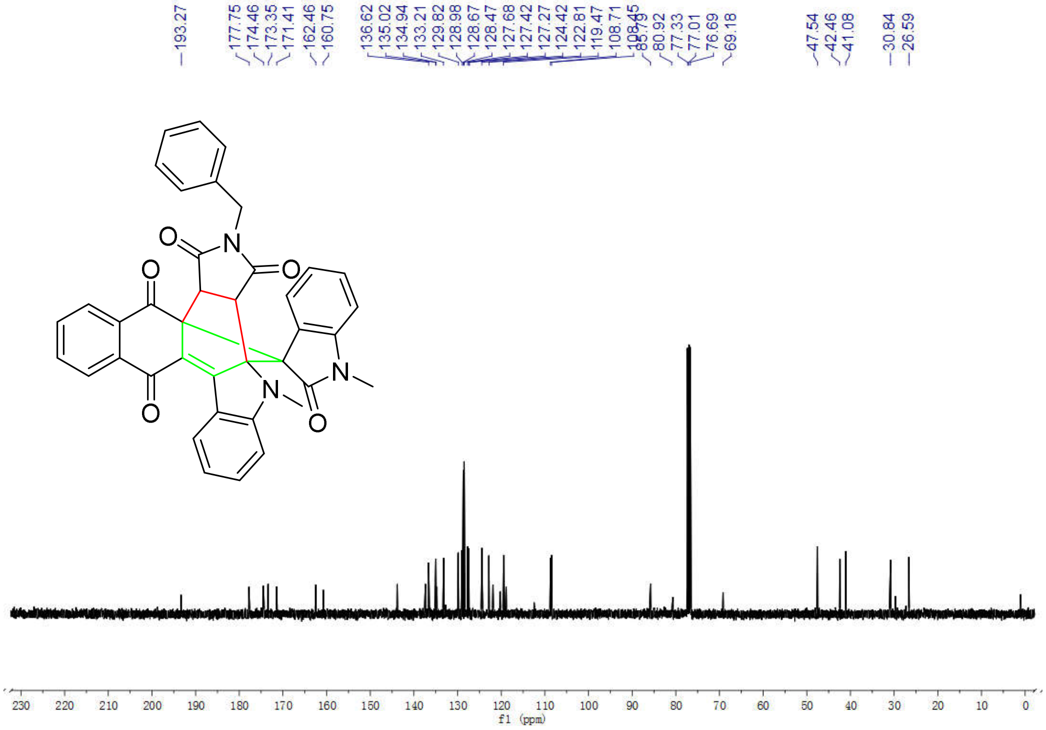

Figure S37. ^{1}H NMR (400 MHz, CDCl$_3$) spectrum of 3an

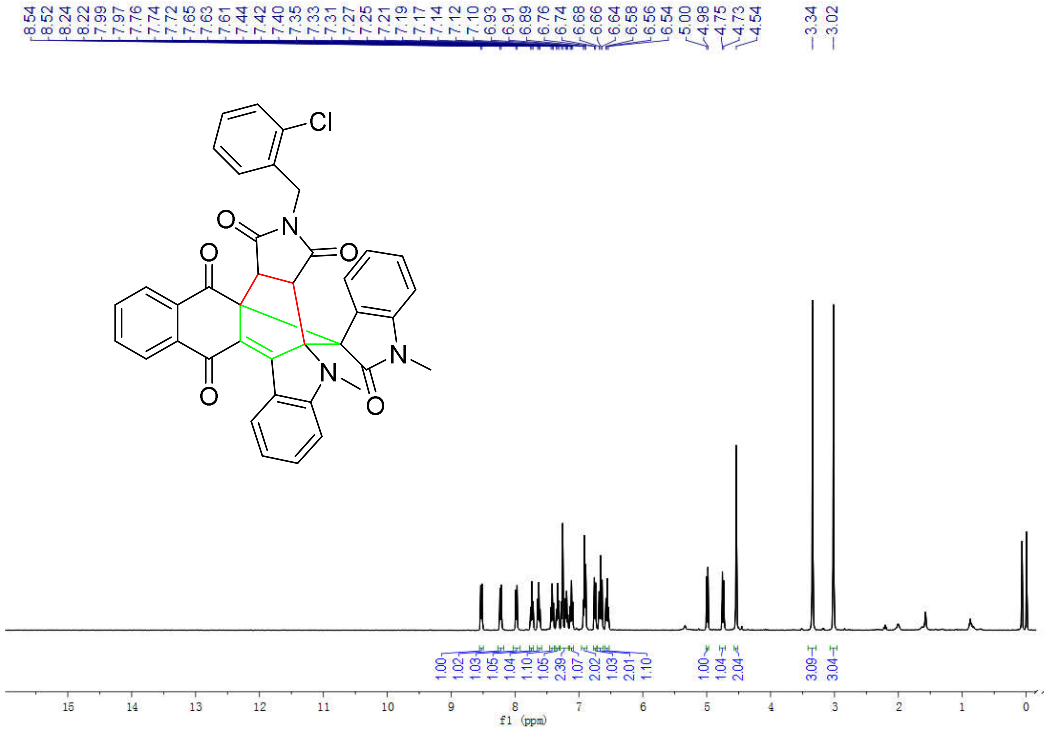

Figure S38. ^{13}C NMR (101 MHz, CDCl$_3$) spectrum of 3an

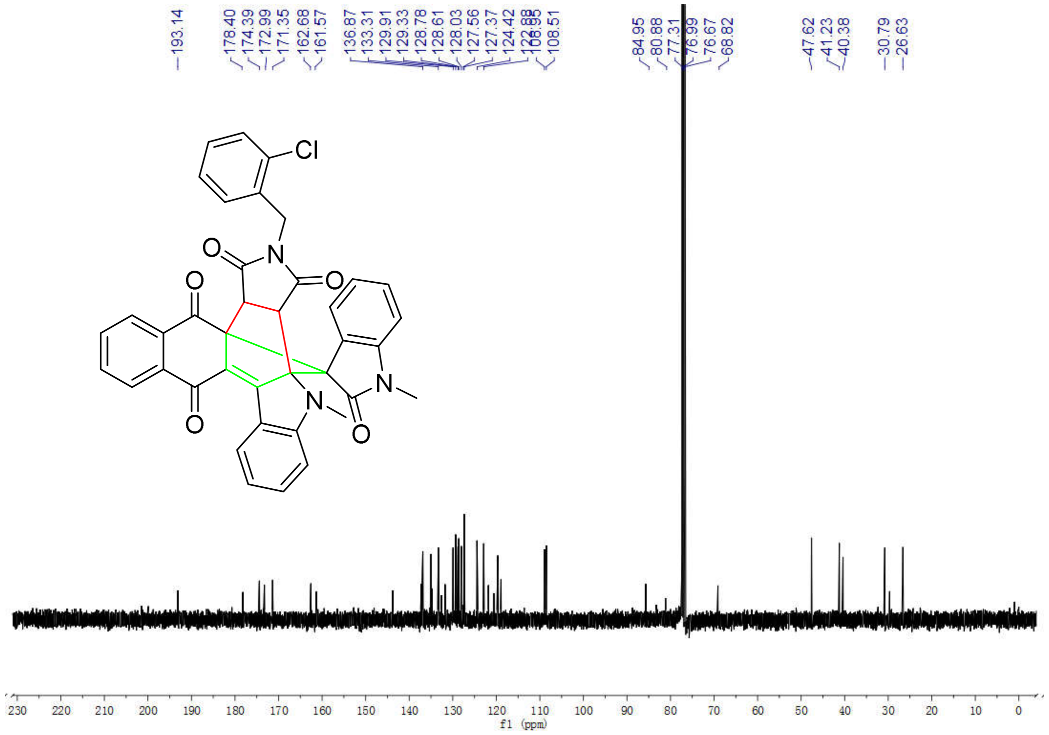

Figure S39. ^{1}H NMR (400 MHz, CDCl$_3$) spectrum of 3ao

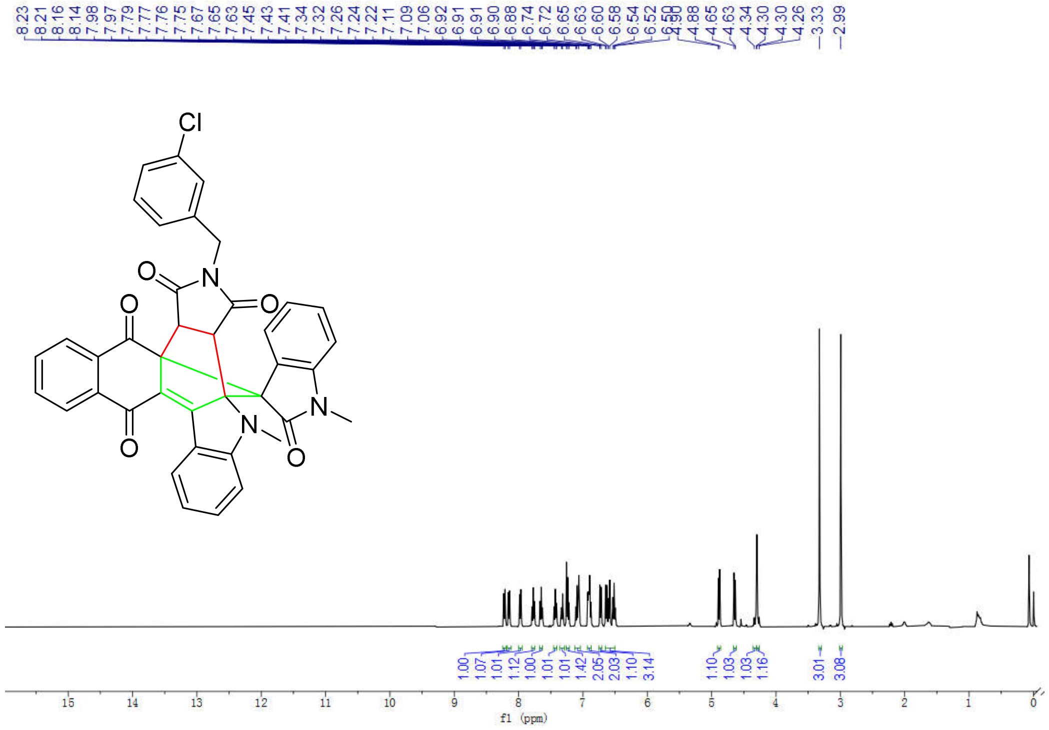

Figure S40. ^{13}C NMR (101 MHz, $CDCl_3$) spectrum of 3ao

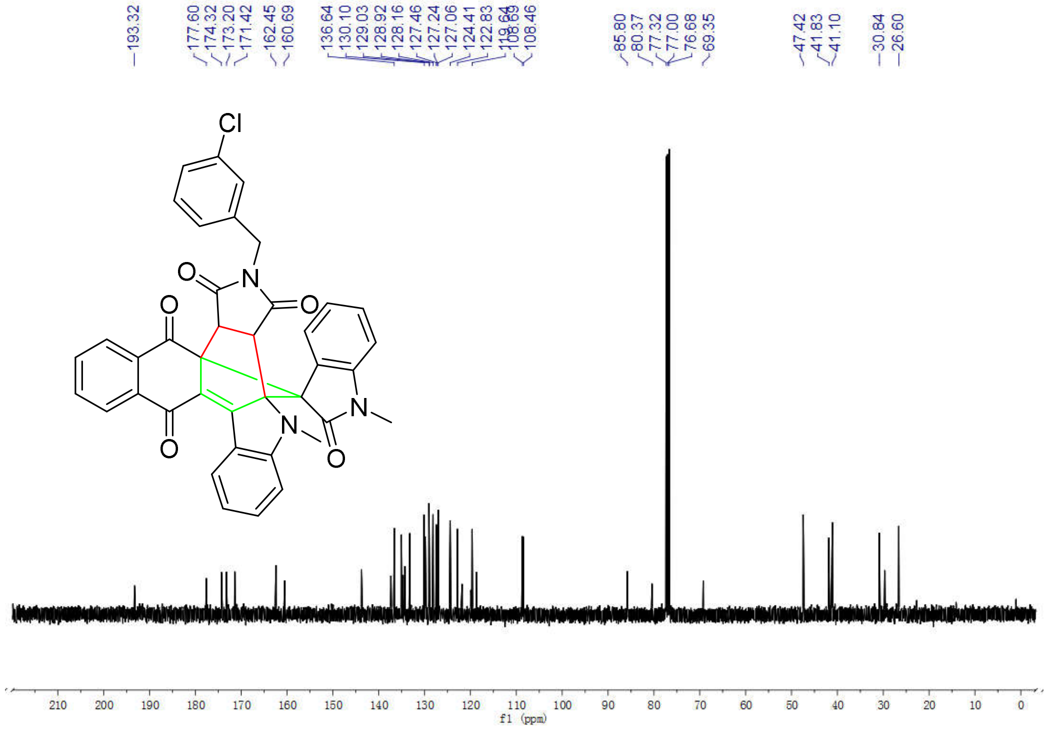

Figure S41. ^{1}H NMR (400 MHz, $CDCl_3$) spectrum of 3ap

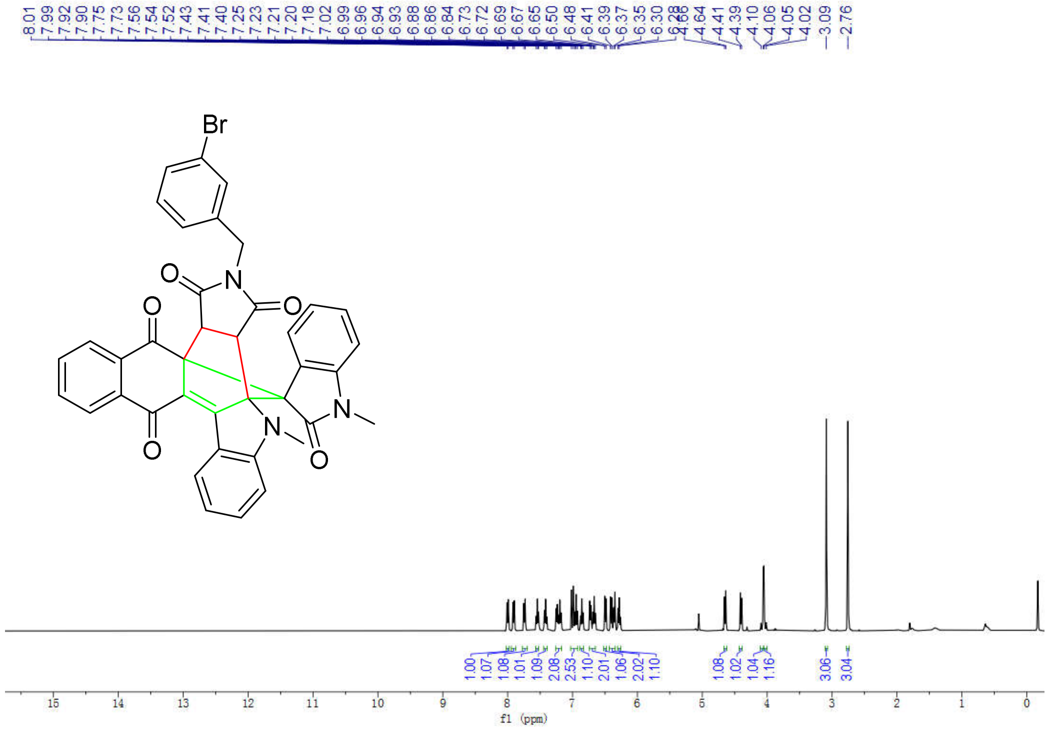

Figure S42. ^{13}C NMR (101 MHz, CDCl$_3$) spectrum of 3ap

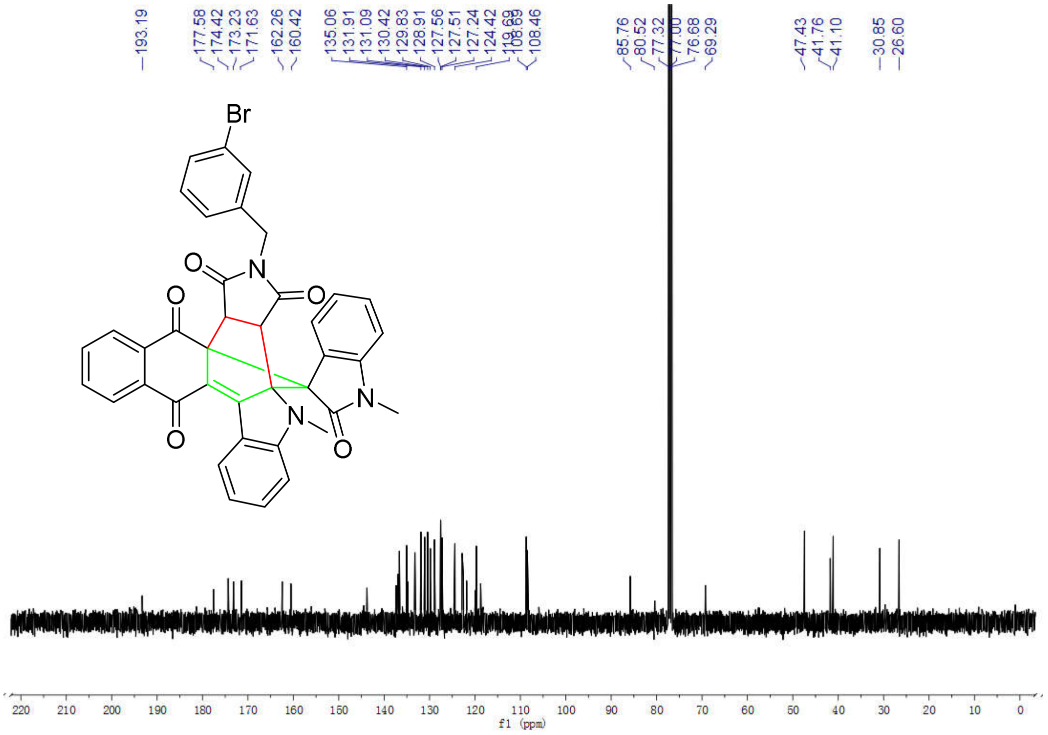

Figure S43. ^{1}H NMR (400 MHz, CDCl$_3$) spectrum of 3aq

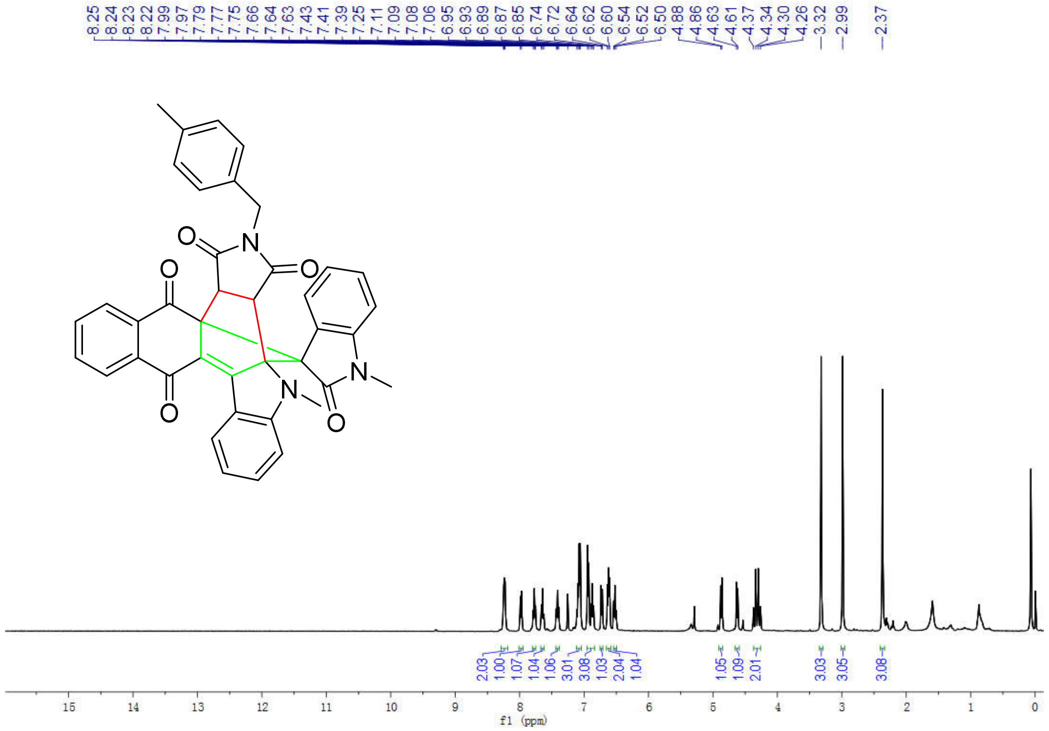

Figure S44. ^{13}**C NMR (101 MHz,** $CDCl_3$**) spectrum of 3aq**

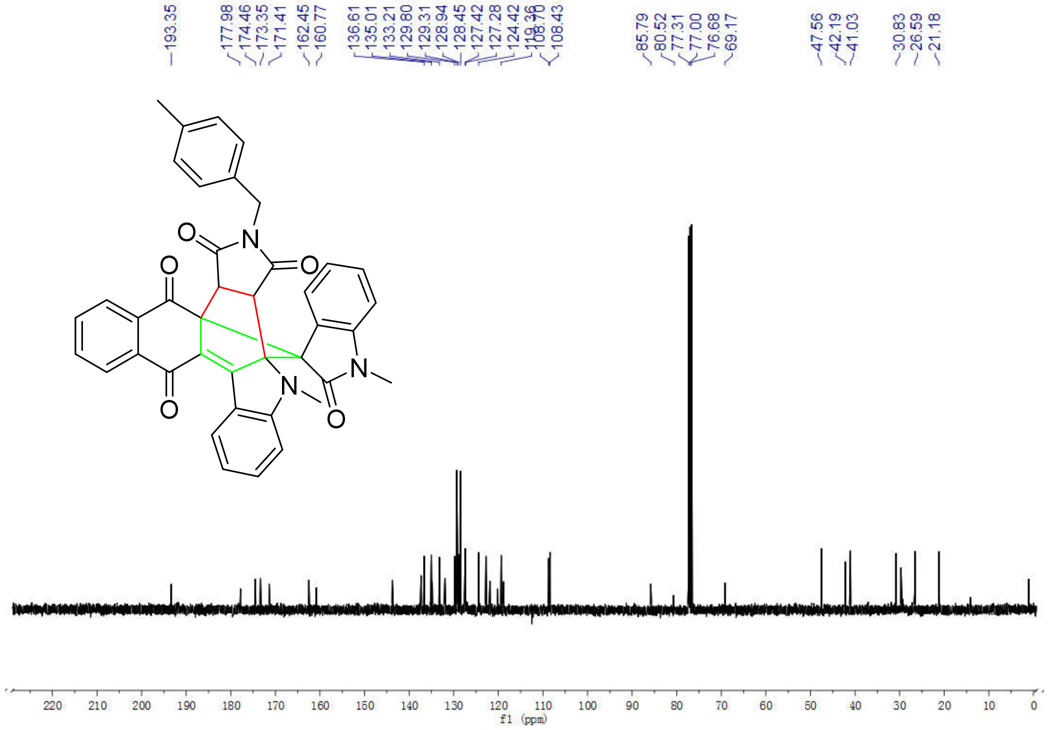

Figure S45. ^{1}**H NMR (400 MHz,** $CDCl_3$**) spectrum of 3ar**

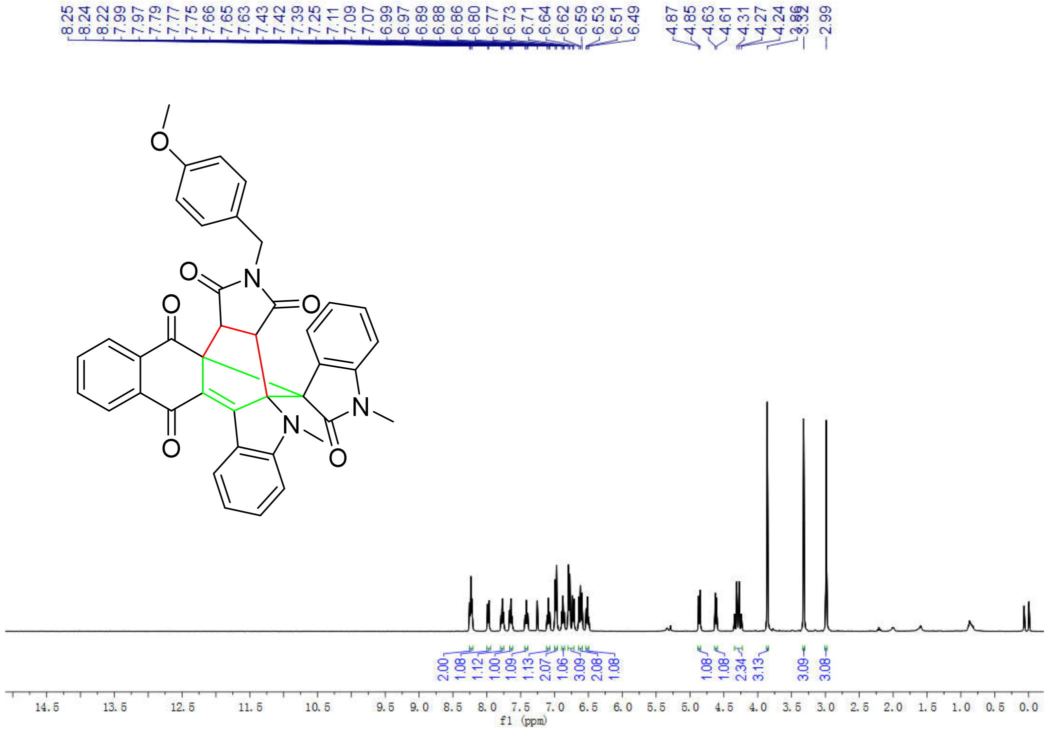

Figure S46. ^{13}C NMR (101 MHz, CDCl$_3$) spectrum of 3ar

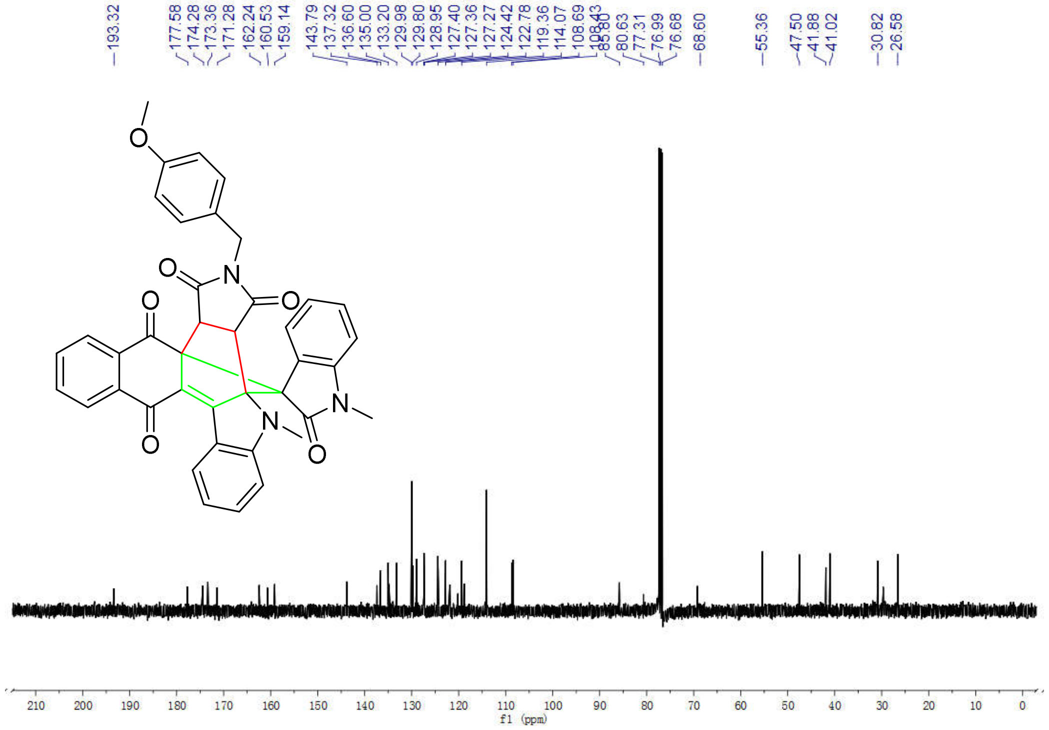

Figure S47. ^{1}H NMR (400 MHz, CDCl$_3$) spectrum of 3as

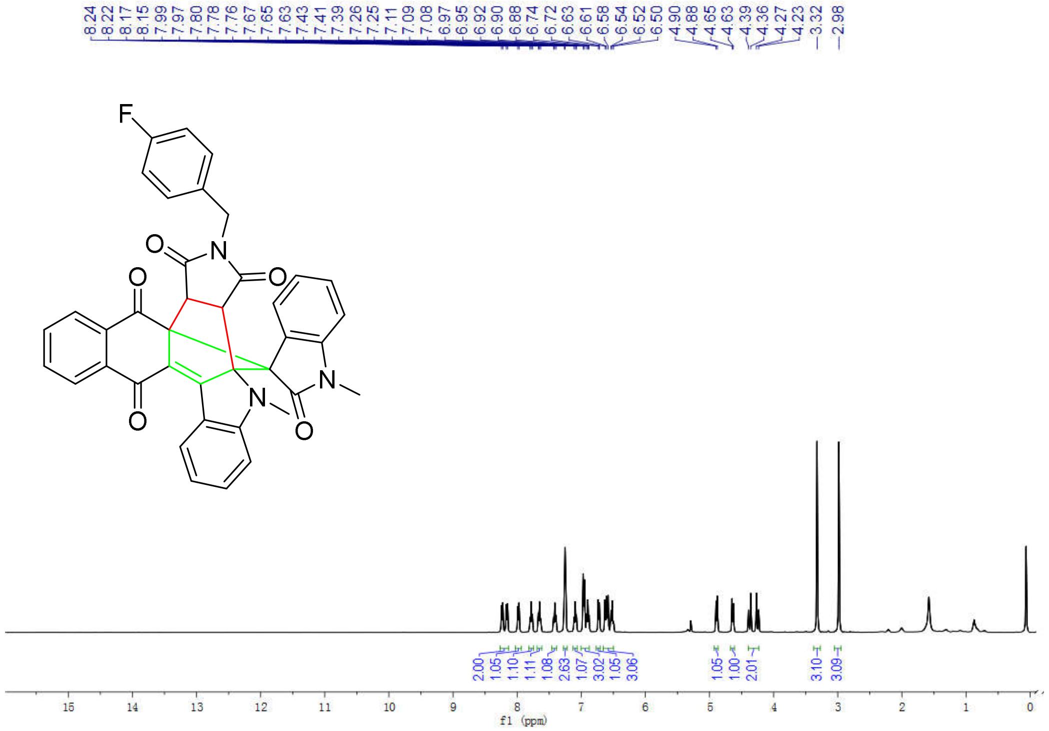

Figure S48. ^{13}C NMR (101 MHz, $CDCl_3$) spectrum of 3as

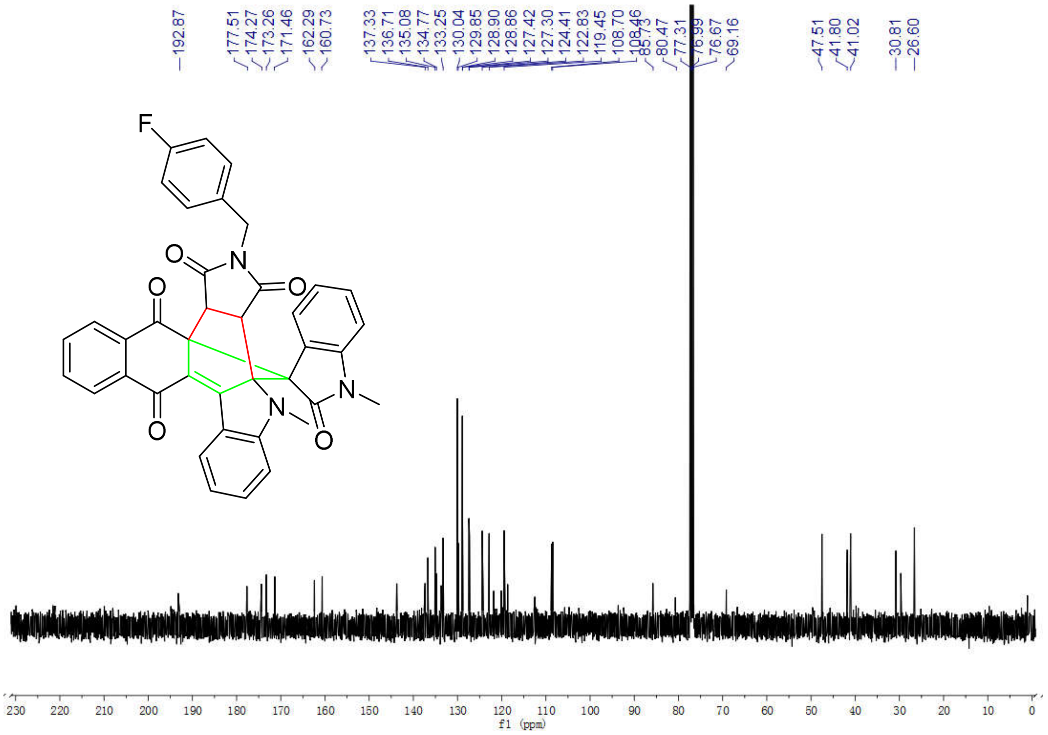

Figure S49. ^{19}F NMR (376 MHz, $CDCl_3$) spectrum of 3as

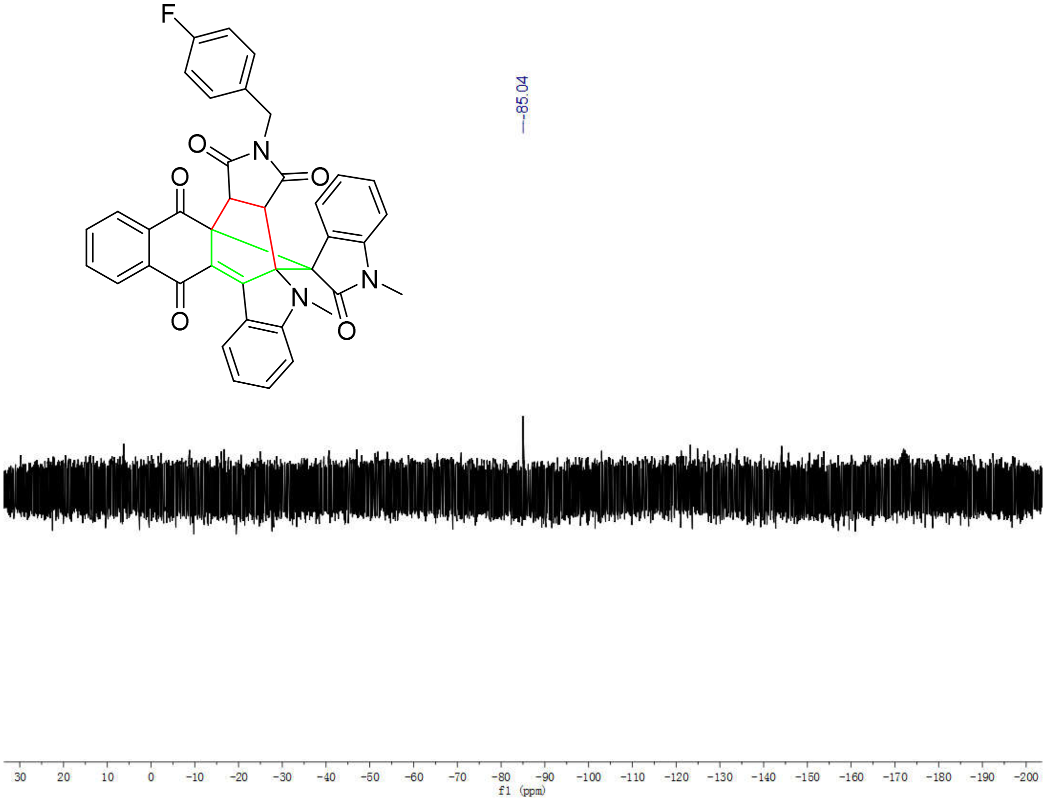

-85.04
30 20 10 0 -10 -20 -30 -40 -50 -60 -70 -80 -90 -100 -110 -120 -130 -140 -150 -160 -170 -180 -190 -200
f1 (ppm)

Figure S50. ^{1}H NMR (400 MHz, CDCl3) spectrum of 3at

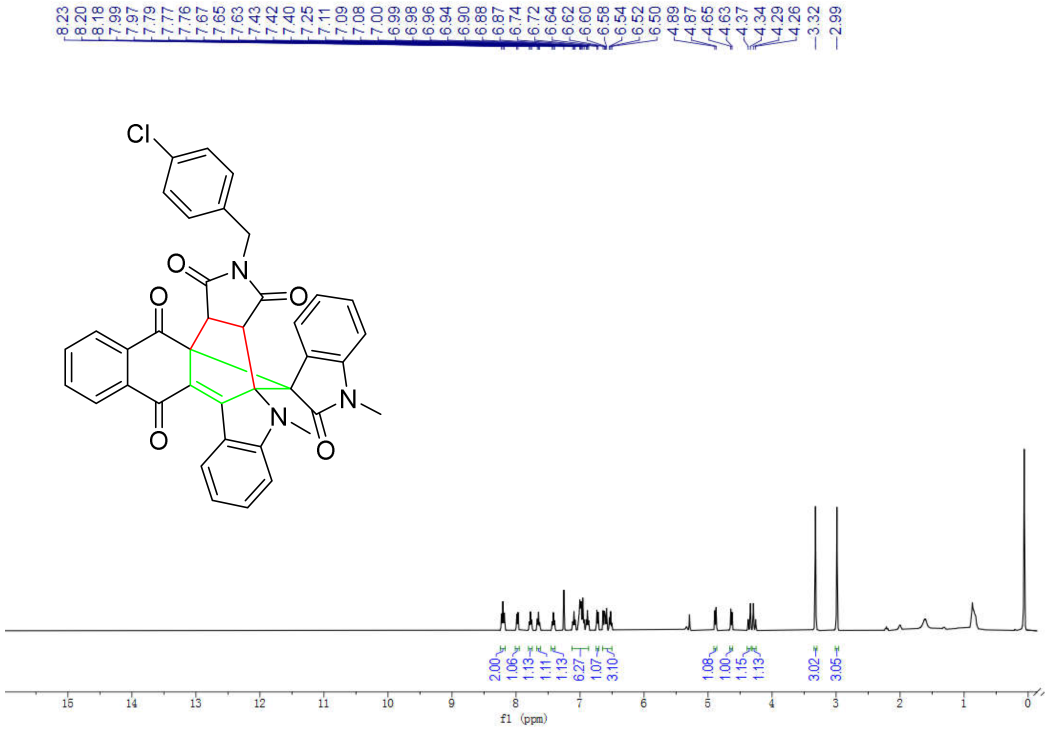

Figure S51. ^{13}C NMR (101 MHz, CDCl3) spectrum of 3at

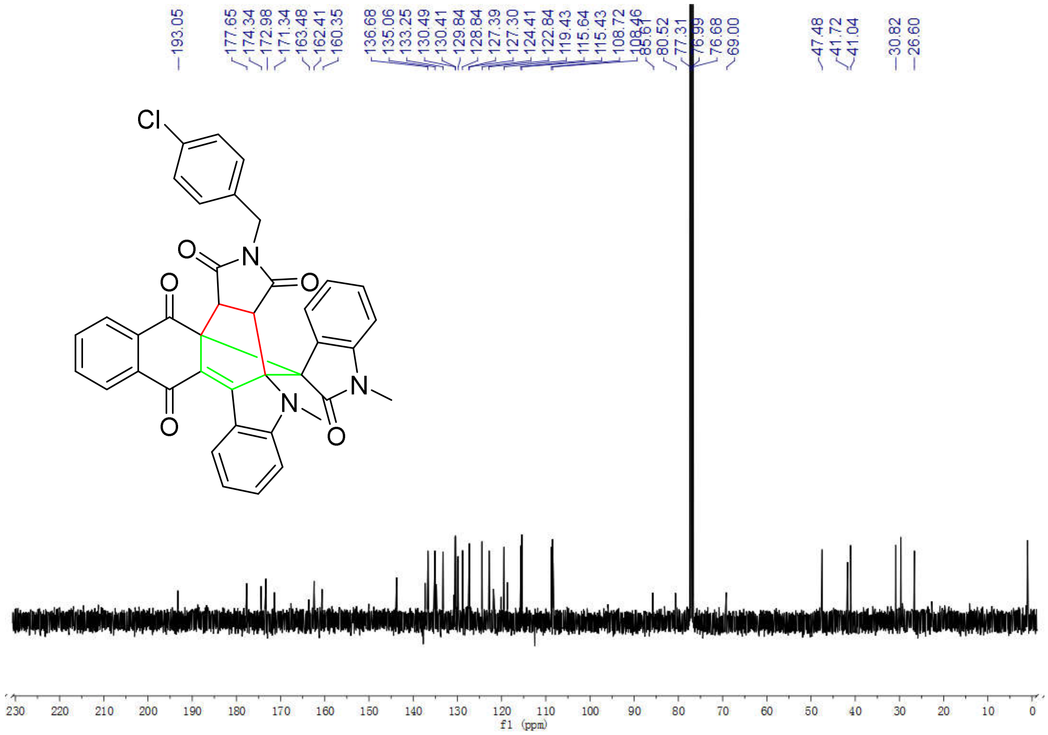

Figure S52. ^{1}H NMR (400 MHz, CDCl3) spectrum of 3au

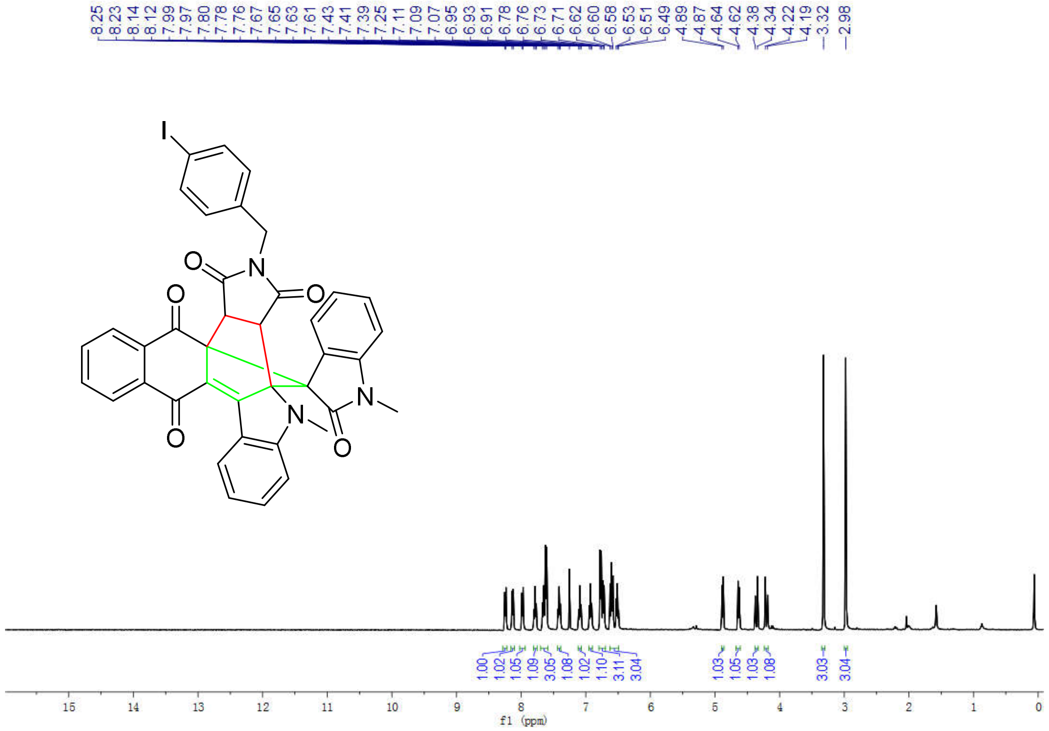

Figure S53. ^{13}C NMR (101 MHz, CDCl3) spectrum of 3au

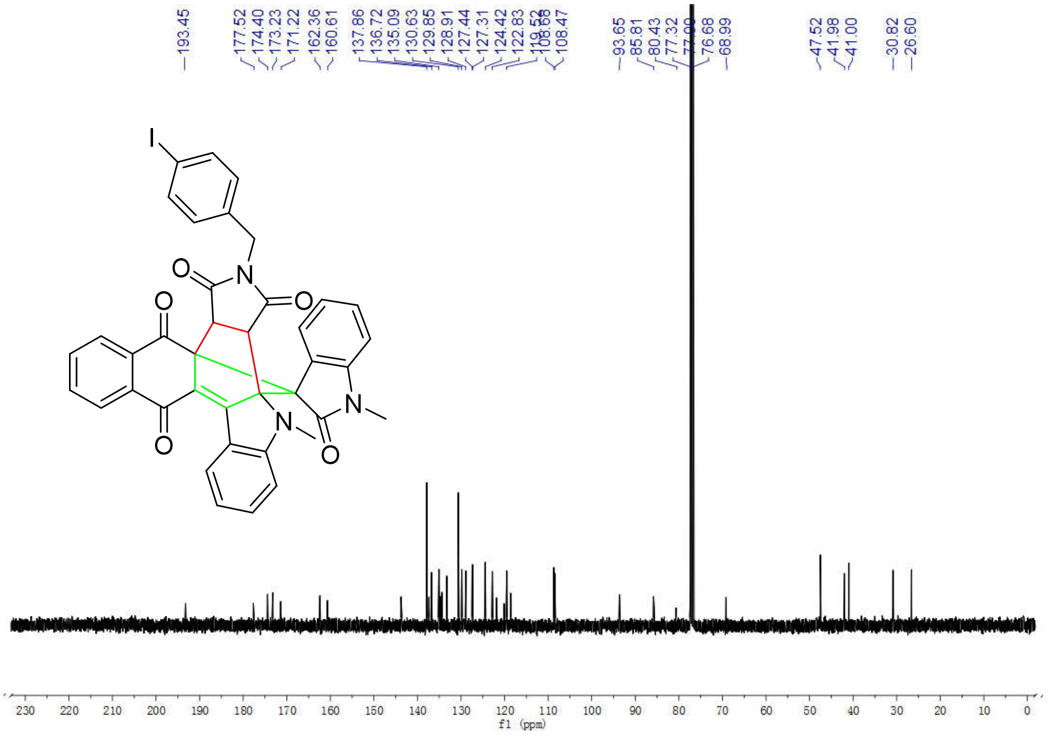

Figure S54. ^{1}H NMR (400 MHz, CDCl3) spectrum of 3av

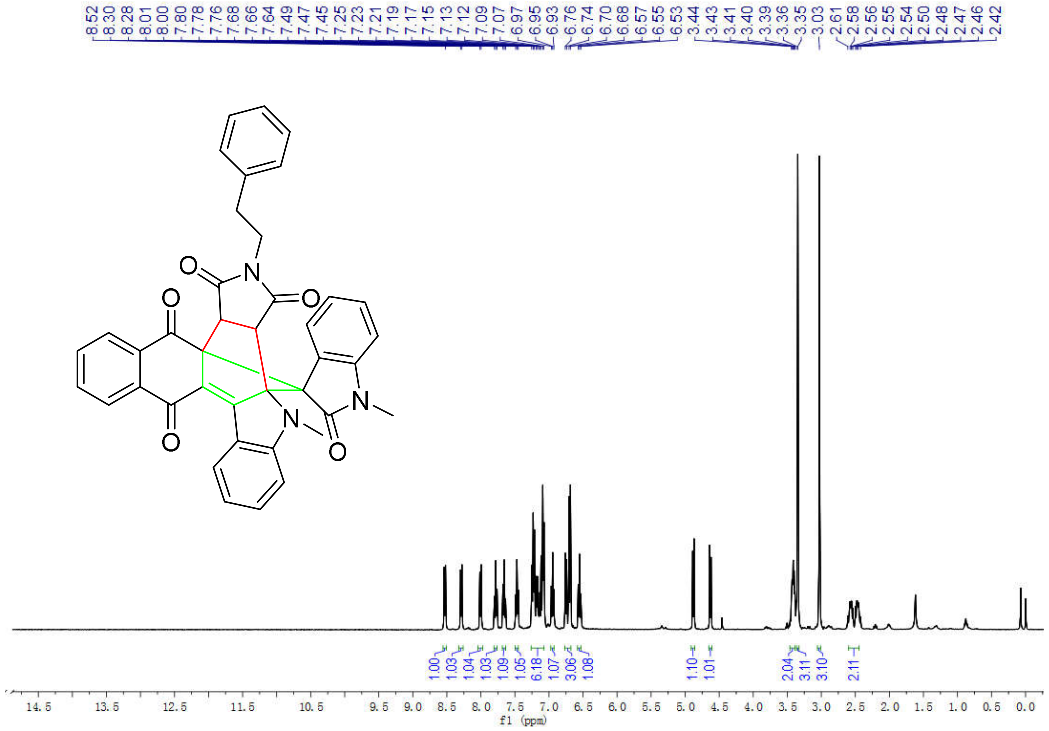

Figure S55. ^{13}**C NMR (101 MHz, CDCl**$_3$**) spectrum of 3av**

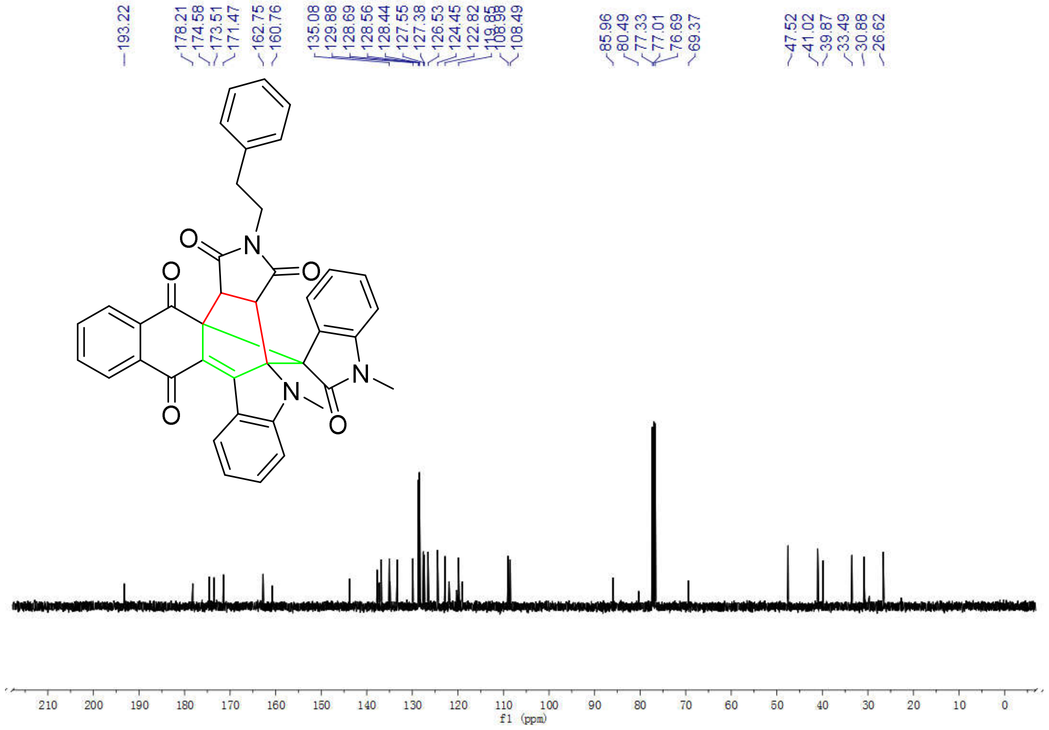

Figure S56. ^{1}**H NMR (400 MHz, CDCl**$_3$**) spectrum of 3aw**

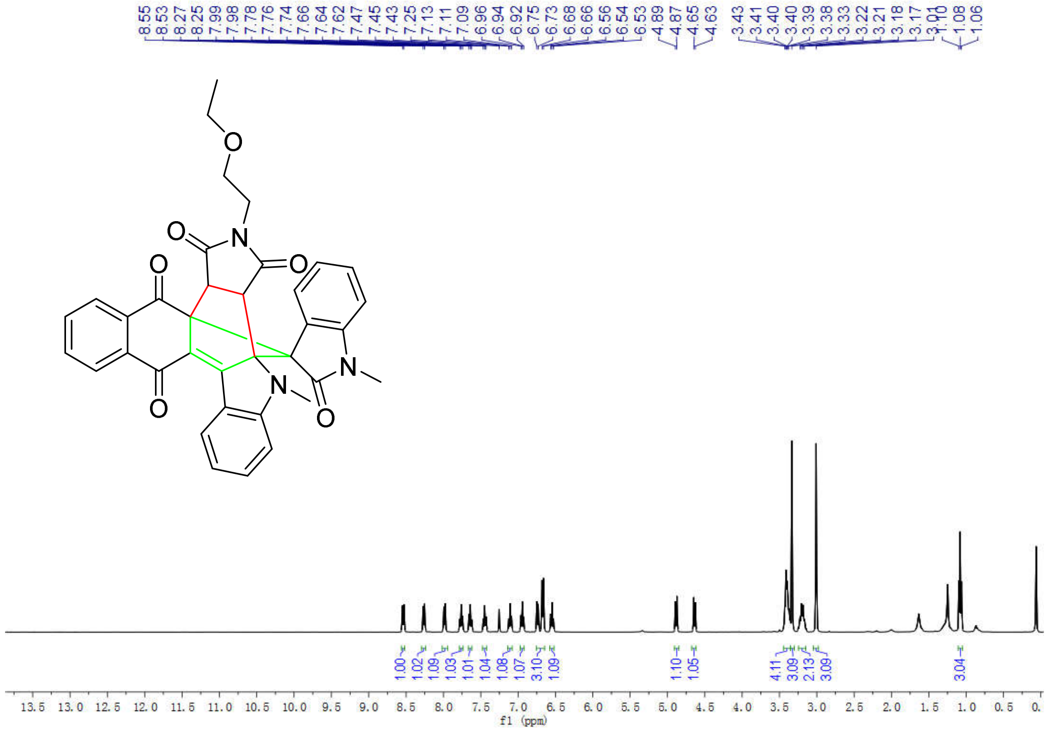

Figure S57. ^{13}C NMR (101 MHz, CDCl$_3$) spectrum of 3aw

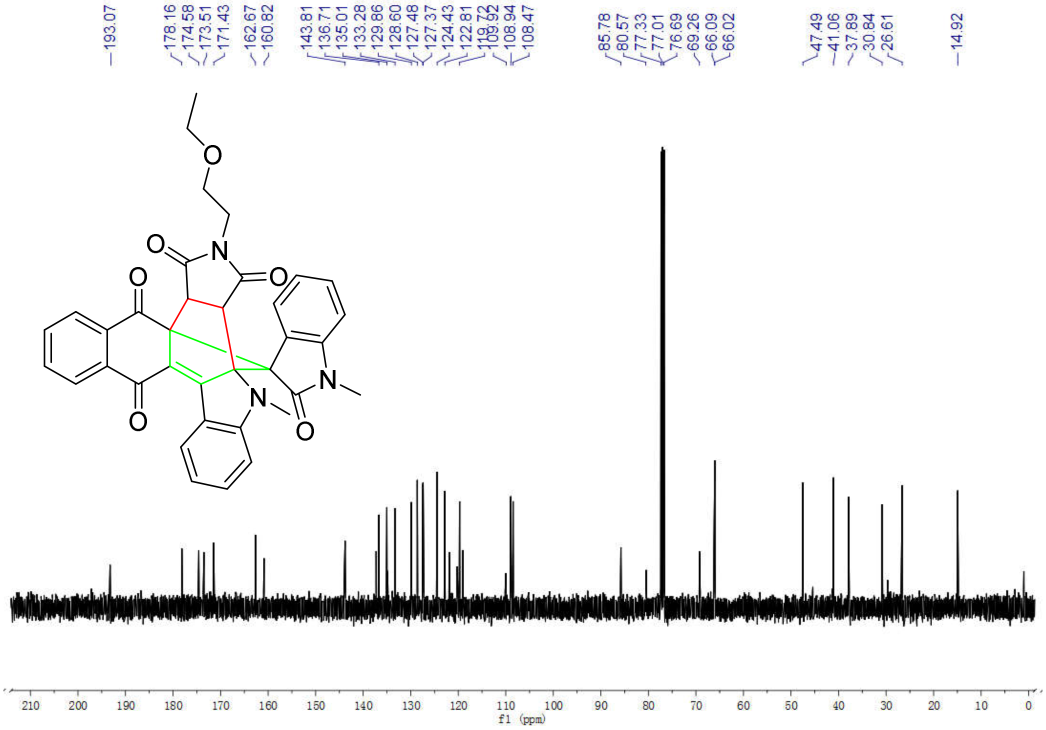

Figure S58. ^{1}H NMR (400 MHz, CDCl$_3$) spectrum of 3ax

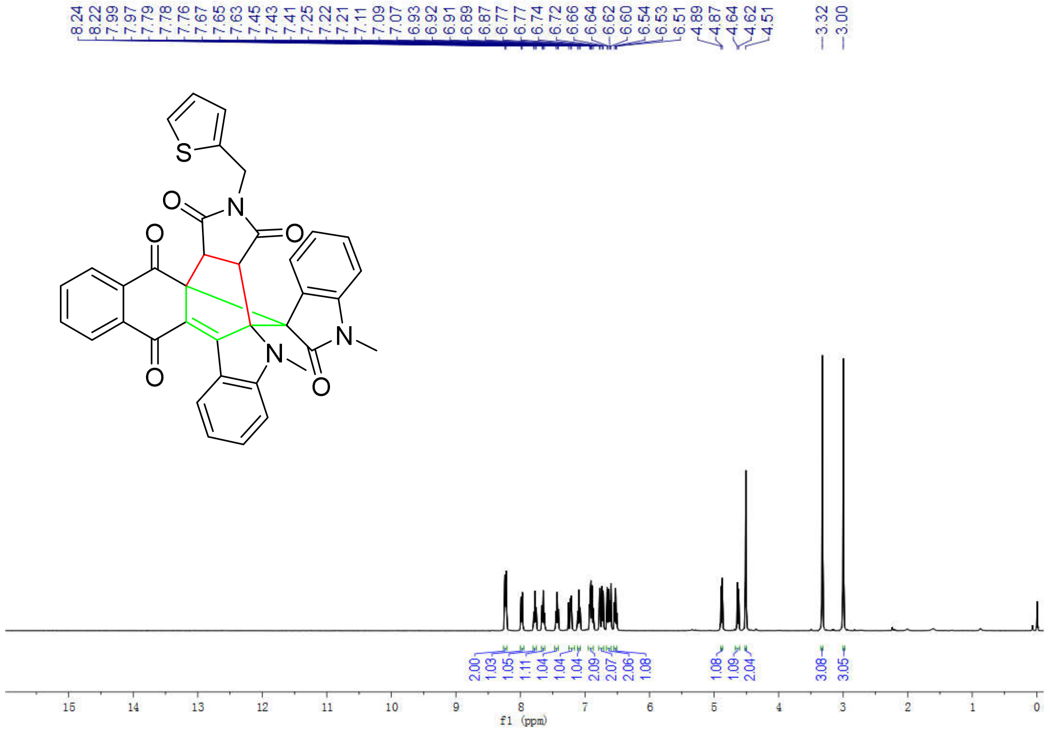

Figure S59. ^{13}C NMR (101 MHz, $CDCl_3$) spectrum of 3ax

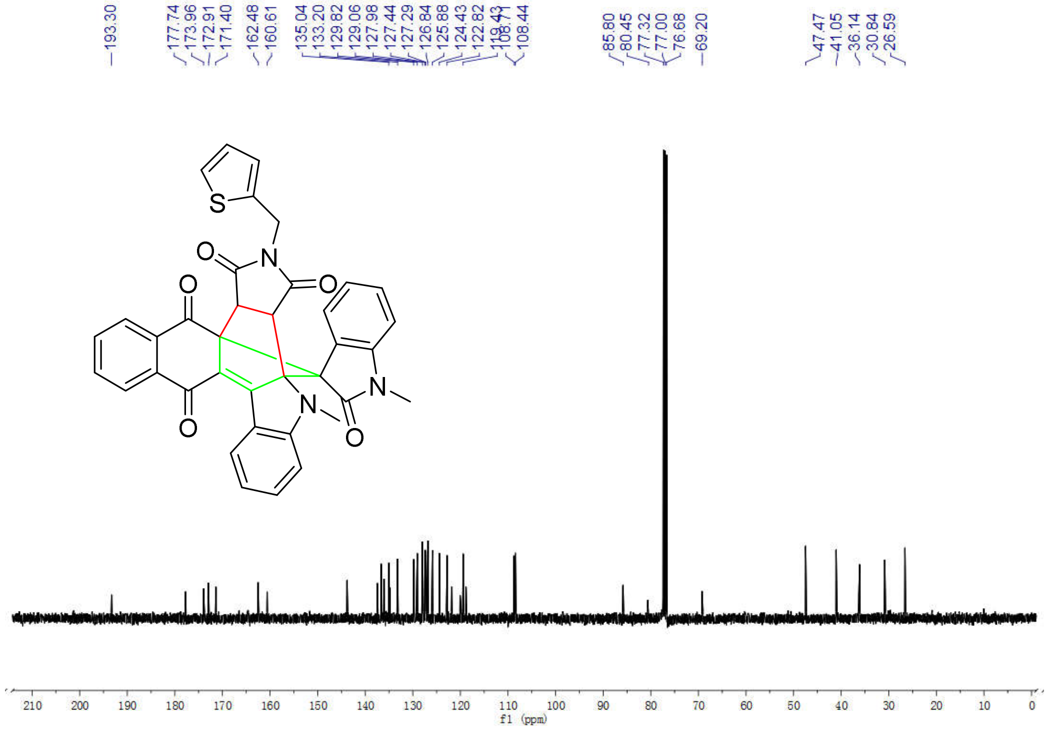

Figure S60. ^{1}H NMR (400 MHz, $CDCl_3$) spectrum of 3ay

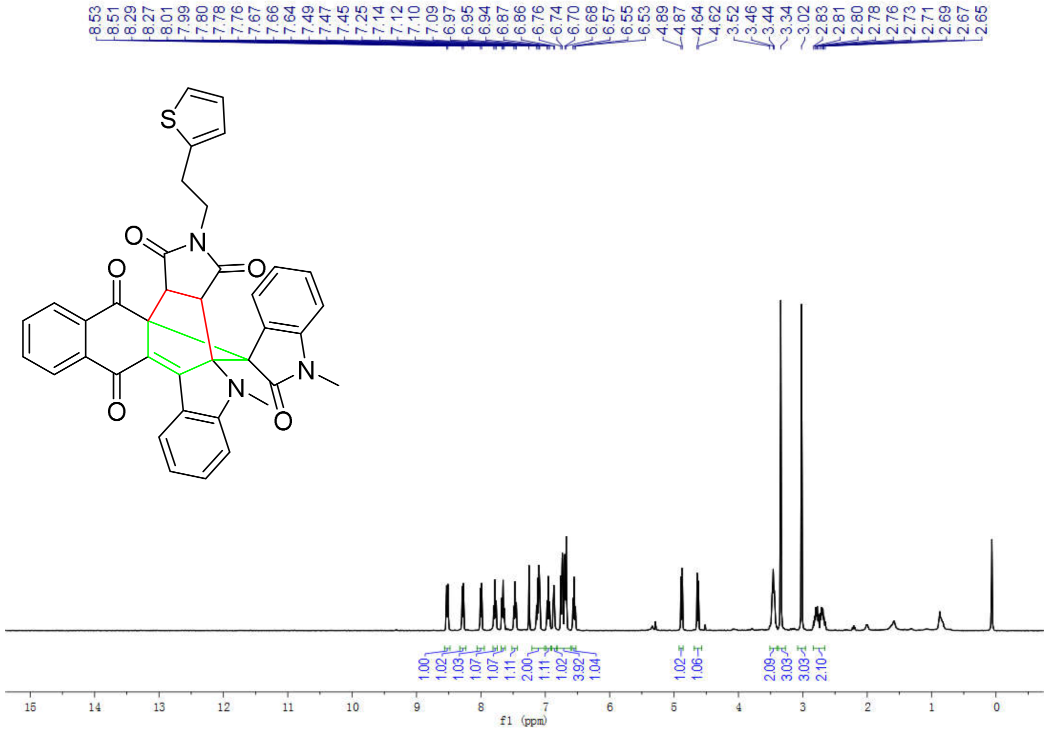

Figure S61. ^{13}**C NMR (101 MHz, CDCl$_3$) spectrum of 3ay**

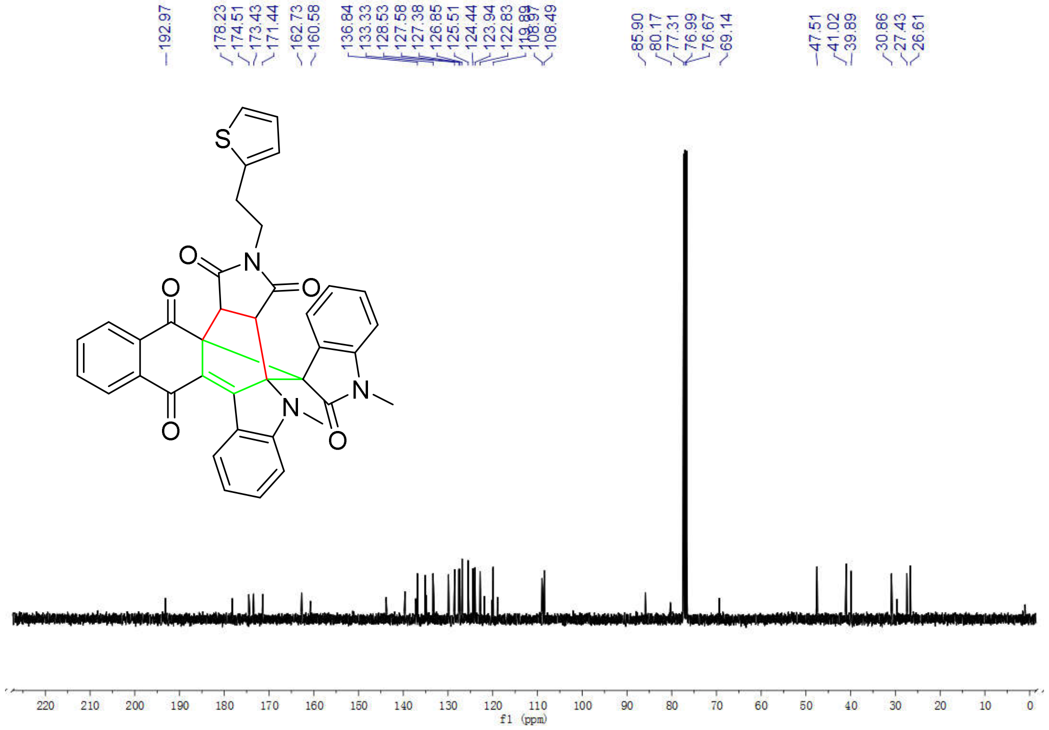

Figure S62. ^{1}**H NMR (400 MHz, DMSO-*d*$_6$) spectrum of 3az**

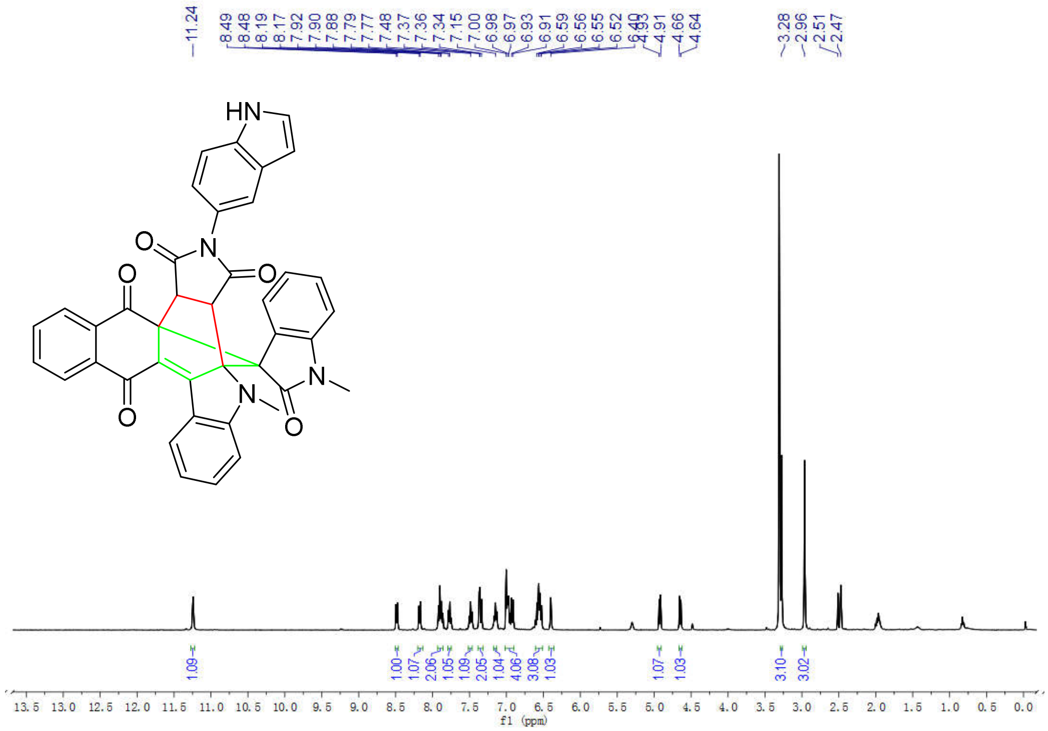

Figure S63. ^{13}C NMR (101 MHz, DMSO-d_6) **spectrum of 3az**

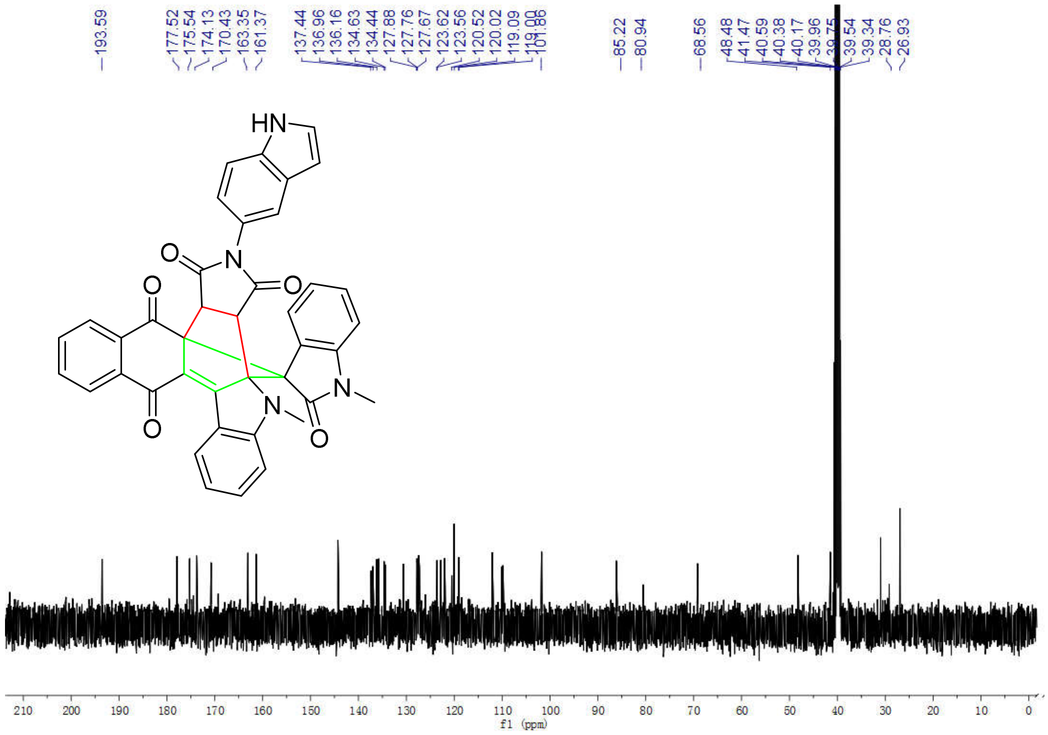

Figure S64. ^{1}H NMR (400 MHz, $CDCl_3$) **spectrum of 4a**

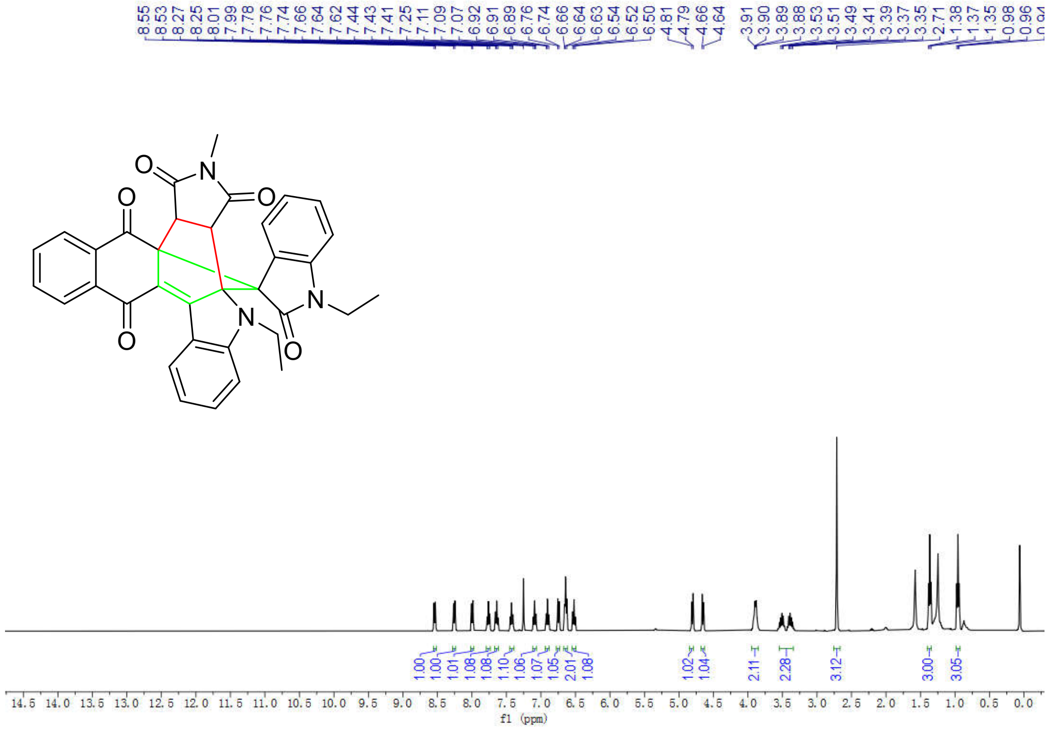

Figure S65. ^{13}**C NMR (101 MHz, CDCl$_3$) spectrum of 4a**

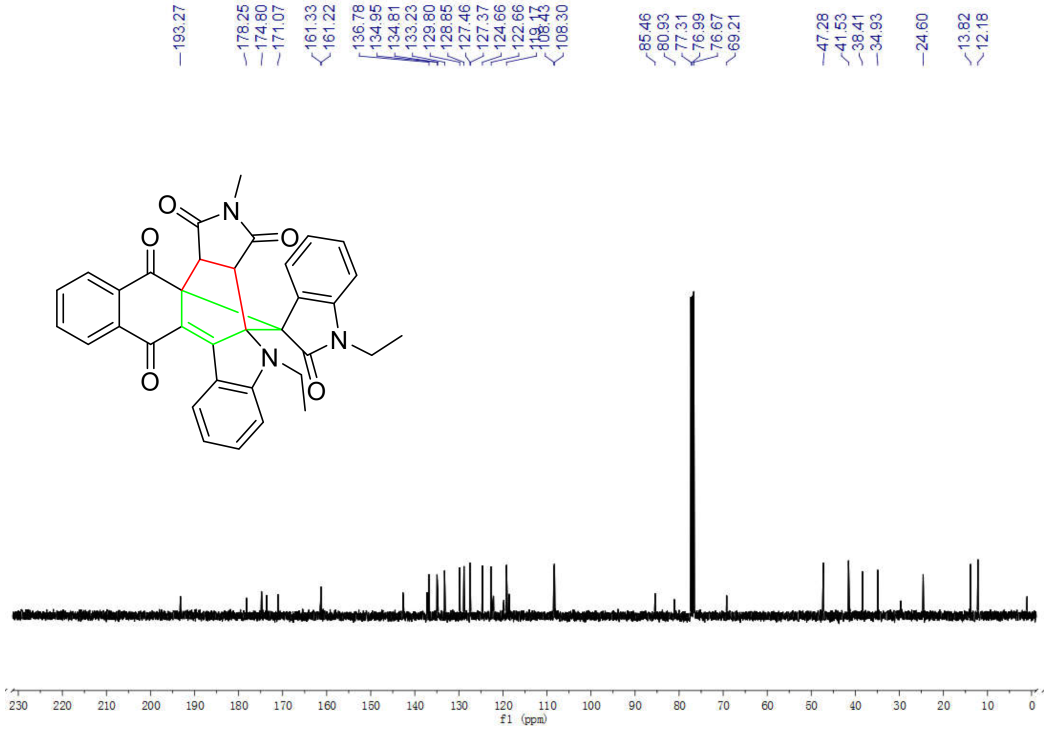

Figure S66. ^{1}**H NMR (400 MHz, CDCl$_3$) spectrum of 4b**

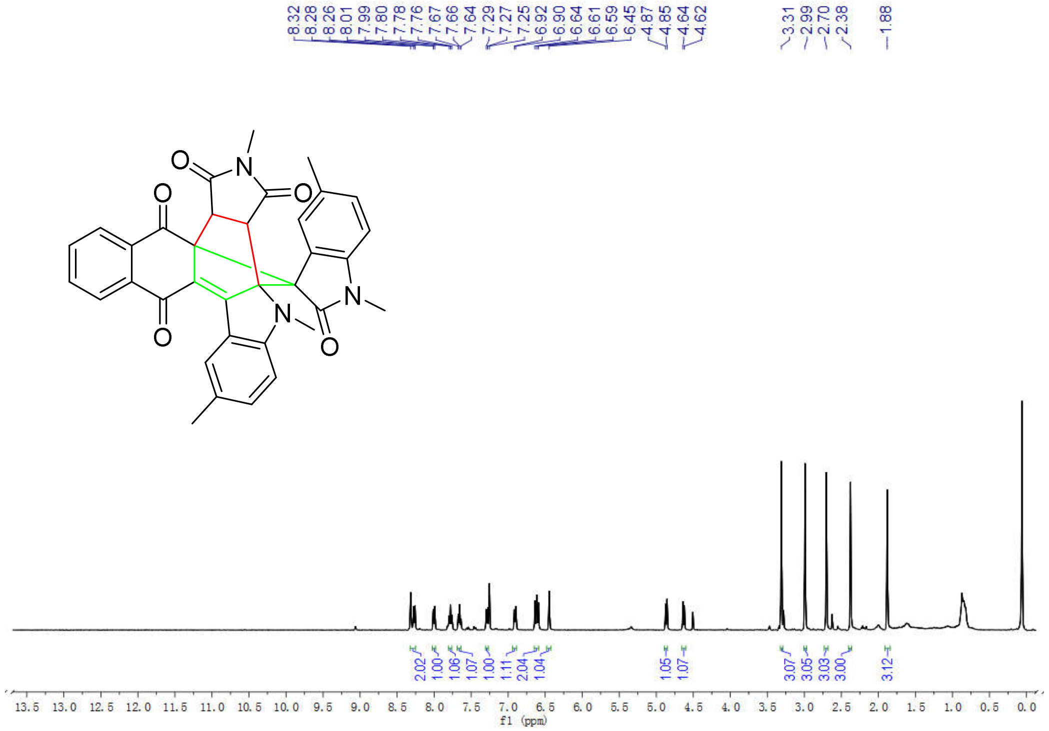

Figure S67. ^{13}C NMR (101 MHz, CDCl$_3$) spectrum of 4b

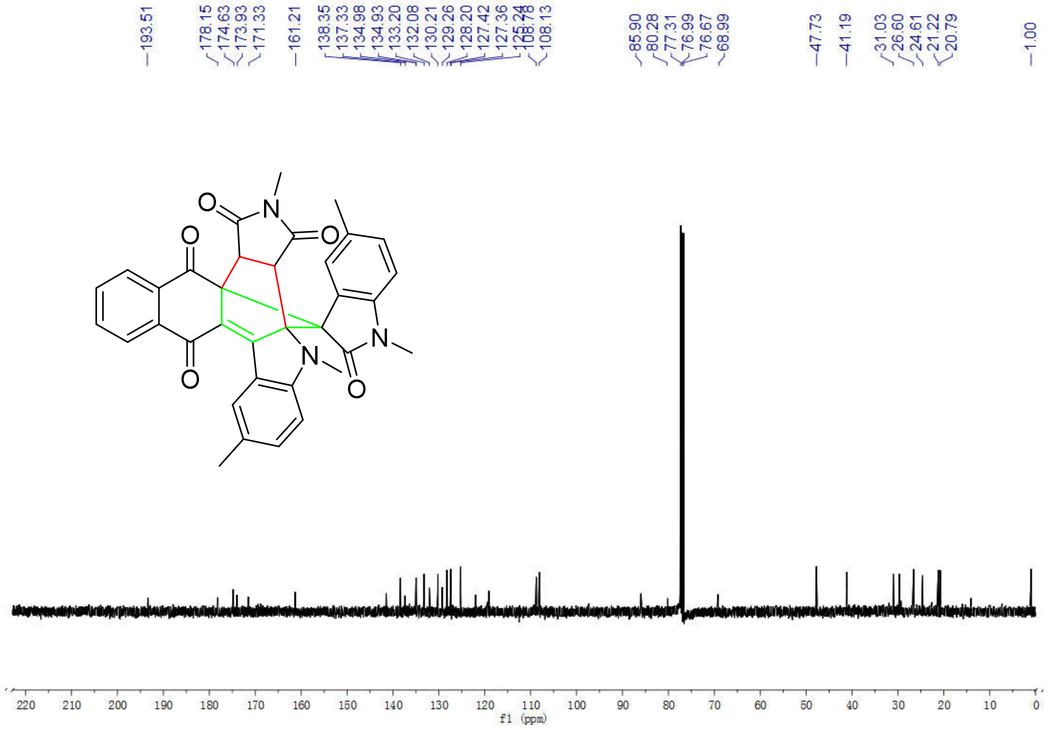

Figure S68. ^{1}H NMR (400 MHz, CDCl$_3$) spectrum of 4c

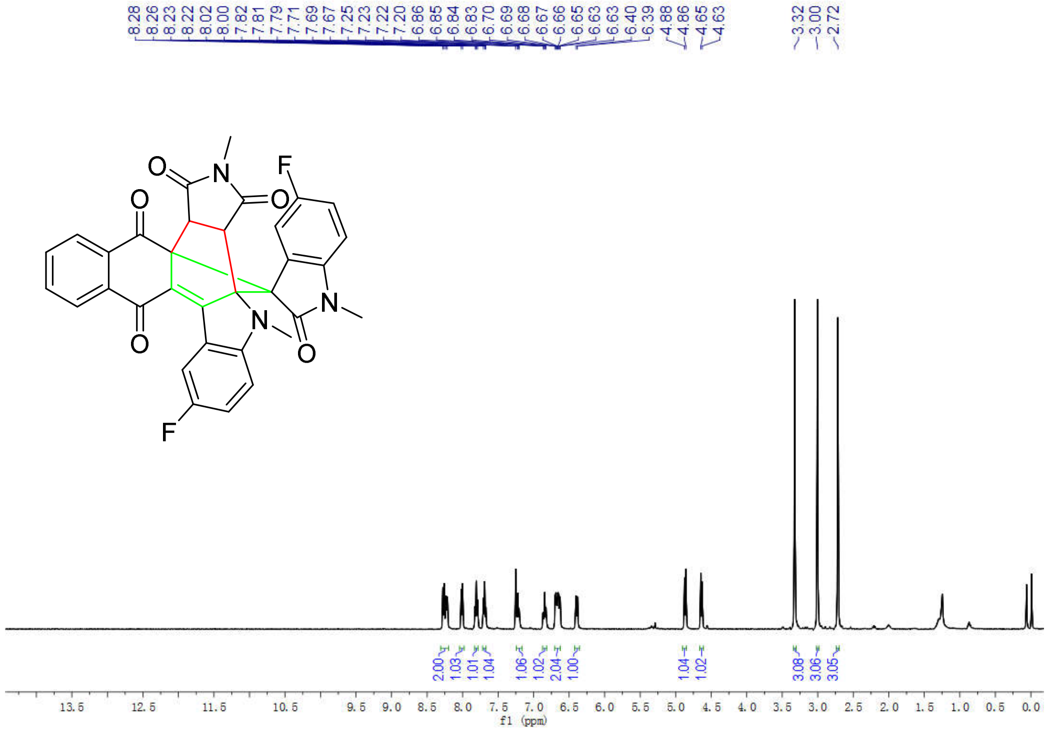

Figure S69. ^{13}C NMR (101 MHz, $CDCl_3$) spectrum of 4c

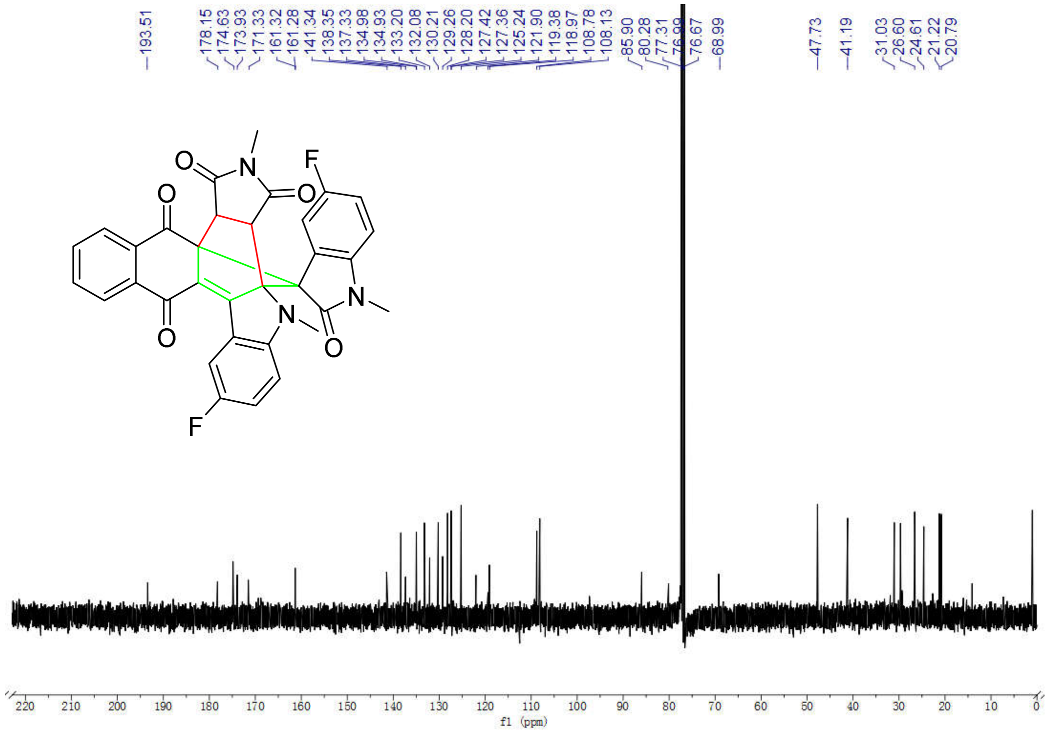

Figure S70. ^{1}H NMR (400 MHz, $CDCl_3$) spectrum of 4d

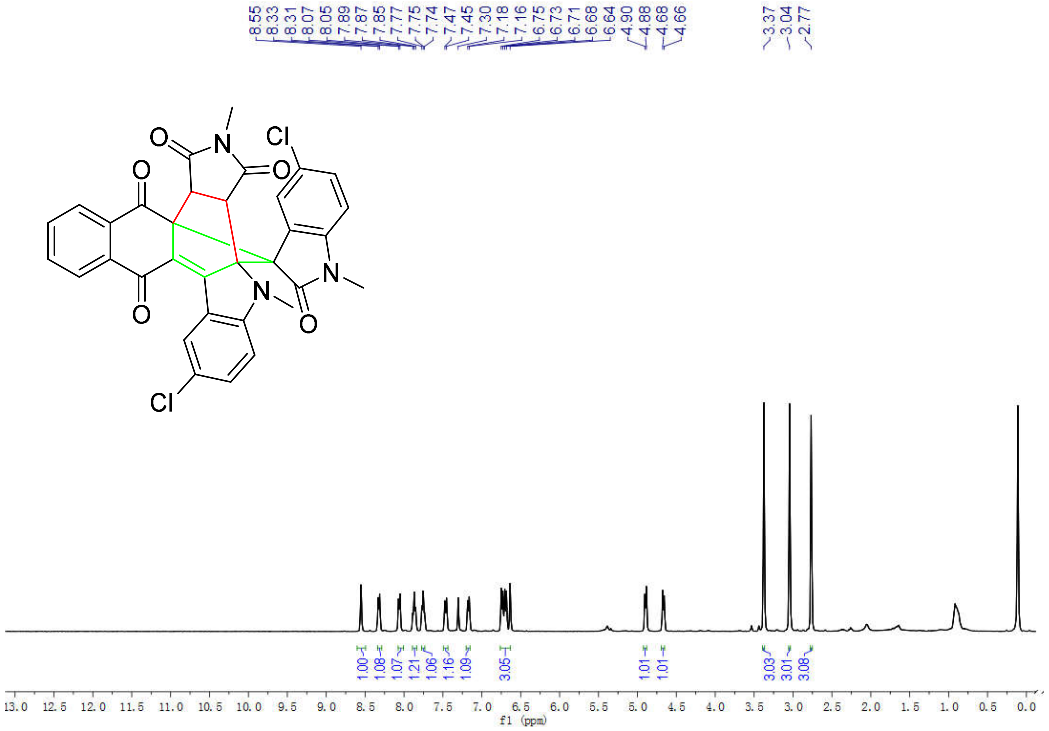

Figure S71. ^{13}C NMR (101 MHz, CDCl₃) spectrum of 4d

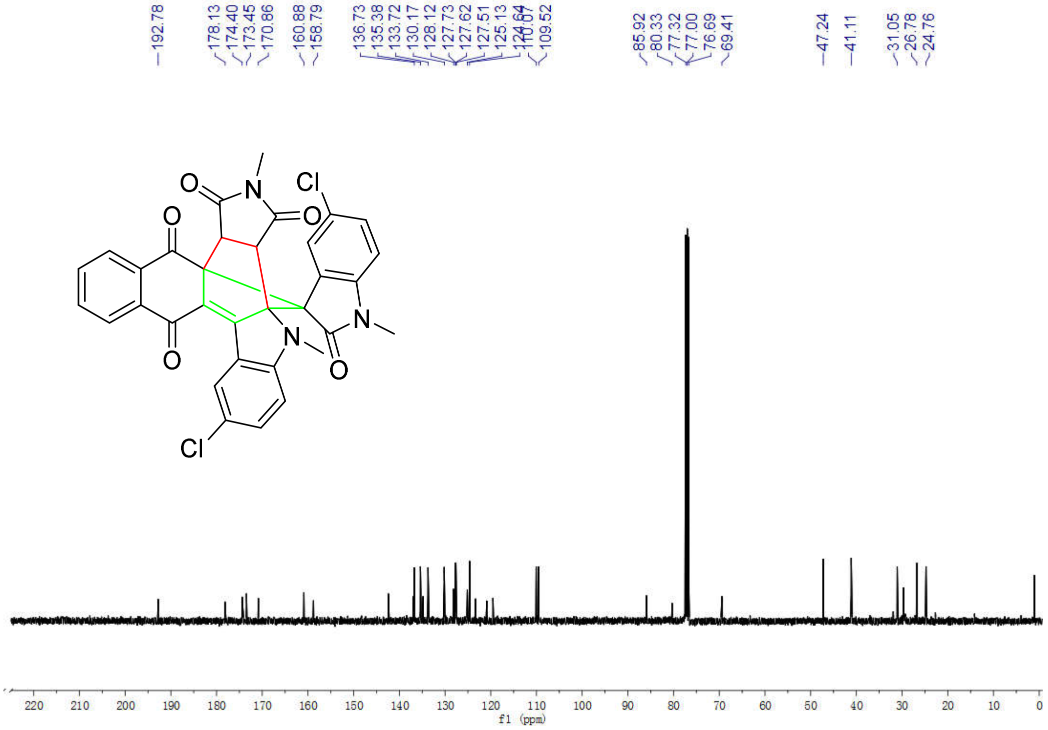

Figure S72. ^{1}H NMR (400 MHz, CDCl₃) spectrum of 4e

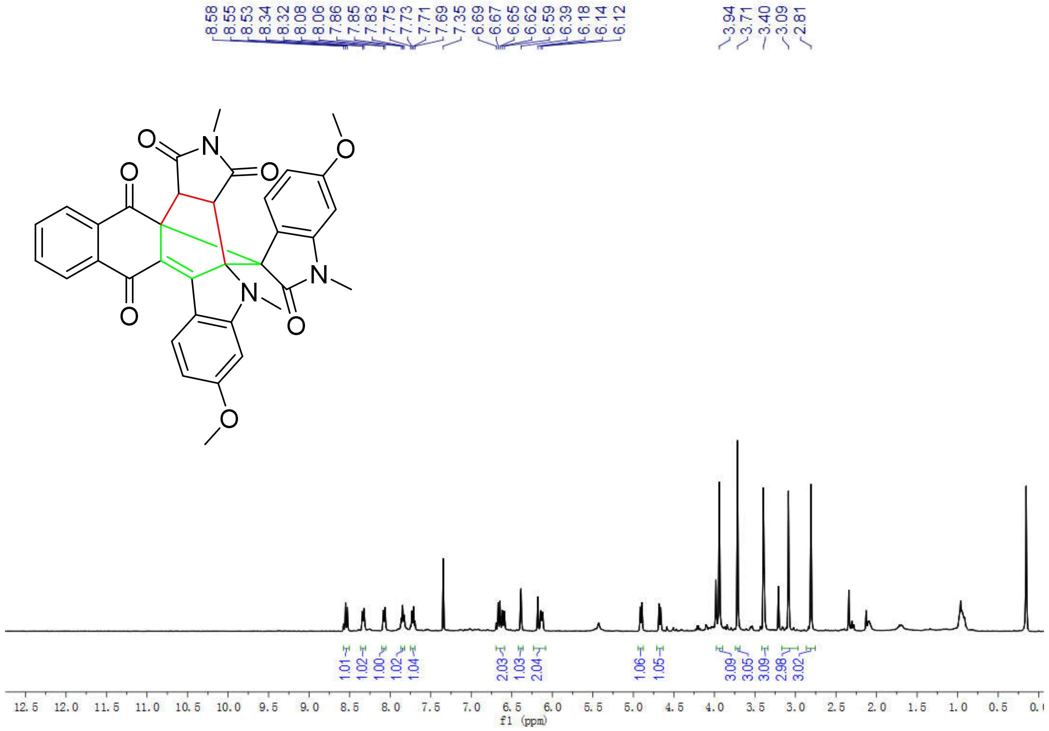

Figure S73. ^{13}C NMR (101 MHz, CDCl3) spectrum of 4e

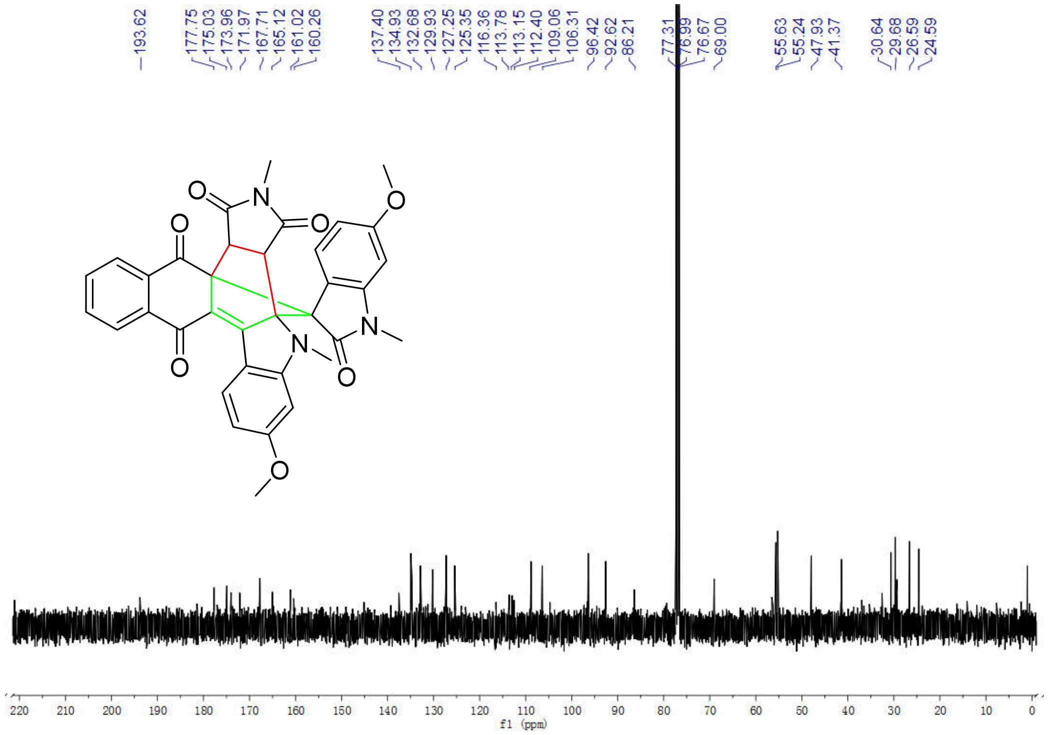

Figure S74. ^{1}H NMR (400 MHz, CDCl3) spectrum of 4f

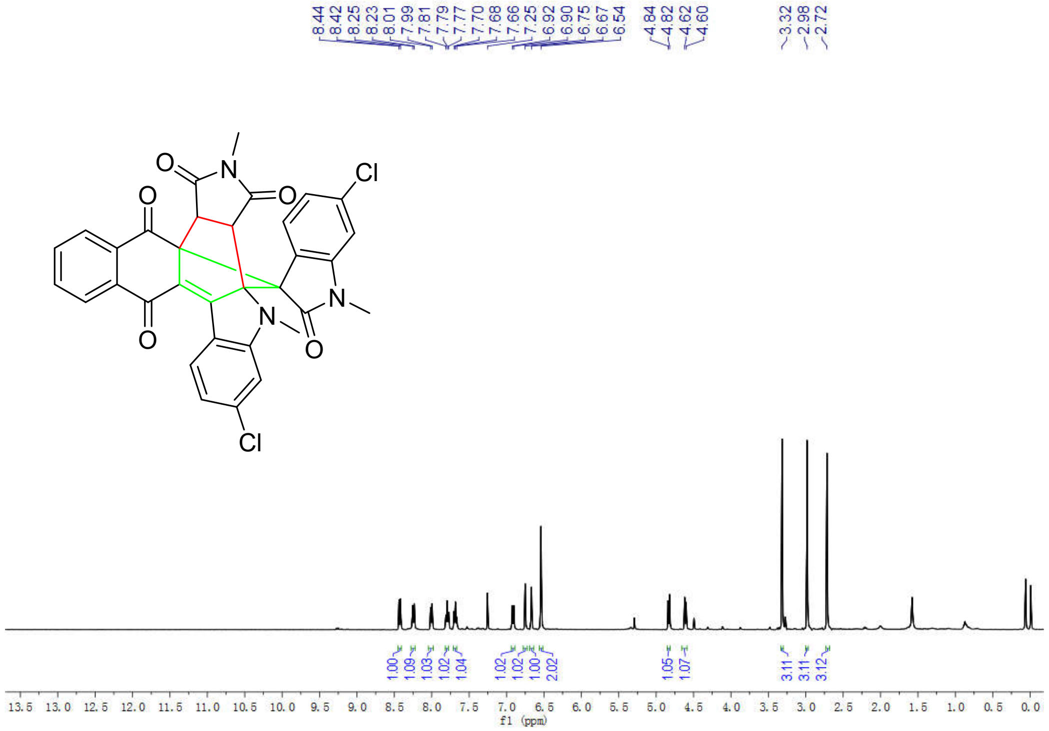

Figure S75. ^{13}**C NMR (101 MHz, CDCl$_3$) spectrum of 4f**

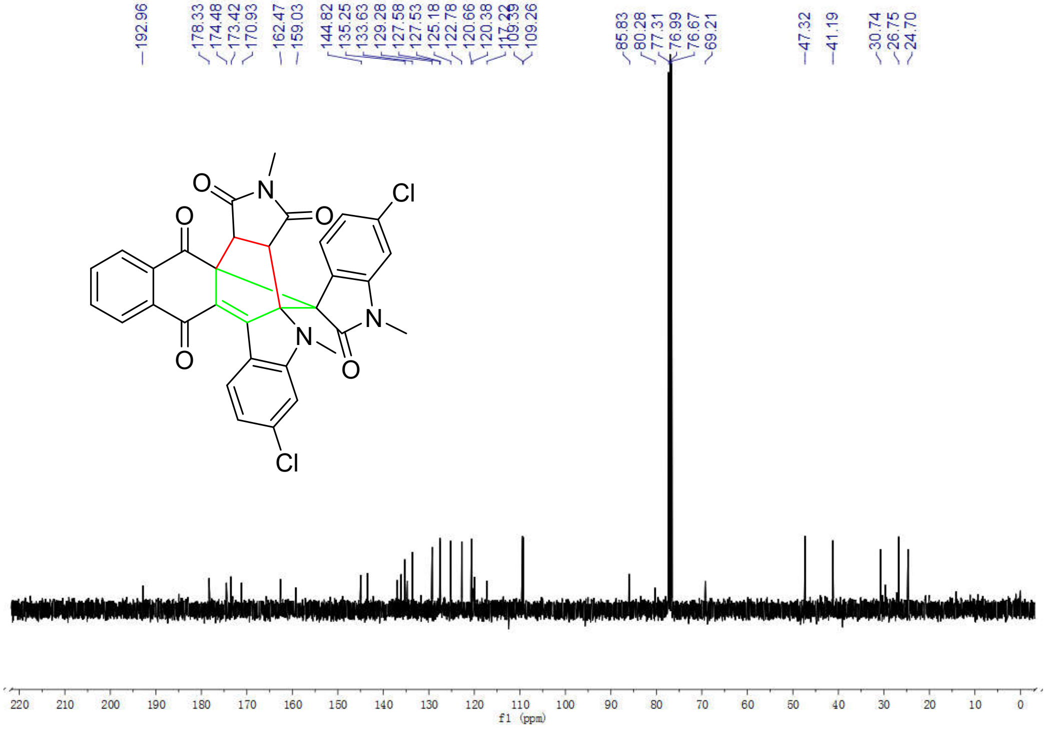

Figure S76. ^{1}**H NMR (400 MHz, CDCl$_3$) spectrum of 4g**

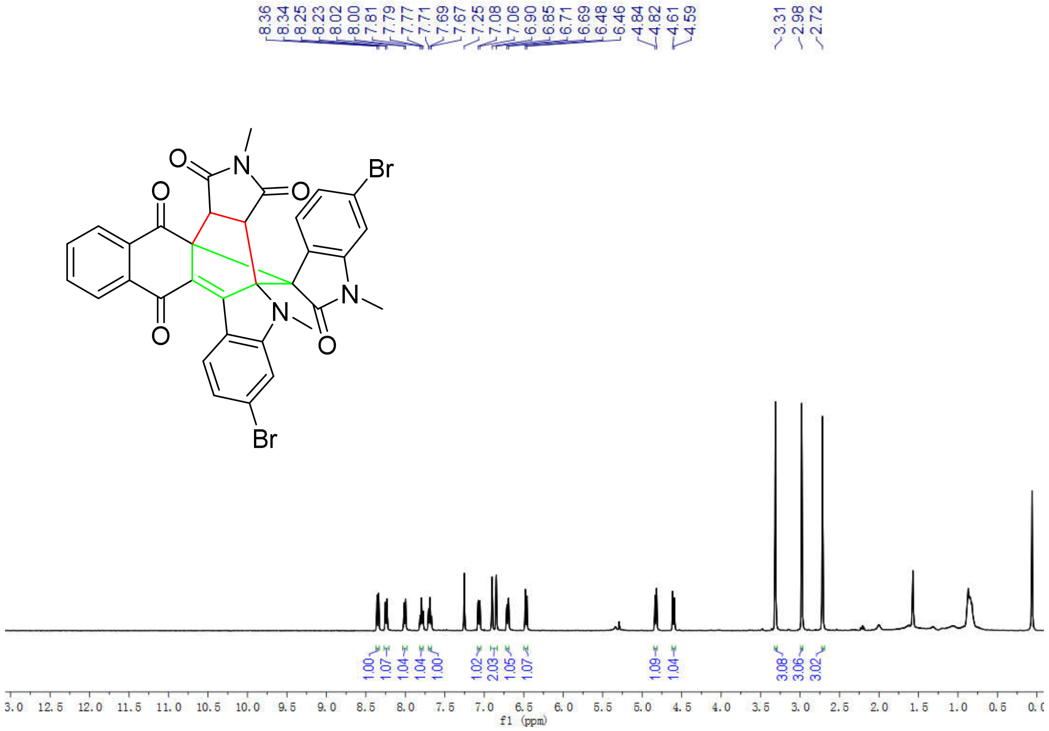

Figure S77. ^{13}C NMR (101 MHz, CDCl$_3$) spectrum of 4g

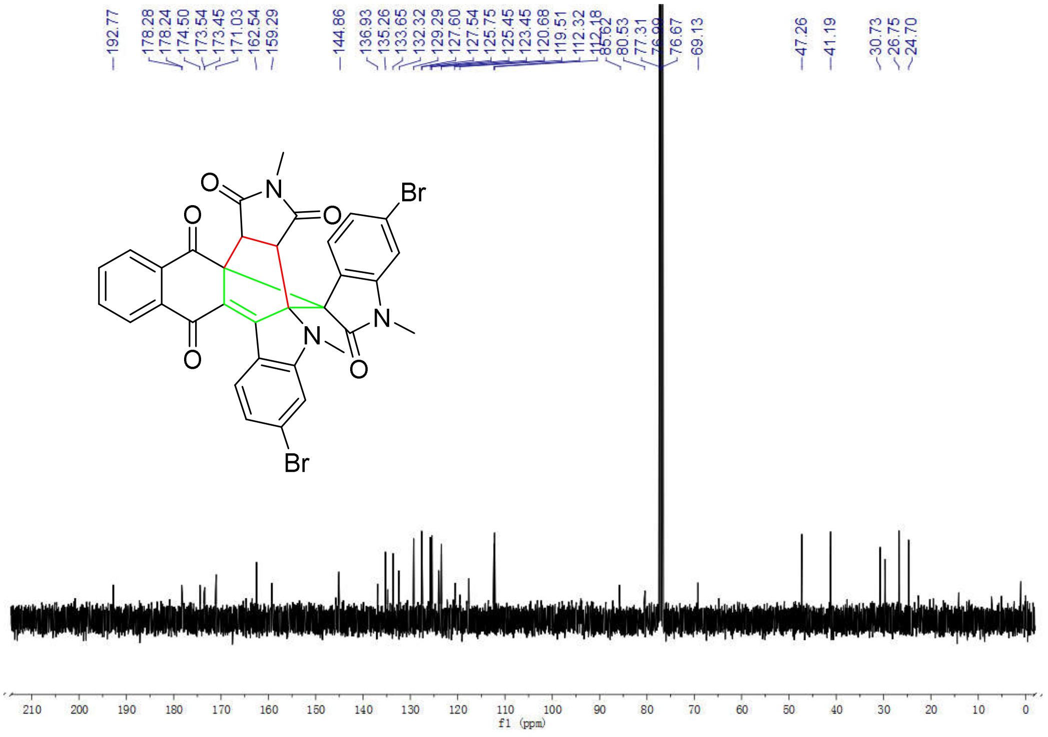

Figure S78. ^{1}H NMR (400 MHz, CDCl$_3$) spectrum of 4h

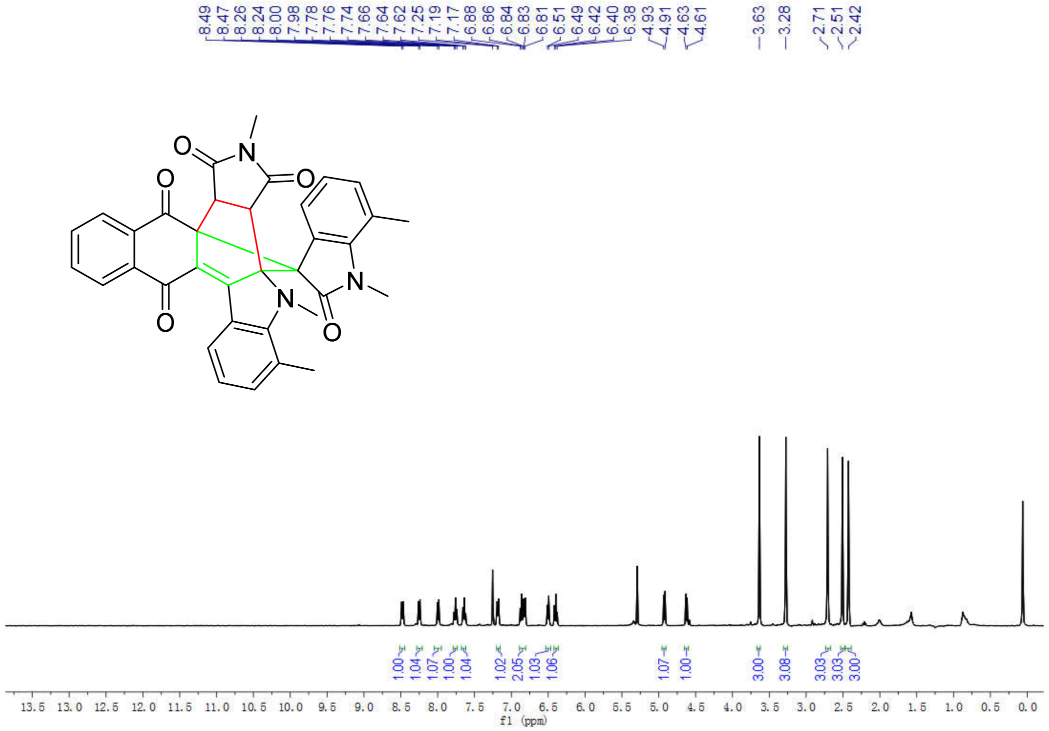

Figure S79. ^{13}C NMR (101 MHz, $CDCl_3$) spectrum of 4h

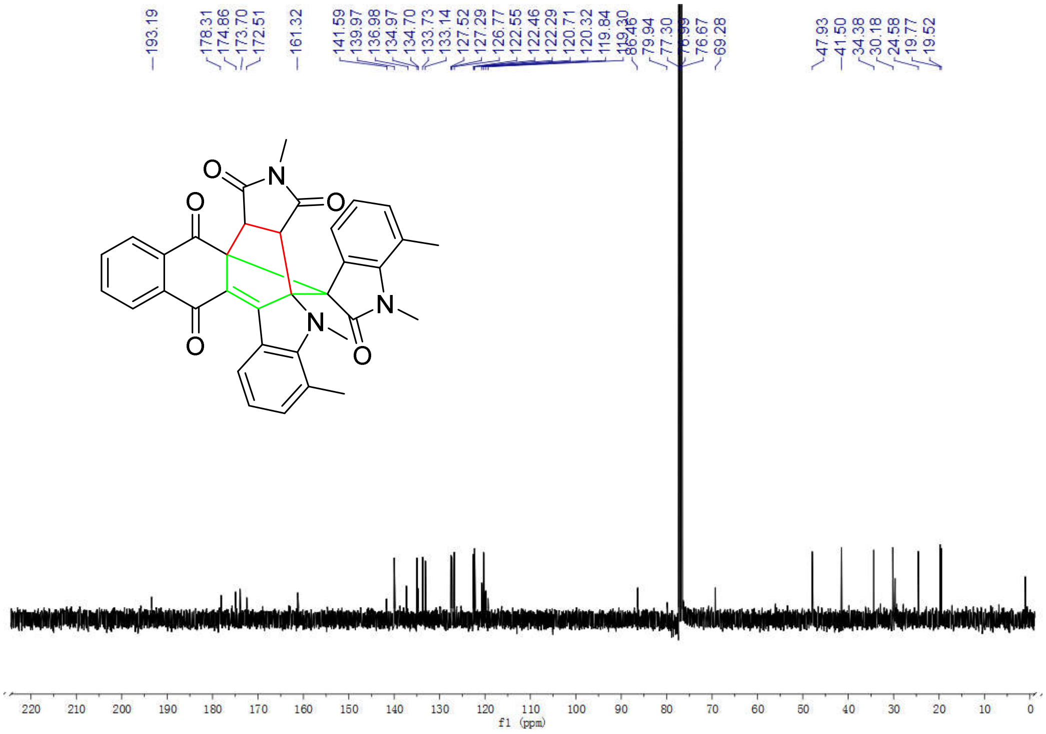

Figure S80. ^{1}H NMR (400 MHz, DMSO-d_6) spectrum of 4j

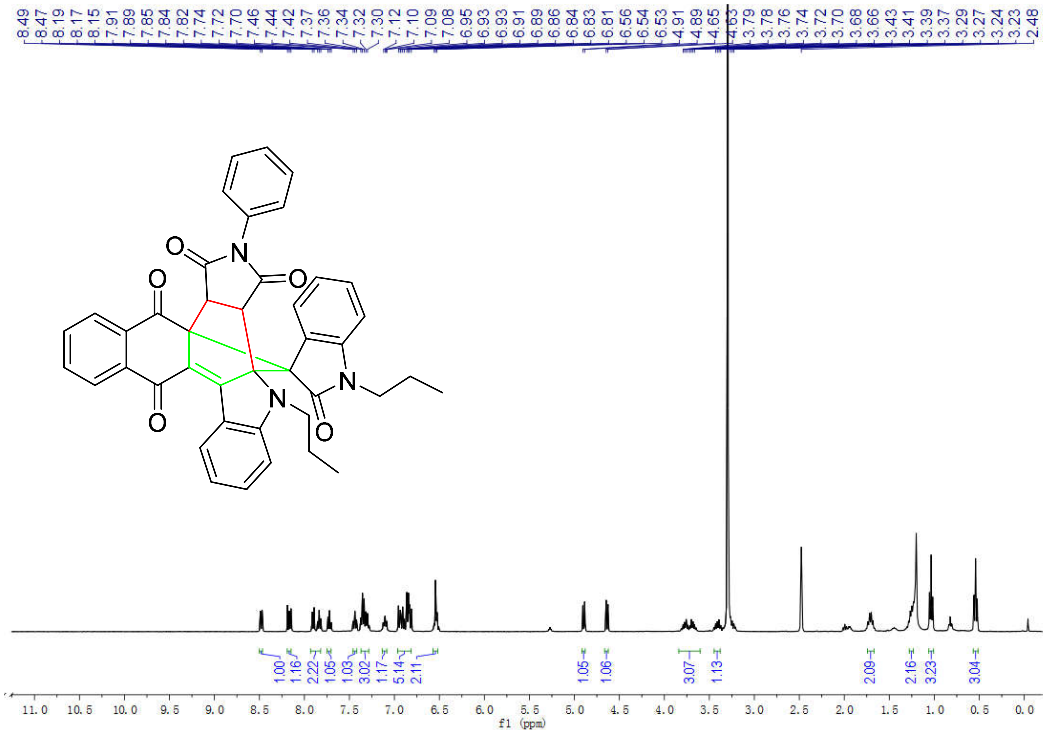

Figure S81. ^{13}**C NMR (101 MHz, DMSO-*d*$_6$) spectrum of 4j**

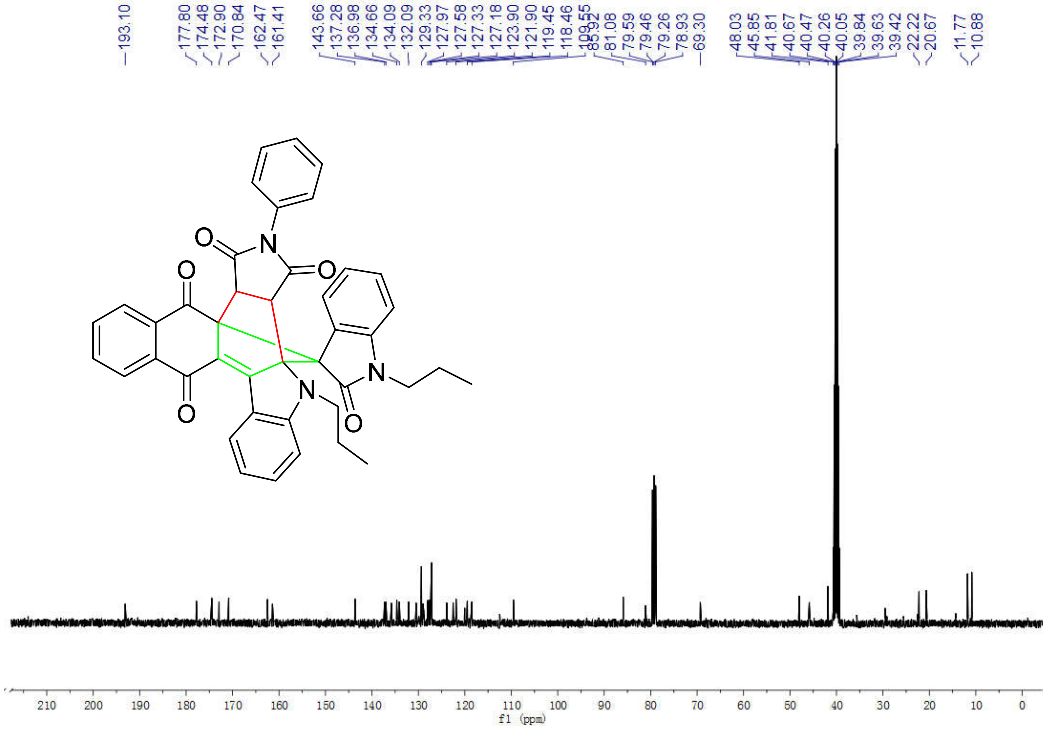

Figure S82. ^{1}**H NMR (400 MHz, CDCl$_3$) spectrum of 4k**

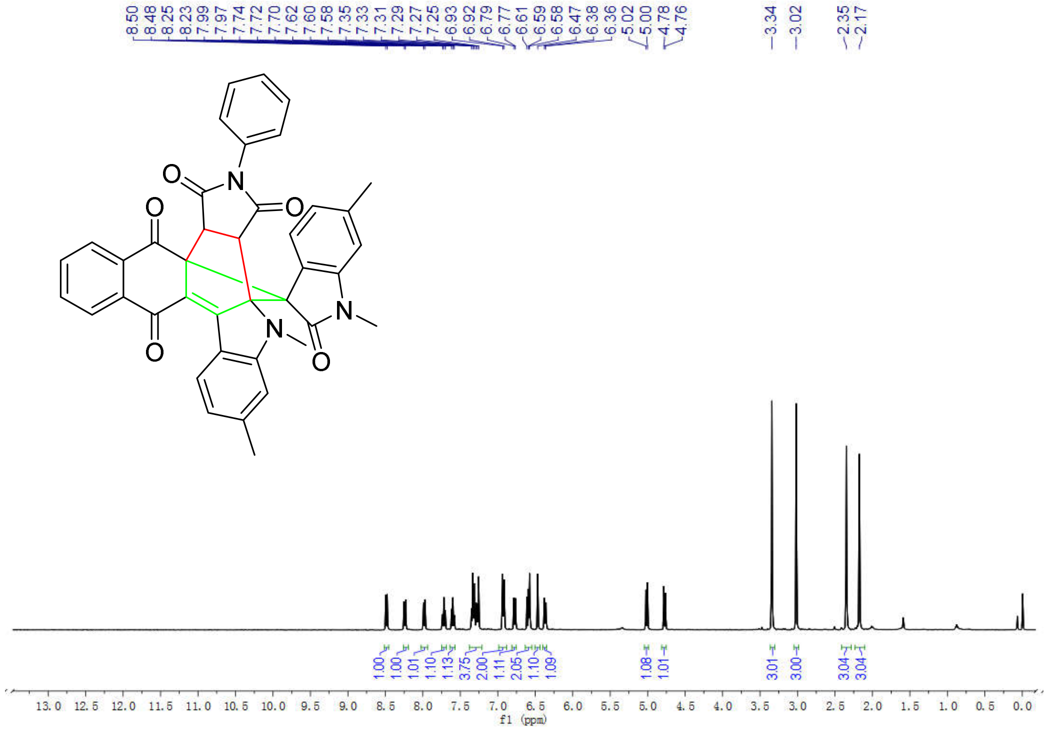

Figure S83. ^{13}C NMR (101 MHz, CDCl$_3$) spectrum of 4k

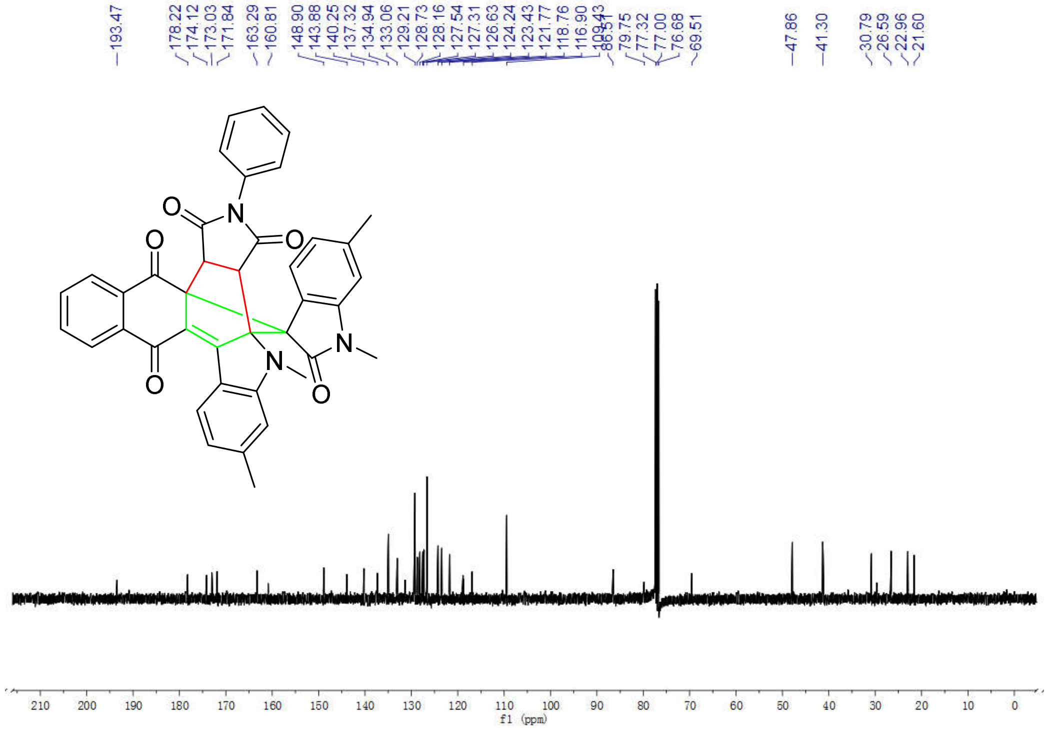

Figure S84. ^{1}H NMR (400 MHz, CDCl$_3$) spectrum of 4l

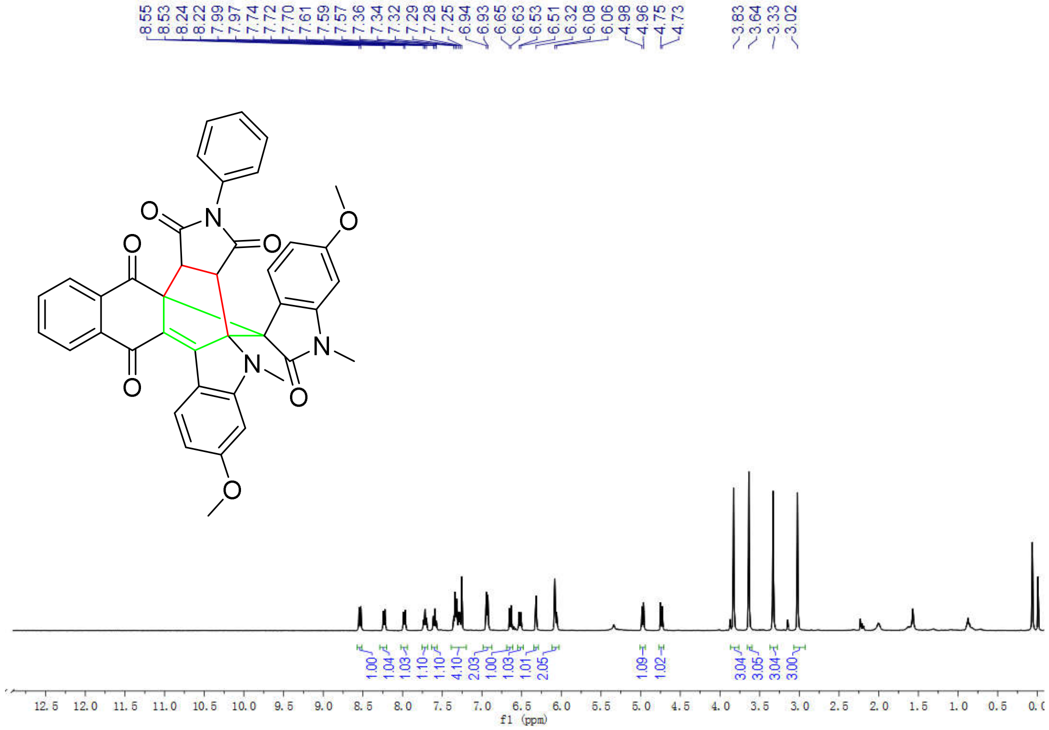

Figure S85. ^{13}**C NMR (101 MHz, CDCl**$_3$**) spectrum of 4l**

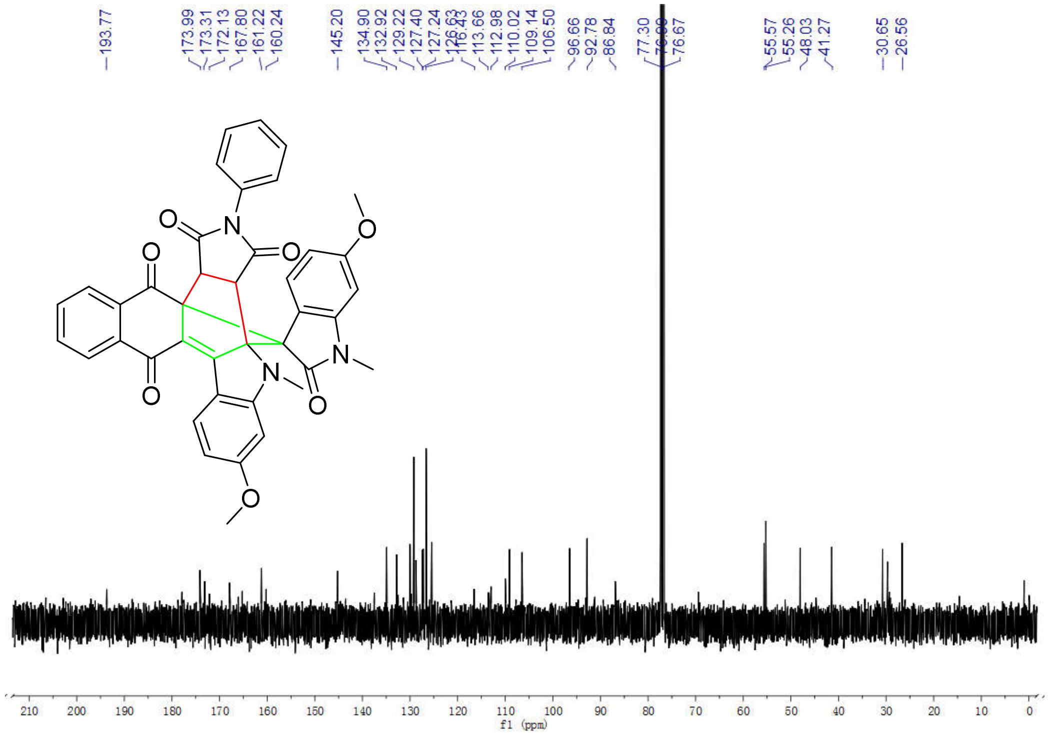

Figure S86. ^{1}**H NMR (400 MHz, CDCl**$_3$**) spectrum of 4m**

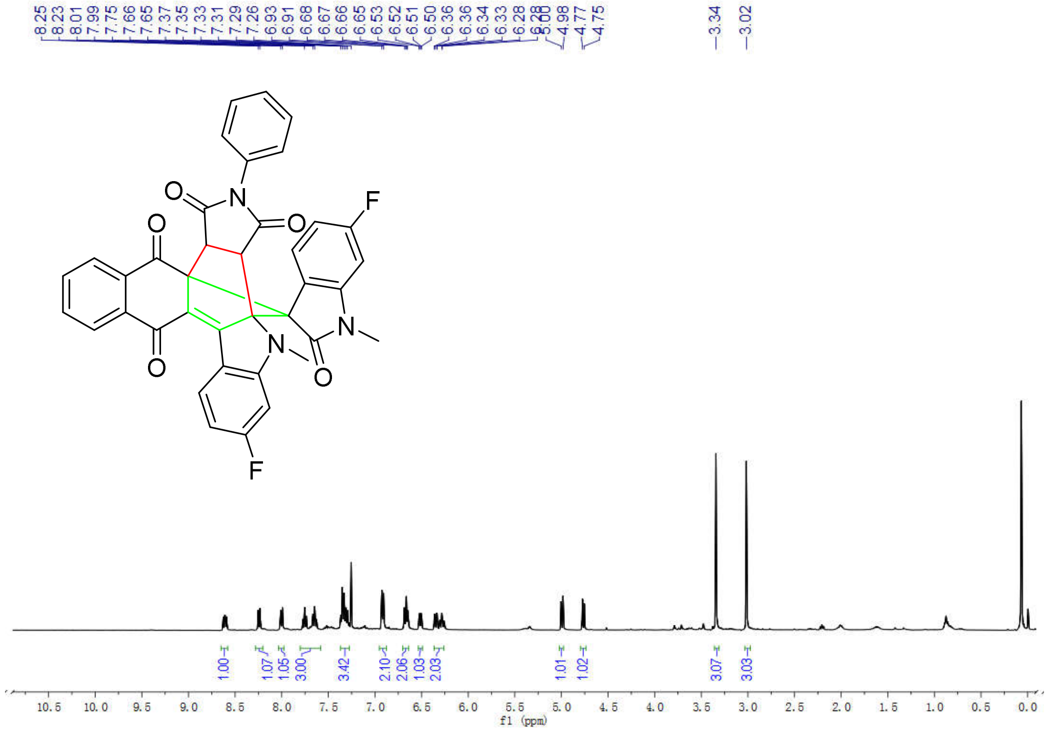

Figure S87. ^{13}C NMR (101 MHz, $CDCl_3$) spectrum of 4m

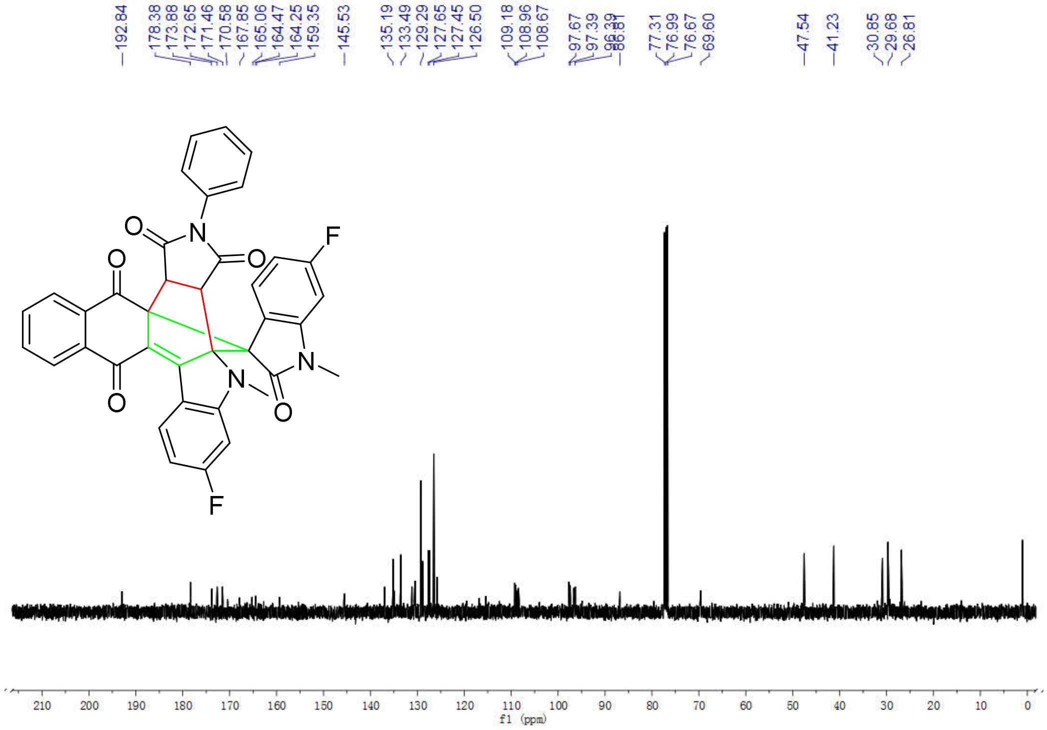

Figure S88. ^{19}F NMR (376 MHz, CDCl$_3$) spectrum of 4m

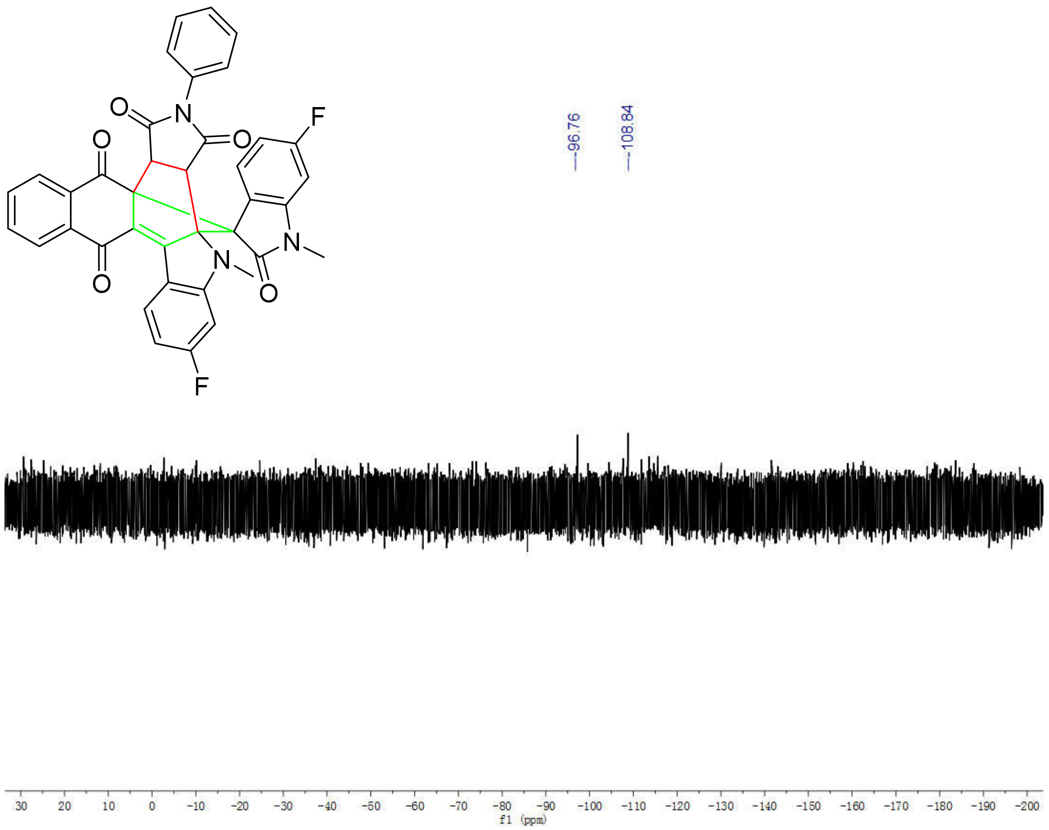

Figure S89. ^{1}H NMR (400 MHz, CDCl$_3$) spectrum of 4n

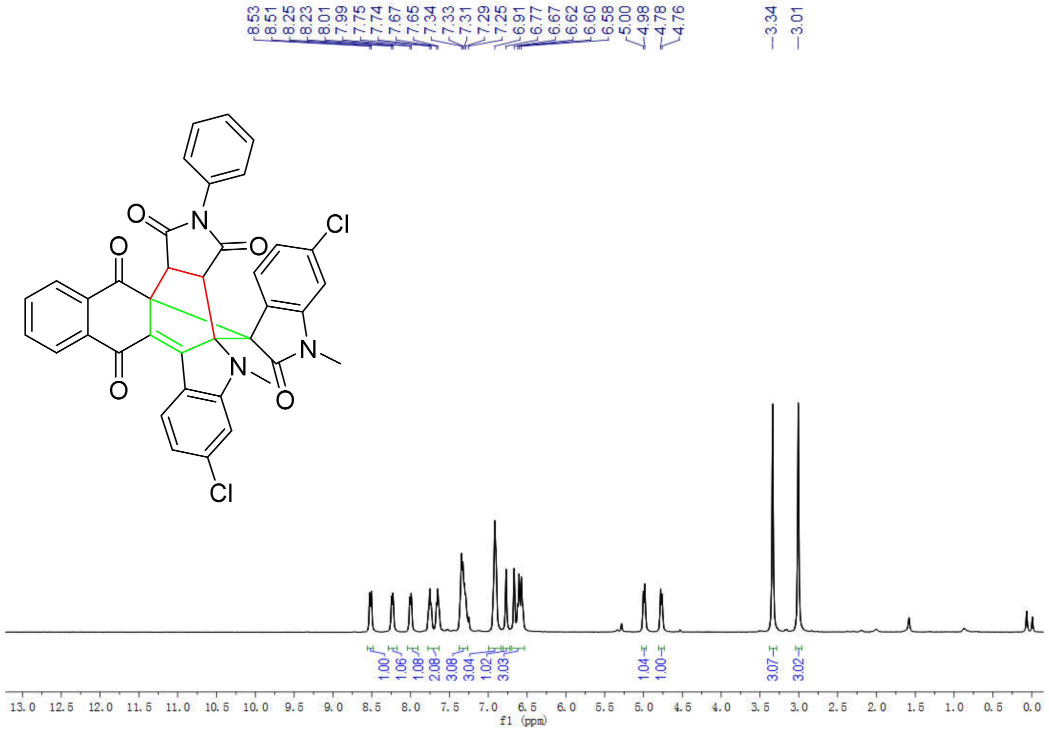

Figure S90. ^{13}C NMR (101 MHz, CDCl$_3$) spectrum of 4n

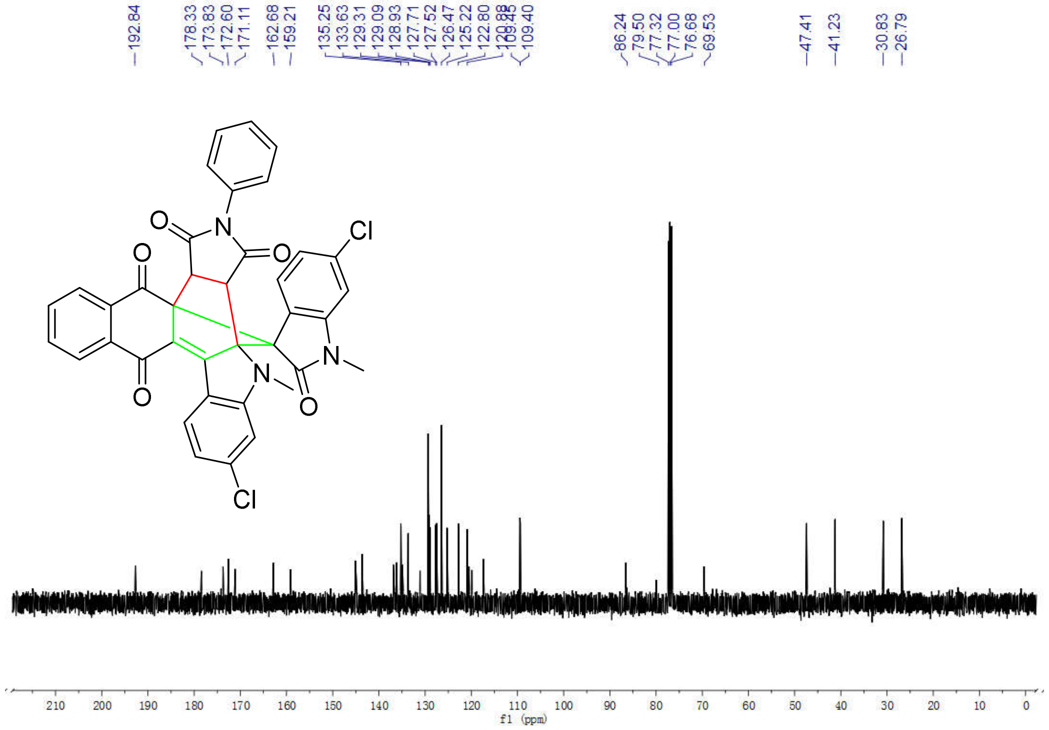

Figure S91. ^{1}H NMR (400 MHz, CDCl$_3$) spectrum of 5

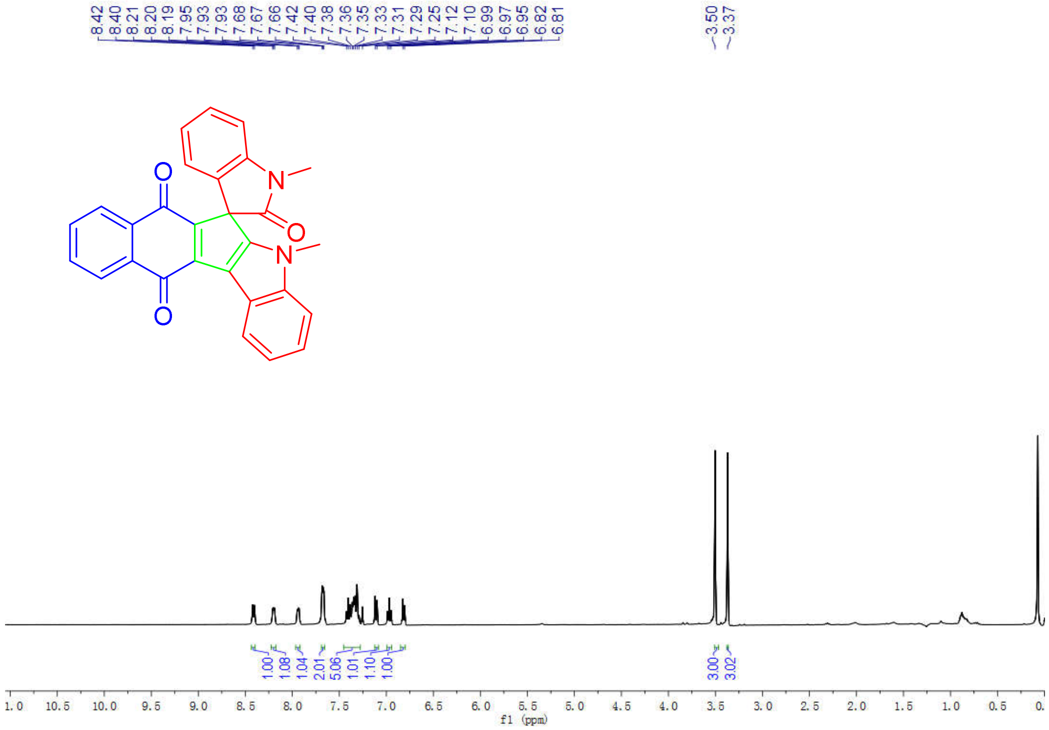

Figure S92. ^{13}C NMR (101 MHz, CDCl$_3$) spectrum of 5

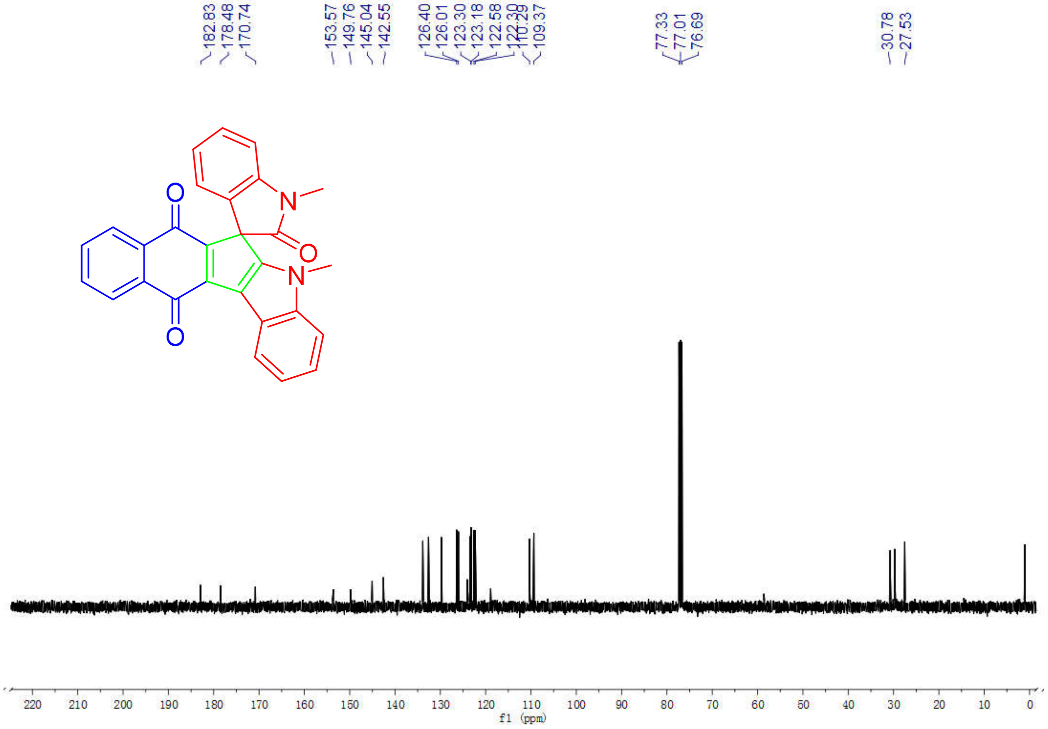

Supplement: Supplementary file 1 [file molecules-29-05639-s001.zip › molecules-3308192-supplementary.pdf]
